# Between Two Chairs: Combination of Theory and Experiment for the Determination of the Conformational Dynamics of Xylosides

**DOI:** 10.1002/chem.202201544

**Published:** 2022-08-04

**Authors:** Sven Ole Jaeschke, Thisbe K. Lindhorst, Alexander Auer

**Affiliations:** ^1^ Otto Diels Institute of Organic Chemistry Christiana Albertina University of Kiel Otto-Hahn-Platz 3–4 24118 Kiel Germany; ^2^ Max-Planck-Institut für Kohlenforschung Kaiser-Wilhelm-Platz 1 45470 Mülheim an der Ruhr Germany

**Keywords:** computational chemistry, conformational equilibria, monosaccharide conformation, NMR spectroscopy, xylopyranoside switches

## Abstract

The conformational properties of monosaccharides constitute fundamental features of oligosaccharides. While the energy landscape of monosaccharides can be altered by a specific biochemical environment or by chemical modifications, the analysis of resulting dynamic conformational equilibria is not feasible by experimental means alone. In this work, a series of β‐d‐xylopyranosides is used to outline how a combination of experimental NMR parameters and computed molecular properties can be used to determine conformers and quantify the composition of conformational equilibria. We demonstrate that identifying the most stable conformers using energy calculations is challenging and computing of NMR shieldings is typically not sensitive enough. On the other hand, computed spin‐spin coupling constants for the xyloside ring can be used to unambiguously assign experimental NMR data of dynamic conformational equilibria and quantify the ratio of different conformers in the mixture. As a proof of principle, this procedure allowed to analyze a hitherto unknown dynamic equilibrium of a diamino‐xyloside as a precursor of a molecular switch.

## Graphical abstract

**The combination of experimental** and computed data allows to disentangle and quantify complex dynamic conformational mixtures of xylosides. Computed spin‐spin coupling constants bridge the gap between experimental NMR data and the conformer distribution.

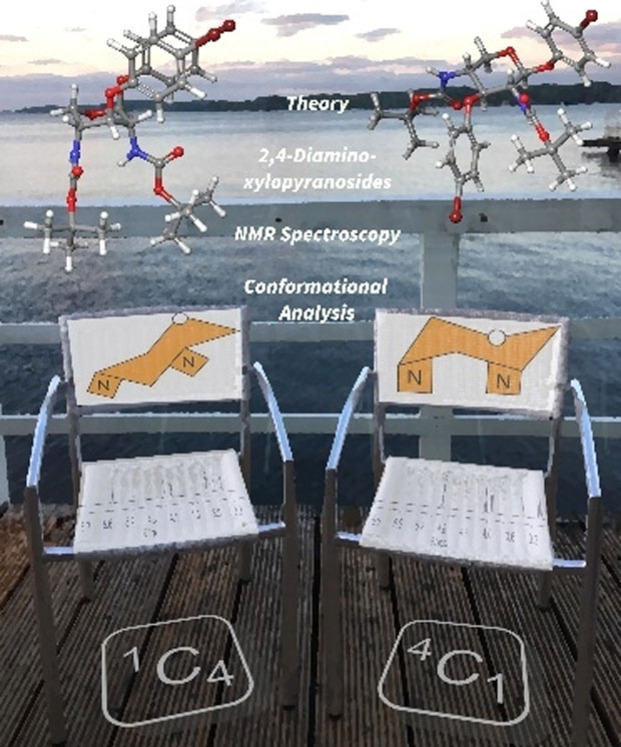

## Introduction

Oligosaccharides mediate a vast amount of biological functions based on their structural characteristics.^[1]^ These include the specific configuration of monosaccharide constituents, the stereochemistry of the glycosidic linkages and, most importantly, the conformational features of oligosaccharides. The conformational flexibility and dynamics have been extensively studied using MD simulations with carbohydrate‐optimized force fields in order to gain insight into the biological consequences of the sugar decoration of glycoconjugates.^[2]^ However, the conformational study of oligosaccharides has frequently emphasized the geometry of the glycosidic bond, whereas the monosaccharide constituents have mainly been considered as rigid blocks as they often maintain a fixed chair conformation. In fact, also the conformation of monosaccharides can be dynamic as sugar rings can adopt different shapes called puckers.

Sugar puckering can be altered by a specific biochemical environment or by chemical modification. Over the past years, it has been increasingly addressed in order to understand, for example, the outcome of glycosylation reactions,^[3]^ carbohydrate ring geometry along “catalytic itineraries” of glycoside hydrolases,^[4]^ or the unusual conformation of glycosaminoglycan constituents (GAGs).^[5]^ Furthermore, the control of monosaccharide conformation has been employed to design conformational switches, which can be reversibly turned between a ^4^ *C*_1_ and a ^1^ *C*_4_ conformation in a controlled way.^[6]^ In this regard, d‐xylose derivatives are frequently utilized owing to the fact that they can adopt various conformations, which might occur as equilibrium depending on the substitution pattern of the pyranose ring.[^6b^, ^6c^, ^6d^]

Typically, one of two chair conformations of a monosaccharide is the most populated one according to the energy difference between various possible other conformations and therefore, a conformational dynamics is not observed. However, monosaccharides can also exist as dynamic structural equilibria of unknown composition. Their experimental analysis is impossible when the conformational equilibria are faster than the experimental methods for their investigation.

Hence, the analysis of the conformational landscape of monosaccharides has to be advanced through a collaborative effort of synthesis, NMR analysis and computational methods.^[7]^ In this account we propose a joined experimental‐computational protocol in order to decipher complex conformational equilibria of monosaccharides based on spectroscopic data and computation of the spin‐spin coupling constants of the pyranose hydrogen atoms. A requirement for this approach is that the exact structural information of the pure conformers can be made available.

**Scheme 1:**
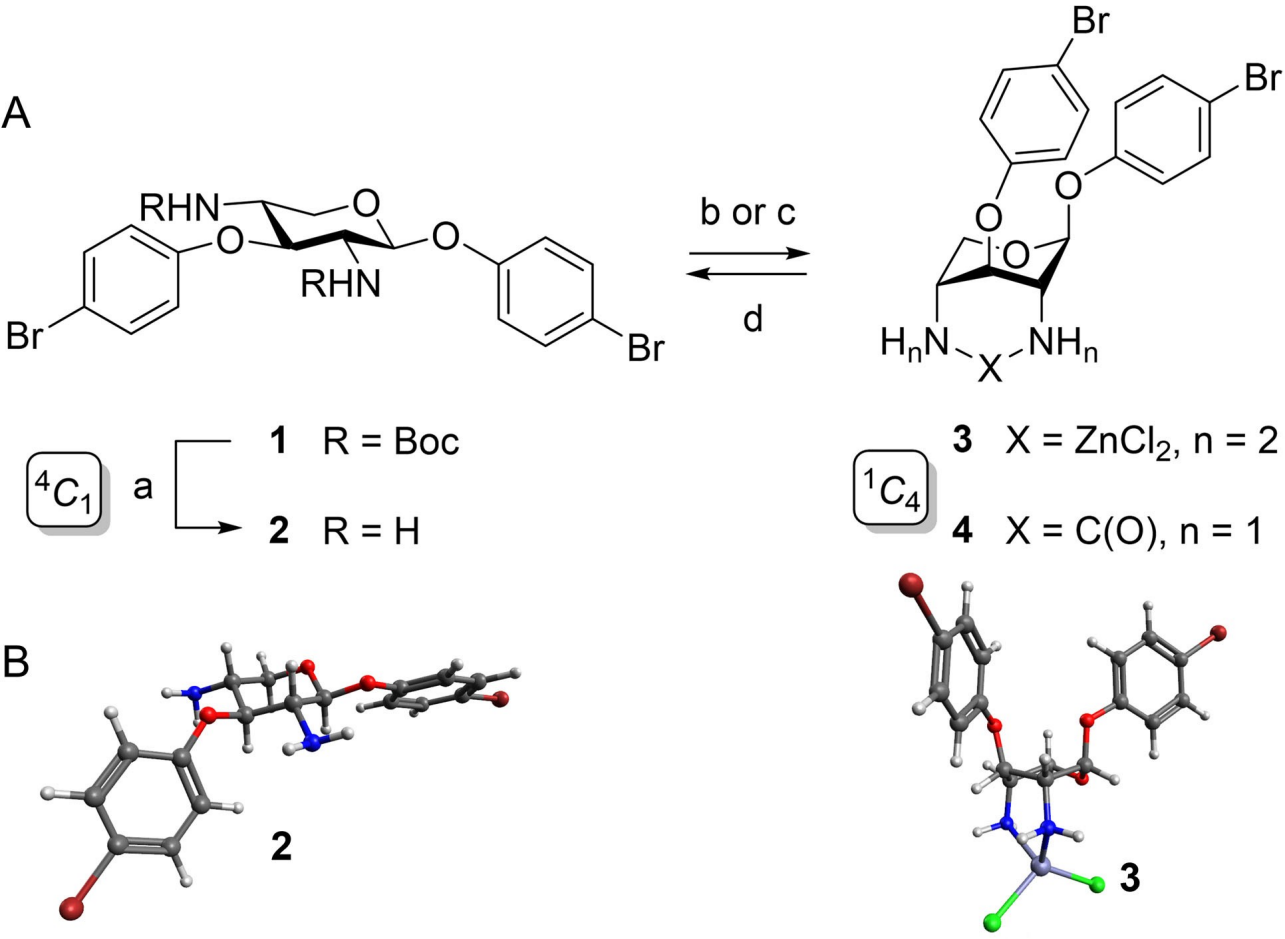
Conformation flipping of xylosides: A: The dominant ^4^ *C*_1_ conformation of xylosides **1** and **2** can be flipped into the ^1^ *C*_4_ conformation of **3** and **4** by zinc complexation or cyclic urea formation, respectively. The complexation of zinc is reversible. a) TFA, CH_2_Cl_2_, RT, 2 h, 92 %; b) ZnCl_2_, MeCN‐d_3_, RT, quant. (**3**); c) CDI, Et_3_N, DMF, RT, 16 h, 81 % (**4**); d) TETA, MeCN‐d_3_, RT, quant (**2**). Boc: *tert*‐butyloxycarbonyl; CDI: carbonyldiimidazole; TETA: triethylenetetramine. B: Optimized (B3LYP‐D4/def2‐TZVPP using CREST conformational search, see computational details) conformations of **2** (^4^ *C*_1_, left) and **3** (^1^ *C*_4_, right); structures rendered using Avogadro. Colour code: dark grey: carbon; red: oxygen; blue: nitrogen; white: hydrogen; dark red: bromine; green: chloride; grey: zinc.

Thus, we investigate four d‐xylopyranoside derivatives (Scheme 1), three of which resemble defined chair conformers (the ^4^ *C*_1_ and ^1^ *C*_4_ form, respectively) and one exists as a dynamic conformational mixture. The defined conformers serve as experimental reference points for the computational analysis of the mixture. First, the computational approach is assessed using a variety of methods in order to reproduce a set of properties of the xylosides with known conformation. In a second step, simulations with robust accuracy of properties with sufficient sensitivity can be applied in conjunction with experimental results. This two‐step procedure, as a proof of principle, is then applied to a xyloside derivative that exhibits an intriguing conformational equilibrium depending on the solvent chosen. We outline that a conformational search and computed relative energies allowed to identify the most important conformers. Furthermore, we show that the computation of the spin‐spin coupling constants of the xyloside ring protons using standard DFT methods and fitting to the experimental results allowed to quantify the composition of the so far unknown conformational mixture.

## Results and Discussion

### Xylosides as molecular switches – benchmarks for computational approaches

**Figure 1:**
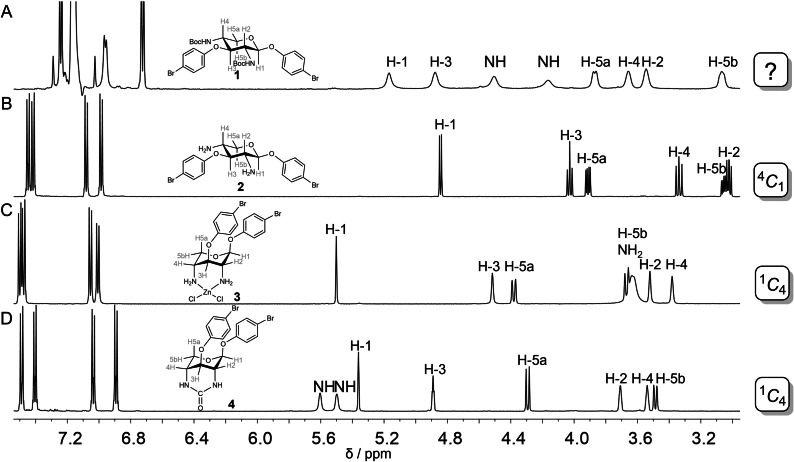
Cutouts of ^1^H NMR spectra of xylosides **1** (C_6_D_6_, 500 MHz), **2**, **3**, and **4** (CD_3_CN, 500 MHz) for conformational analysis. Coupling constants prove the ^4^ *C*_1_ conformation of the diamino xyloside **2** (B) on the one hand, and the ^1^ *C*_4_ conformation of the zinc complex **3** (C) and the cyclic urea derivative **4** (D) on the other hand. However, the broad signals in the ^1^H NMR spectrum of **1** (A) suggest a conformational dynamics, which is fast on the time scale of the NMR experiment. The reversibility of the conformational flipping between **2** and **3** can be monitored by NMR. Protons were assigned by 2D NMR spectroscopic methods (COSY and HSQC). The signals of the NH_2_ groups in **2** (spectrum B) are buried under the solvent signal (water in CD_3_CN at ∼2.17 ppm, not shown). Spectra A, B, and D were measured at 300 K, spectrum C was recorded at 328 K.

Four d‐xylopyranoside derivatives, **1** to **4**, were investigated (Scheme 1A), of which the Boc‐protected xyloside **1** and its unprotected analogue **2** were recently described en route to conformational switches.^[8]^ For the characterization of the conformational properties of the synthetic xylosides, NMR spectroscopy was applied first. The 2,4‐diamino‐xyloside **2**, according to the NMR‐spectroscopic analysis of the ^3^ *J*_H,H_ coupling constants, adopts a pure ^4^ *C*_1_ conformation (Figure 1B) in various solvents (DMSO‐d_6_, acetone‐d_6_, CDCl_3_, MeCN‐d_3_; cf. Table 1). Its precursor, the bis‐Boc‐protected xyloside **1**, on the other hand, occurs as a dynamic mixture of conformers. As the kinetic of the conformational dynamics is fast compared to the time scale of the NMR experiment, an average signal set reflecting all conformers of the equilibrium is detected in the ^1^H NMR spectrum with typical broadening of the proton signals (Figure 1A). As the different conformers could not be resolved spectroscopically (cf. Figures S3–S13), in the next step a xyloside derivative with flipped conformation was required to obtain reference data for the inverted chair (^1^ *C*_4_).

**Table 1:** NMR spectroscopic data of xylosides **2**, **3**, and **4** measured in different solvents. For the experimental determination of the ^3^ *J*_H,H_ coupling constants (in Hz), ^1^H and COSY‐DQF NMR experiments were performed at 298 K if not otherwise indicated.^[23]^ Data listed in rows shaded in grey are calculated values and obtained at the PBE/pc‐J3^[22]^ level of theory with and without implicit solvent (MeCN); MAD: maximum absolute deviation, RMSD: root‐mean‐square deviation.

Xyloside	Solvent	*J*_1,2_	*J*_2,3_	*J*_3,4_	*J*_4,5a_	*J*_4,5b_	*J*_5a,5b_	MAD	RMSD
2	DMSO‐d_6_	7.44	8.93	8.93	5.00	10.73	11.54		
2	Acetone‐d_6_	7.64	9.20	9.20	[a]	[a]	[a]		
2	CDCl_3_	7.60	9.05	9.05	4.76	9.79	11.38		
2	MeCN‐d_3_	7.52	9.06	9.06	5.03	10.22	11.66		
2 (^4^ *C*_1_)	B3LYP‐D4, PBE	7.49	9.58	9.04	5.46	9.97	11.10	0.52 ^[b]^	0.34
2 (^1^ *C*_4_)		1.31	2.07	3.08	2.45	1.41	11.91		
2 (^1^ *S*_5_)		7.24	10.63	2.26	2.55	0.93	11.52		
2 (^4^ *C*_1_)	B3LYP‐D4, PBE (MeCN)	7.61	9.50	8.79	5.62	10.91	11.16	0.69 ^[b]^	0.45
2 (^1^ *C*_4_)		1.18	2.00	2.87	2.52	1.26	12.09		
2 (^1^ *S*_5_)		2.10	0.52	8.42	5.93	13.00	10.06		
2 (^4^ *C*_1_)	ωB97M‐D4, PBE (MeCN)	7.54	9.51	8.82	5.52	10.88	11.16	0.66^[b]^	0.42
2 (^1^ *C*_4_)		1.38	2.00	3.08	2.44	1.48	12.10		
2 (^1^ *S*_5_)		2.23	0.50	8.38	5.58	12.94	10.16		
3	MeCN‐d_3_^[c]^	2.18	3.54	3.90	2.24	2.83	13.00		
3	B3LYP‐D4, PBE	1.01	1.83	2.60	3.17	1.21	12.67	1.71	1.26
	B3LYP‐D4, PBE (MeCN)	1.16	1.90	2.68	2.86	1.29	12.86	1.64	1.15
	ωB97M‐D4, PBE (MeCN)	1.16	1.72	2.69	2.62	1.48	12.82	1.82	1.14
4	MeCN‐d_3_	2.80	3.89	3.18	1.67	1.90	12.00		
4	B3LYP‐D4, PBE	1.87	3.46	4.36	1.14	2.21	11.04	1.18	0.79
	B3LYP‐D4, PBE (MeCN)	1.94	3.47	4.40	1.17	2.26	11.51	1.22	0.71
	ωB97M‐D4, PBE (MeCN)	2.07	3.41	4.38	1.07	2.44	11.58	1.12	0.71

[a] Coupling constants were not resolved. [b] Compared to experimental coupling constants determined in MeCN‐d_3_. [c] NMR experiments performed at 328 K.

In fact, the conformation of **2** can be inverted by the addition of one equiv. of ZnCl_2_. An estimate of the association energy of **2** with ZnCl_2_ at the B3LYP‐D4/def2‐TZVPP level of theory^[9]^ confirms that the reaction energy is exothermic by more than 90 kJ/mol (cf. Supporting Information for computational details). Thus, the reaction from **2** to **3** can be expected to be fast and quantitative. Indeed, the Zn^2+^ complex **3** forms instantaneously after the known complexation of the 2‐ and 4‐amino groups.[^6b^, ^6c^, ^6d^] In **3**, the conformation is forced into the flipped ^1^ *C*_4_ chair with all ring substituents in an axial orientation. The conformation flipping can be reversed by the addition of one equiv. of the strong zinc chelating ligand triethylenetetramine (TETA). In this reaction, zinc is precipitated as Zn‐TETA complex and the diamine **2** is obtained back in the ^4^ *C*_1_ conformation (Scheme 1A and Figure 1C). Furthermore, the ^1^ *C*_4_ conformation can be locked in the form of the cyclic urea derivative **4**, which was obtained in over 80 % yield when **2** was treated with 1,1’‐carbonyldiimidazol (CDI) and triethylamine in DMF (Scheme 1A and Figure 1D).

The NMR analysis of xylosides **1**–**4** provides information about the conformational characteristics of the 6‐membered ring. A marked difference between the ^1^H NMR spectra of **2** on the one hand and of **3** and **4** on the other hand is observed, indicating inverse chair conformations (Figure 1). The proton signals of the zinc complex **3** and of the cyclic urea derivative **4** are all downfield‐shifted in comparison to the spectrum of **2** and broadened. The small ^3^ *J*_H,H_ coupling constants (e.g., ^3^ *J*_1,2_<3.0 Hz) are expected for a d‐xylose ^1^ *C*_4_ conformer (Figure 1C and 1D and Table 1). Optimized geometries (B3LYP‐D4/def2‐TZVPP) of the conformations of **2** and **3** further illustrate the structural difference between the two conformers (Scheme 1B).

While the xylopyranosides **2**, **3**, and **4** occur as one single conformer, ^4^ *C*_1_ and ^1^ *C*_4_, respectively, as proven by the NMR analysis, the bis‐Boc‐protected xyloside **1** occurs as a dynamic mixture of conformers of unknown composition, which could not be resolved (cf. Figures S3–S13). The chair conformations of xylosides **2**, **3**, and **4** are stable either due to the intrinsic stability of the conformer, or by frustration of the internal degrees of freedom via linking the amino groups into bicyclic xylose derivatives (**3** and **4**). Hence, **2**, **3**, and **4** offer a basis to benchmark computational results against the measured NMR parameters. As simulated results always include approximations, our aim is to determine whether the agreement between experiment and theory is sufficient to unambiguously determine the most important conformers and to quantify conformational mixtures. For this purpose, xyloside **1** will provide the final test.

In the following, we will discuss computed energy differences, NMR chemical shifts and spin‐spin coupling constants and assess their deviation from experimental data and their sensitivity to distinguish different conformers. The aim of this approach is to find a property that can be computed efficiently and accurately so that conformers contributing to a given unknown conformational mixture such as in **1** can be described in detail by combining computed results with experimental benchmark data.

### Computed relative energies for conformers of 1 and 2

If the relative energies of a given set of conformers can be computed with high accuracy, their conformational equilibrium can be predicted using electronic structure methods. However, the inherent errors of electronic structure methods are not negligible. DFT does not necessarily yield sub‐kJ accuracy for energies.^[10]^ Moreover, zero‐point and temperature effects are typically treated in the harmonic approximation and solvent effects can be sizable.

In order to assess the structural features of xylosides **1** and **2**, their structures and relative energies were computed using a conformational search with the CREST^[11]^ protocol. Based on an ensemble of low energy conformers obtained from CREST, B3LYP‐D4 geometries and normal modes as well as DLPNO‐CCSD(T1)/cc‐pVTZ^[12]^ single point energies with inclusion of implicit solvent effects were evaluated. In analogy to the NMR experiments described above (Figure 1), compound **2** was computed in acetonitrile and for comparison also without solvent correction. Compound **1** on the other hand, was computed for the solvents DMSO (ϵ=47), acetonitrile (ϵ=38), acetone (ϵ=21), benzene (ϵ=2) and without solvent correction (cf. Tables S3 to S6).

**Figure 2:**
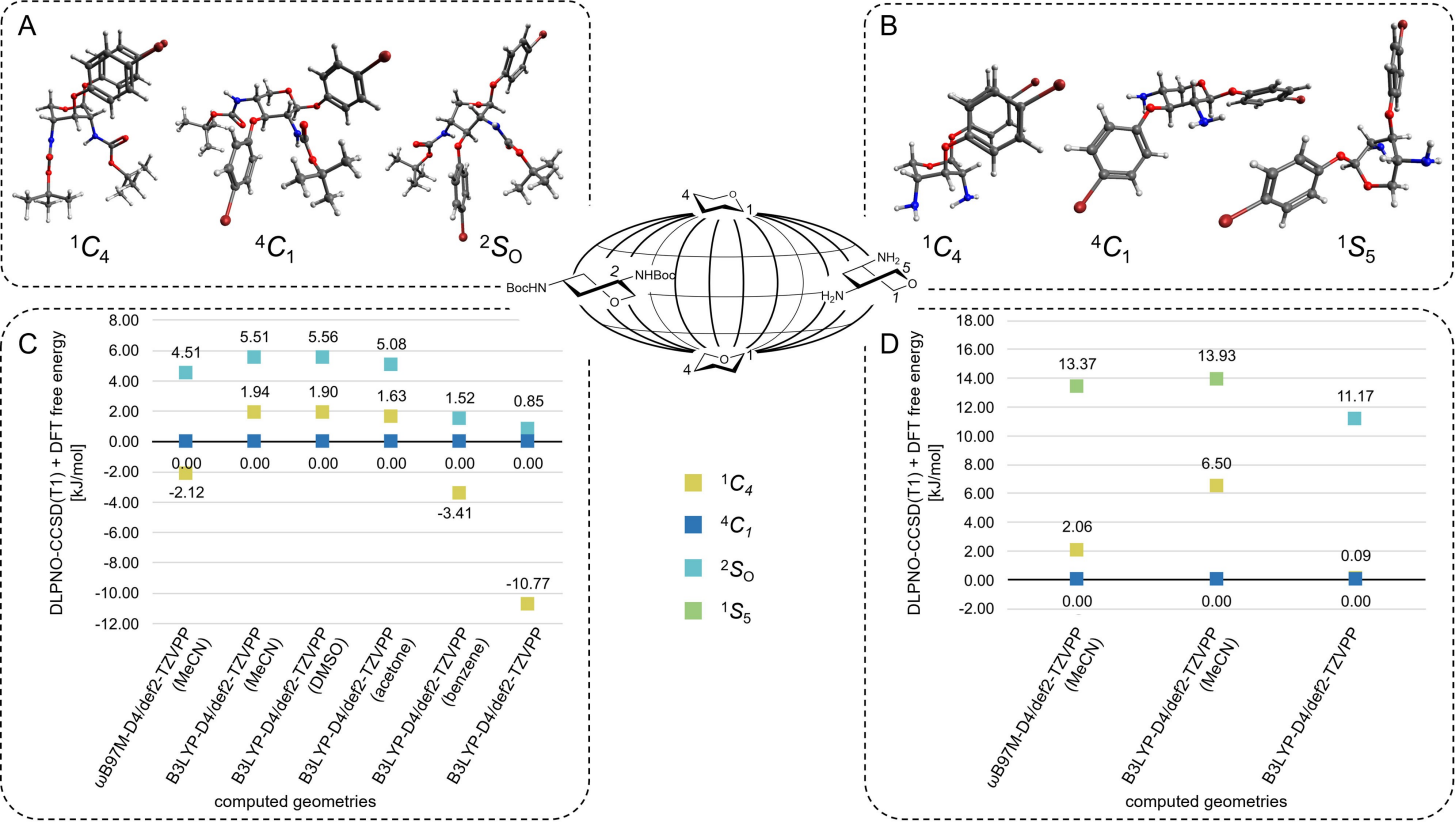
Cremer and Pople have used a spherical coordinate system to describe interconversions between specific sugar ring conformations.^[15]^ Here, the relevant pyranose puckering conformations of xylosides **1** and **2** are selected in the Cremer Pople landscape (center) and optimized geometries of the lowest energy conformers are shown ((A) for **1** and (B) for **2**). For computational details of the conformer search protocol see computational details. For all conformers, relative free energies (in kJ/mol) were obtained from conformational sampling (for **1** (C) and for **2** (D)). Implicit solvent (MeCN, using the conductor‐like polarizable continuum model CPCM)^[16]^ was taken into account, geometries, frequencies and enthalpy corrections were obtained at the B3LYP‐D4 and ωB97M‐D4 def2‐TZVPP level of theory. Single point energies were evaluated at the DLPNO‐CCSD(T1)/cc‐pVTZ^[12]^ level of theory. The lowest energy conformer was used as reference. Structures were rendered using Avogadro. Colour code: dark grey: carbon; red: oxygen; blue: nitrogen; white: hydrogen; dark red: bromine.

The results yield the ^1^ *C*_4_ and ^4^ *C*_1_ conformers as lowest energy structures for both xylosides **1** and **2**. Additional skew‐conformer minima were also found which could be identified as ^1^ *S*_5_ and ^2^ *S*_O_ (Figure 2A and B). All other conformers found in the CREST search are considerably higher in energy. Note that the conformers found and the range of energies observed are in agreement with what was found for other glycosides and related compounds.[^4^, ^13^]

For compound **2**, indeed the observed dominance of the ^4^ *C*_1_ conformer is found if solvent effects are taken into consideration (cf. Tables S1 to S2). Note that for compound **1** the energetics clearly show that with increasing dielectric constant of the solvent, the ^4^ *C*_1_ conformer is stabilized while in unpolar solvents, the ^1^ *C*_4_ conformer is the prevailing structure (cf. Tables S3 to S6). However, the results also show that the energetics alone are not sufficiently accurate to predict the conformational equilibrium of an unknown mixture in any detail. Using different methods yields results differing by several kJ/mol and a change in 1 kJ/mol easily leads to changes of more than 5 % in the Boltzmann distribution of two conformers at 350 K. The computed DLPNO‐CCSD(T1)/cc‐pVTZ energies using conformational sampling, DFT optimizations and free energy corrections with implicit solvation yield valuable information about which conformers contribute to a dynamic mixture, as also described by others.^[14]^ A more sensitive approach is required to quantify conformational equilibria.

### Measured and computed NMR chemical shifts

**Figure 3:**
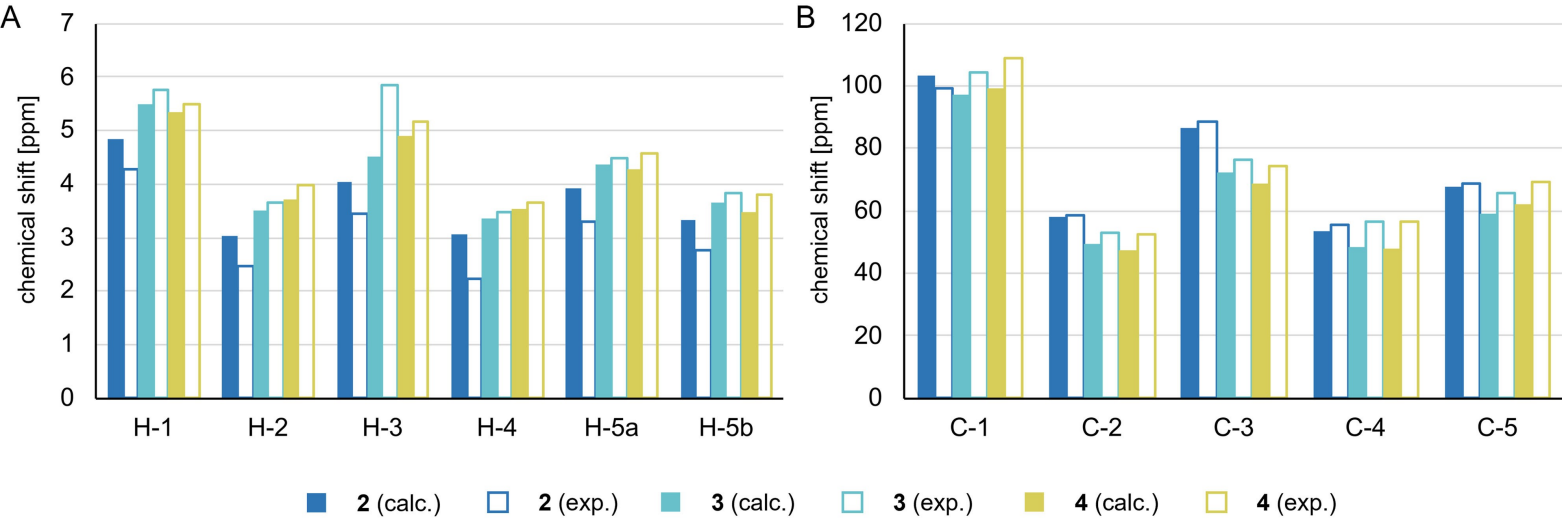
Comparison of calculated and measured chemical shifts (in ppm) for the pyranoside ring protons H‐1 to H‐5b (A) and carbons C‐1 to C‐5 (B) of compounds **2**, **3**, and **4** in MeCN‐d_3_. Proton signals (for numbering cf. Figure 1) were assigned by 2D NMR experiments (COSY and HSQC). Calculated values were obtained at the DLPNO‐MP2/pcSseg‐3^[19]^ level of theory with CPCM (MeCN).

NMR chemical shifts are typically very structure‐sensitive and can generally be computed with good accuracy.^[17]^ As structural changes between different conformers are mostly localized to the xyloside ring, Figure 3 summarizes the computed and measured ^1^H and ^13^C NMR chemical shifts of the ring protons H‐1 to H‐5b and of C‐1 to C‐5, respectively, for compounds **2**, **3** and **4** (the full set of chemical shifts is shown in Tables S7 and S8). For the ^1^H NMR chemical shifts, the agreement with the experiment is acceptable but not quantitative (RMSD of 0.49 ppm with MAD of 0.87 ppm). A closer analysis shows that the agreement for the xylose ring protons is noticeably better (RMSD of 0.36 ppm) than for the protons of the substituents (RMSD of 0.54 ppm). This is likely due to explicit interactions with the solvent and to the flexibility of substituents. The agreement of the computed ^13^C NMR chemical shifts is less good than for the protons (RMSD of 13.70 ppm with MAD of 30.37 ppm). Again, the agreement for carbons of the xylose ring (RMSD of 9.59 ppm) is better than for the substituents (RMSD of 15.87 ppm). Note that the computed carbon shifts for atoms in *ortho*‐ and *meta*‐position to the bromine atoms even exhibit the wrong trend, which is likely due to the absence of relativistic effects in our treatment.^[18]^

Overall, the agreement between experiment and theory is in the order of 0.3 to 0.5 ppm for ^1^H and 10 to 15 ppm for ^13^C chemical shifts. This has to be contrasted to the differences observed for the ^4^ *C*_1_ and ^1^ *C*_4_ conformers. The experimental results show that the ^1^H shieldings generally increase by roughly 0.5 ppm going from ^4^ *C*_1_ to ^1^ *C*_4_ while the ^13^C shifts show a decrease of 5–15 ppm for some of the xyloside carbon atoms (Figure 3, Tables S7 and S8). This means that the change of the experimental values from ^1^ *C*_4_ to ^4^ *C*_1_ conformers is of the same order of magnitude as the agreement of the computed and experimental numbers. In other words, deviations due to approximations in the electronic structure approach might be just as large as if simulating the wrong conformer. As a consequence, a compound of unknown conformation would be very hard to assign based on computed chemical shifts.

### Measured and computed spin‐spin coupling constants

In a next step, measured spin‐spin coupling constants were compared with computed data. The experimental coupling constants of the xylosides **2**, **3** and **4** were determined by NMR spectroscopy in different solvents (Table 1). Comparing the two compounds in ^1^ *C*_4_ conformation (**3** and **4**) with the ^4^ *C*_1_ conformer **2**, a strong trend can be observed. Especially the *J*_1,2_, *J*_2,3_, *J*_3,4_, and *J*_4,5b_ coupling constants are reduced by more than 6 Hz. Hence, the observed change in several spin‐spin coupling constants amounts to at least 3 Hz for the two main conformers. Furthermore, the couplings are very characteristic as expected from the well‐known Karplus correlation between coupling constants and ring conformation. In fact, Malkin and others have demonstrated that spin‐spin coupling constants of xylose can be computed with good accuracy using standard DFT methods.^[20]^ In the literature computed coupling constants have often been used to assess the ratios of conformers for example for mixtures of polysaccharides like heparin in aqueous solution. Here, DFT with implicit or explicit solvation has proven to be a valuable tool for analysis and rationalization of structure‐property relationships.[^14a^, ^14b^]

**Figure 4:**
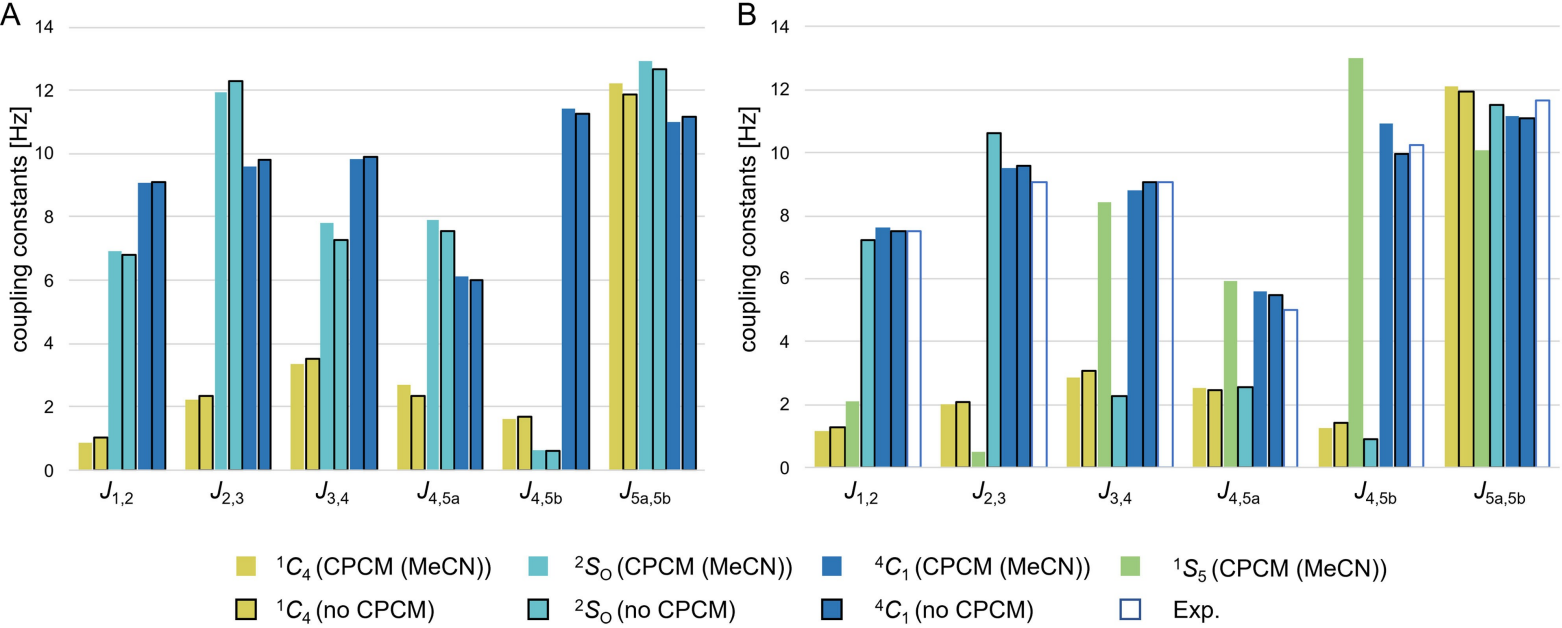
Computed spin‐spin coupling constants (PBE/pc‐J3) for the three lowest energy conformers obtained for compound **1** (A) and **2** (B). Coupling constants were calculated with CPCM (MeCN) and without implicit solvent. For **2**, the experimental coupling constants measured in MeCN‐d_3_ (B, white bars) are in excellent agreement with the computed coupling constants for the ^4^ *C*_1_ conformation (dark blue).

In Figure 4, the spin‐spin coupling constants for the six protons on the central xylose unit are depicted for the lowest energy conformers obtained at the B3LYP‐D4 and ωB97M‐D4 level of theory^[21]^ (geometries, with and without CPCM, couplings computed at the PBE/pc‐J3 level of theory; for further results cf. Tables S9–S12). For compound **2** (right panel), for which the experimental assignment is unambiguous, it is clear that irrespective of the level of theory, only for the ^4^ *C*_1_ conformer good agreement between computed couplings and the experimental values is obtained. All other conformers can be ruled out by a mismatch of more than 4 Hz for at least two coupling constants.

Overall, the comparison of the experimental and computed spin‐spin coupling constants (Table 1) shows that the deviation of the computed results at the PBE/pc‐J3^[22]^ level of theory is typically less than one Hz. The maximum absolute deviation (MAD) amounts to 0.5, 1.7 and 1.2 Hz for compound **2**, **3** and **4** without CPCM and 0.7, 1.6 and 1.2 Hz with CPCM, respectively. Hence, the choice of functional, geometry and even the inclusion of implicit solvation does not have a strong influence on the computed coupling constants.

From the computed couplings of **2** and **1** it is clearly visible that a distinct pattern in coupling constants distinguishes the two chair conformers and the skew conformer from each other. For both compounds, low spin‐spin coupling constant values for all couplings except for *J*_5a,5b_ are characteristic for the ^1^ *C*_4_ conformer, while for the ^4^ *C*_1_ conformer all coupling constants are at least 3 to 4 Hz larger, except for *J*_5a,5b_ which is 1 to 2 Hz smaller. The skew conformers found for compounds **1** and **2** exhibit a very different coupling pattern with alternating large and small coupling constants and very small coupling values for *J*_4,5b_ for compound **1** and *J*_2,3_ for compound **2**, respectively. This is in agreement with the experimentally obtained values measured for compounds **2**, **3**, and **4**.

If we contrast the observed strong changes in the coupling constants for the ^4^ *C*_1_ and ^1^ *C*_4_ conformers and the agreement between calculation and experiment, we find differences of several Hz between the different conformers, but only deviations of less than one Hz between theory and experiment. Hence, one can predict the xylose ring proton coupling constants of the pure conformers which are not accessible to experiment. This provides the missing link between observed coupling constants in the mixture and its composition. In the next section, we will focus on a proof‐of‐principle example for a combined computational and experimental quantification procedure for compound **1**.

### Solvent‐dependent conformational changes of xyloside 1

In the previous sections we have established that relative energies can be used to determine which are the most likely low energy conformers of pyranosides. While computed NMR chemical shifts do not allow a robust assignment of pyranoside conformation, the computation of the spin‐spin coupling constants of the xyloside ring protons can be used to predict the couplings of a given conformer with an accuracy that allows to reliably distinguish possible conformers. In the following, we discuss the computed spin‐spin coupling constants for the dynamic mixture of the conformers of xyloside **1**, for which the couplings of the pure conformers are not accessible separately in experiment. This serves as a proof‐of‐principle example for the combined experimental and computational scheme we exemplify here.

We saw that the coupling constants for the xylopyranoside **1** are solvent‐dependent. In fact, in several studies in the literature, a strong influence of solvation and intermolecular interactions on the conformation of related systems has been reported.[^14a^, ^14b^, ^24^] Thus, coupling constants of **1** were determined in ten deuterated solvents (Table 2). The solvents ranged from highly polar (DMSO‐d_6_ or DMF‐d_7_) containing hydrogen bond accepting and/or donating functionalities to non‐polar solvents (benzene‐d_6_ or CDCl_3_). In addition to the ^1^H NMR experiments, ^n^ *J*_HH_ coupling constants were measured in 2D experiments (COSY‐DQF).^[23]^ For the following calculations we assume as basic hypothesis that the different solutions of **1** constitute conformational equilibria in which various conformational isomers can quickly interconvert such that the observed NMR parameters are a Boltzmann average of the conformers in the mixtures. This is supported by several studies showing that conformational changes of the xylose ring are associated with low barriers and depending on the substitution pattern, several conformers can be low in energy within a narrow energy window of 5–10 kJ/mol.[^4^, ^14^, ^24^]

**Table 2:** Coupling constants of xyloside **1** determined in different solvents. For the experimental determination of the ^3^ *J*_H,H_ coupling constants (in Hz), ^1^H and COSY‐DQF NMR experiments were performed.^[23]^ Data in rows shaded in grey are calculated values and obtained at the PBE/pc‐J3 level of theory.

Solvent	*J*_1,2_	*J*_2,3_	*J*_3,4_	*J*_4,5a_	*J*_4,5b_	*J*_5a,5b_
DMSO‐d_6_^[a]^	8.03	9.66	9.66	5.15	11.37	11.37
DMF‐d_7_^[a]^	8.33	9.76	9.76	5.27	10.94	10.94
Acetone‐d_6_^[a]^	7.55	8.46	8.46	4.88	11.88	11.05
MeOD‐d_3_^[a]^	7.41	8.73	9.38	5.12	9.61	11.92
Pyridine‐d_5_^[a]^	6.77	8.34	8.34	4.69	9.89	11.51
THF‐d_8_^[a]^	5.87	7.89	7.89	4.49	9.18	11.84
Toluene‐d_8_^[b]^	4.38	6.10	6.10	3.87	6.58	11.83
Benzene‐d_6_^[b]^	n.d.	5.63	5.63	3.81	6.55	11.87
CDCl_3_^[b]^	4.17	5.53	5.53	3.70	5.97	12.04
Chlorobenzene‐d_5_^[b]^	4.21	5.42	5.42	3.47	5.50	12.00
B3LYP‐D4/B3LYP (^4^ *C*_1_)	10.17	10.93	10.87	6.56	12.39	12.66
B3LYP‐D4/B3LYP (^1^ *C*_4_)	1.26	2.53	3.76	2.68	2.00	13.35
B3LYP‐D4/B3LYP (^2^ *S*_O_)	7.48	13.35	8.04	8.27	0.83	14.20
B3LYP‐D4/PBE (^4^ *C*_1_)	9.09	9.79	9.92	6.03	11.27	11.15
B3LYP‐D4/PBE (^1^ *C*_4_)	1.04	2.35	3.50	2.37	1.70	11.89
B3LYP‐D4/PBE (^2^ *S*_O_)	6.82	12.29	7.27	7.54	0.60	12.65
B3LYP‐D4/PBE (MeCN) (^4^ *C*_1_)	9.07	9.58	9.85	6.15	11.40	11.02
B3LYP‐D4/PBE (MeCN) (^1^ *C*_4_)	0.87	2.21	3.34	2.70	1.60	12.22
B3LYP‐D4/PBE (MeCN) (^2^ *S*_O_)	6.93	11.93	7.82	7.89	0.65	12.91
ωB97M‐D4/PBE (MeCN) (^4^ *C*_1_)	9.14	9.34	9.98	6.29	11.45	10.98
ωB97M‐D4/PBE (MeCN) (^1^ *C*_4_)	1.27	2.40	3.25	2.83	1.56	12.35
ωB97M‐D4/PBE (MeCN) (^2^ *S*_O_)	6.80	11.96	7.36	7.47	0.47	12.95

[a] NMR experiments performed at 298 K. [b] NMR experiments performed at 328 K.

The NMR experiments showed that in polar solvents the coupling constants for the xylopyranoside ring protons are higher and that the ^4^ *C*_1_ conformation appears to be favoured. However, in less polar solvents with lower dielectric constant, the coupling constants of the conformational equilibria are decreasing, which is in line with the computed relative free energies discussed above.

From the experimental NMR results obtained for compound **1** in various solvents one can hypothesize that the changes in spin‐spin coupling constants are caused by intermolecular interactions of solvent molecules with the xyloside. This changes the energy difference between the two conformers and hence the conformer distribution, which would also shift the observed averaged coupling constants.

As can be seen from Table 2, the observed changes in the coupling constants for compound **1** depend very much on the polarity of the solvent and its ability to form hydrogen bonds. DMF and DMSO exhibit values expected for a ^4^ *C*_1_ conformer, while in chlorobenzene and especially CDCl_3_ all couplings are reduced, except for *J*_5a,5b_. This is exactly the trend for a transition to a ^1^ *C*_4_ conformer as predicted by the computed coupling constants for compound **1** and **2** (cf. Figure 4) and the experimentally determined coupling constants for compounds **3** and **4**. This is a strong argument for the assumption of a conformational equilibrium between the ^1^ *C*_4_ and ^4^ *C*_1_ conformers, which is determined by the interaction of the 2‐ and 4‐BocNH substituents of xyloside **1** with the solvent.

The experimental data depicted in Table 2 are the result of an average over a mixture of conformers with unknown composition. In order to disentangle the different contributions, it is necessary to know the spin‐spin couplings of the pure conformers. These are not accessible experimentally for compound **1**. However, we have shown above that spin‐spin couplings of the pure conformers can be computed with an accuracy of about one Hz. Detailed computational results using B3LYP‐D4 and ωB97M−D geometries using PBE0 and B3LYP with and without CPCM for the calculation of the *J*_1,2_, *J*_2,3_, *J*_3,4_, *J*_4,5a_, *J*_4,5b_ and *J*_5a,5b_ coupling constants of the ^1^ *C*_4_ and ^4^ *C*_1_ conformers of **1** can be found in Table 2. They show good consistency so that also for compound **1**, robust and reliable results can be expected at the DFT level of theory.

Based on the computational results for the two conformers of **1**, a least squares fit of the coupling constants observed in a given solvent to an average of the computed constants of the two pure conformers was performed to estimate the ^1^ *C*_4_:^4^ *C*_1_ distribution for every solvent. This fit was also carried out for a three‐component average including the lowest energy skew conformer obtained from the conformational search (^2^ *S*_0_, cf. Figure 2). However, the contribution of the skew conformer was never above 1 % and omitting if from the fit hardly affected the RMSD of the fitted values. Hence, we assume that the predominant conformers for compound **1** are ^1^ *C*_4_ and ^4^ *C*_1_.

Note that in principle, a rigorous inclusion of implicit solvation would require computing the lowest energy structures for every conformer from the ensemble generated by the CREST search separately for every solvent. However, our results indicate that the spin‐spin coupling constants are hardly affected by these influences while being highly sensitive to the conformation (cf. Figure 4). Hence, we emphasize on an assignment based on results without the inclusion of implicit solvation, which greatly simplifies the computation protocol. Note that in Table 3 we have also included the results of the least squares fit obtained based on geometries and couplings with CPCM (MeCN) as well as with other functionals, which all show very consistent results. The results compiled in Table 3 show that the fitted conformer ratios are fairly stable irrespective of the functional used with deviations of 1–2 % at most and RMSD values below one Hz.

**Table 3:** The ^1^ *C*_4_:^4^ *C*_1_ conformer distribution of xyloside **1** in various solvents is given as a result of a least squares fit of the type *J* ^i^_exp_(solv.)=a * *J* ^i^_calc_(^1^ *C*_4_)+b *J* ^i^_calc_(^4^ *C*_1_).

	B3LYP‐D4/PBE		B3LYP‐D4 (CPCM)/PBE (CPCM)		ωB97M‐D4 (CPCM)/PBE (CPCM)	
Solvent	^1^ *C*_4_:^4^ *C*_1_	RMSD	^1^ *C*_4_:^4^ *C*_1_	RMSD	^1^ *C*_4_:^4^ *C*_1_	RMSD
DMF‐d_7_	5 : 95	0.3	5 : 95	0.4	5 : 95	0.5
DMSO‐d_6_	5 : 95	0.5	5 : 95	0.5	5 : 95	0.6
Acetone‐d_6_	11 : 89	0.9	11 : 89	0.8	11 : 89	0.7
MeOD‐d_3_	17 : 83	0.4	16 : 84	0.5	17 : 83	0.5
Pyridine‐d_5_	22 : 78	0.4	21 : 79	0.5	22 : 78	0.6
THF‐d_8_	30 : 70	0.5	29 : 71	0.6	30 : 70	0.6
Toluene‐d_8_	54 : 46	0.3	53 : 47	0.4	54 : 46	0.4
Benzene‐d_6_	56 : 44	0.5	55 : 45	0.4	55 : 45	0.5
CDCl_3_	60 : 40	0.4	59 : 41	0.3	60 : 40	0.4
Chlorobenzene‐d_5_	62 : 38	0.3	61 : 39	0.3	62 : 38	0.4

**Figure 5:**
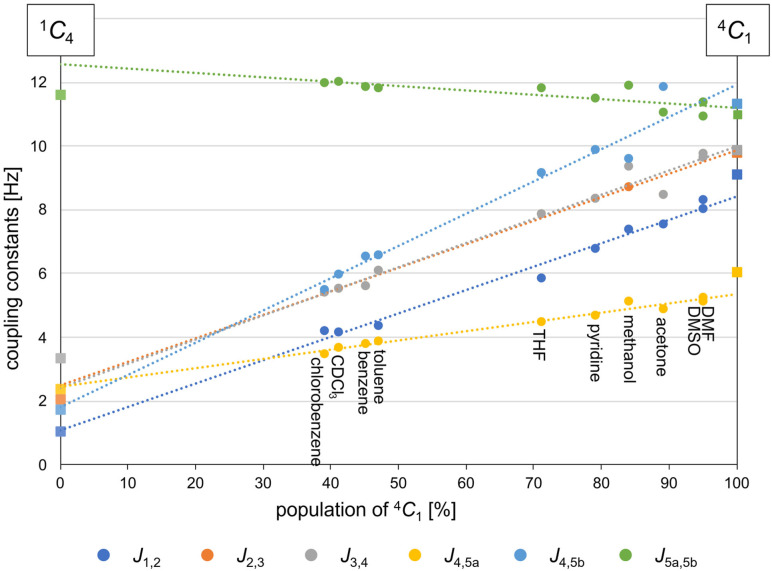
Dependence of experimental spin‐spin coupling constants on the fitted ratio between the ^4^ *C*_1_ and ^1^ *C*_4_ conformers of **1** in different solvents. For each of the couplings in each solvent, a least squares fit was carried out including *J*_1,2_, *J*_2,3_, *J*_3,4_ *J*_4,5a,_ *J*_4,5b,_ and *J*_5a,5b_ to obtain the optimal ^1^ *C*_4_:^4^ *C*_1_ ratio as plotted in % of ^4^ *C*_1_ on the x‐axis. Dotted lines illustrate a linear regression of the experimental values and computed ratios. Squares show the computed coupling constants for the ^1^ *C*_4_ conformer at 0 % and the ^4^ *C*_1_ conformer at 100 %, respectively.

The fitted ratios obtained without implicit solvent are assembled as x‐axis values in Figure 5, displaying how the couplings for compound **1** change depending on the percentage of the ^4^ *C*_1_ conformer. Note that depending on the solvent, the composition of the mixture changes from 5 : 95 in DMF up to 62 : 38 in chlorobenzene. At 293 K this amounts to a solvent‐induced shift in relative energy from +7 to −2 kJ/mol in favor of the ^4^ *C*_1_ conformer.

If these values are chosen for a linear regression for each coupling constant (Figure 5), it is possible to extrapolate the values for 0 and 100 % of ^4^ *C*_1_ and hence to estimate the coupling constants for the pure conformers from the experimental values. These are given in Table 4 (typical R^2^ values for the interpolation are in the order of 0.96–0.98 except for *J*_5a,5b_ for which the fit is, with R^2^=0.63, worse). The extrapolated coupling constants demonstrate that the combination of experimental and computational results enable us to obtain additional information like an estimate of the coupling constants for the pure conformers without having to rely solely on either experiment or theory.

**Table 4:** Extrapolated coupling constants for compound **1** using the experimental spin‐spin coupling constants obtained in various solvents and the ^1^ *C*_4_:^4^ *C*_1_ ratios obtained from a least squares fit of the computed values to the experimental ones. All values given in Hz; dev.: deviation.

	^1^ *C*_4_ extra‐ polated	^1^ *C*_4_ (PBE)	dev.	^4^ *C*_1_ extra‐ polated	^4^ *C*_1_ (PBE)	dev.
***J***_**1,2**_	1.23	1.04	0.19	8.43	9.09	−0.68
***J***_**2,3**_	2.63	2.35	0.28	9.89	9.79	0.10
***J***_**3,4**_	2.56	3.50	−0.94	10.02	9.92	0.10
***J***_**4,5a**_	2.52	2.37	0.15	5.36	6.03	−0.67
***J***_**4,5b**_	1.97	1.70	0.27	11.96	11.27	0.69
***J***_**5a,5b**_	12.55	11.89	0.66	11.20	11.15	0.05

## Conclusion

The conformational landscape of monosaccharides is complex. There are 38 canonical pyranose puckering conformations of monosaccharides which are frequently depicted in the form of a spherical coordinate system according to Cremer and Pople.^[16]^ Whereas often one of two chair conformations is the most populated one in a specific monosaccharide according to the energy difference between various possible other conformations, chemical modifications can lead to a conformational dynamics. This can be employed to control carbohydrate conformation and turn monosaccharides into molecular switches, which can be reversibly flipped between a ^4^ *C*_1_ and a ^1^ *C*_4_ conformation. This has recently been demonstrated for a di‐picolyl‐functionalized glucoside^[6a]^ and earlier, the conformation of 2,4‐diamino‐xylosides has been switched by complexation of the amino groups with Zn^2+^.[^6b^, ^6c^, ^6d^] Also in nature, monosaccharide conformation is altered by functionalization of the sugar ring to modulate biological function.^[5]^ In xylooligosaccharides, the sulfation pattern regulates the conformation of the sugar rings, modulating the biochemistry of these therapeutically relevant polysaccharides.^[25]^ Equally in the Notch receptor and in glycosaminoglycan (GAG) chains d‐xylose is an important constituent.^[26]^ Often mixtures of conformers are encountered in xylose‐containing oligosaccharides due to conformational equilibria with small energy differences and facile dynamics. Their experimental analysis, however, is difficult.

In our study we have investigated four xylosides, three of which can be obtained as pure conformers, whereas the xyloside **1** exists as a conformational equilibrium, which changes composition depending on the solvent. While this is a highly interesting observation, further analysis is demanding as the spectroscopic signatures of the pure conformers are unknown. Hence, there is too little information to decipher which conformers are present in the mixture and how large their fraction is.

A careful computational study in comparison to experiment shows that the spin‐spin coupling constants of the xylose moiety are not only very sensitive to the conformation, but can also be computed with sufficient accuracy using standard DFT methods. Consequently, it is possible to compute the coupling constants of the pure conformers and bridge the gap between the experimentally observed coupling constants and the conformer distribution. The composition of the conformer mixture is obtained by fitting the experimental values observed in different solvents.

An interesting finding is that while relative energies are very sensitive to solvent effects, their impact on the molecular geometry and the ^n^ *J*_H‐H_ coupling constants of the xylose moiety is almost negligible. This suggests that solvent effects mostly determine the relative energies of conformers and hence the ratio of different conformers but do not influence the coupling constants directly. Therefore, having obtained the ratios of the different conformers in the mixture, one can even extrapolate the hypothetical experimental coupling constants of the pure conformers to assess the agreement with the computed values.

Note that this procedure is fairly general and can potentially be applied to a broad range of carbohydrates and dynamic mixtures including several conformers. In fact, similar schemes have been applied for example to glycosaminoglycans by computing spin‐spin coupling constants for possible conformers and identifying the main contributions in conformational mixtures by comparison to experiment.^[14a]^ Apart from the composition, the fitting procedure also allows to estimate the experimental NMR parameters of the pure conformers as well as their relative energies. Hence, focussing on H−H spin‐spin coupling constants of monosaccharides, this combined experimental and computational scheme is suited to disentangle the complex NMR data of conformational mixtures, investigate changes in conformational equilibria and quantify solvent effects or intermolecular interactions for a broad range of xylosides and related carbohydrates. This scheme will assist the analysis and prediction of the conformational characteristics of monosaccharides as constituents of complex glycoconjugates and thus can be applied in the life sciences and in glycobiology, respectively, as well as for the development of carbohydrate foldamers and conformational switches.[^6a^, ^27^]

## Experimental Section

**Computational details**: All calculations were carried out with a local development version of the ORCA 5.0^[28]^ program package. All geometries were obtained from geometry optimizations at the B3LYP‐D4 and ωB97M‐D4/def2‐TZVPP level of theory (with and without CPCM) (RI approximation with def2 aux basis sets and tight optimization and convergence criteria).[^9^, ^21^] Spin‐spin coupling constants were computed at the DFT/pc‐J3^[22e]^ level of theory (RI with Autoaux basis set and tight convergence criteria). The functionals applied were PBE, TPSS, TPSSH, PBE0 and B3LYP.[^9a^, ^9b^, ^9c^, ^22^] Note that all contributions to the coupling constants (Fermi contact, paramagnetic and diamagnetic spin‐orbit as well as spin‐dipole) are included. NMR chemical shifts were obtained at the DLPNO‐MP2/pcSseg‐3^[19]^ level of theory with CPCM (MeCN) (no frozen core approximation, RIJCOSX approximation with def2/JK and cc‐pwCVQZ/C aux basis set and very tight convergence criteria) using gauge including atomic orbitals. Single point energies were obtained at the DLPNO‐CCSD(T1)/cc‐pVTZ^[12]^ level of theory (CPCM (MeCN),RI approximation with the corresponding JK and C basis sets).

In order to find low energy conformer structures for compounds **1** and **2**, several possible conformers were used as starting structures for the CREST protocol at the XTB level of theory.^[11]^ The resulting 20 lowest energy conformers and some additional non ^1^ *C*_4_ and ^4^ *C*_1_ structures were then further optimized at the B3LYP‐D4 and ωB97M‐D4/def2‐tzvpp (with and without CPCM) level of theory identifying the lowest energy conformers. Note that when we report results with CPCM herein, this refers to a complete optimization of the CREST ensemble of possible structures including CPCM and successive property calculations including CPCM. For results reported without CPCM the full workflow is carried out omitting CPCM.

**Dichloro [4‐Bromophenyl‐2,4‐N‐carbonyl‐2,4‐diamino‐2,4‐dideoxy‐3‐O‐(4‐bromophenyl)‐β‐d–xylopyranoside‐N,N’] zinc (3)**: The 2,4‐diamino xyloside **2** (2.00 mg, 4.37 μmol) was dissolved in acetonitrile‐d_3_ (600 μL) and zinc chloride (595 μg, 4.37 μmol) was added. The formation of the complex **3** was observed in a quantitative yield (according to ^1^H NMR spectroscopy). [α]^20^_D_=−70.62 (*c* 0.1, MeCN); IR (ATR): ν_max_/cm^−1^=2927, 1624, 1485, 1281, 1231, 1003, 825, 659; ^1^H NMR (500 MHz, MeCN‐d_3_, 328 K): δ=7.52 (d, ^3^ *J*=8.8 Hz, 2H, H‐6, H‐6’), 7.49 (d, ^3^ *J*=8.9 Hz, 2H, H‐8, H‐8’), 7.07 (d, ^3^ *J*=8.9 Hz, 2H, H‐9, H‐9’), 7.01 (d, ^3^ *J*=7.7 Hz, 2H, H‐7, H‐7’), 5.50 (d, ^3^ *J*_1,2_=2.18 Hz, 1H, H‐1), 4.52 (dd, ^3^ *J*_3,4_=3.90 Hz, ^3^ *J*_2,3_=3.54 Hz, 1H, H‐3), 4.36 (dd, ^3^ *J*_4,5a_=2.24 Hz, ^2^ *J*_5a,5b_=13.0 Hz, 1H, H‐5a), 3.65 (dd, ^3^ *J*_4,5b_=2.83 Hz, ^2^ *J*_5a,5b_=13.0 Hz, 1H, H‐5b), 3.51 (dd, ^3^ *J*_2,3_=3.54 Hz, ^3^ *J*_1,2_=2.18 Hz, 1H, H‐2), 3.37 (ddd, ^3^ *J*_3,4_=3.90 Hz, ^3^ *J*_4,5b_=2.83 Hz, ^3^ *J*_*4,5a*_=2.24 Hz, 1H, H‐4), ppm; ^13^C NMR (125 MHz, MeCN‐d_3_, 298 K): δ=156.45 (C‐12), 156.22 (C‐10), 133.53 (2 C, C‐6, C‐6’), 133.45 (2 C, C‐8, C‐8’), 119.82 (2 C, C‐9, C‐9’), 119.66 (2 C, C‐7, C‐7’), 115.05 (C‐11), 115.03 (C‐13), 97.00 (C‐1), 72.21 (C‐3), 59.07 (C‐5), 49.44 (C‐2), 48.34 (C‐4) ppm; HRMS (ESI): calcd. for [M−Cl]^+^ (C_17_H_18_Br_2_ClN_2_O_3_Zn): 554.8659; found *m/z*=554.8665.

**4‐Bromophenyl‐2,4‐*N*‐carbonyl‐2,4‐diamino‐2,4‐dideoxy‐3‐*O*‐(4‐bromophenyl)‐β‐d–xylopyranoside (4)**: The xyloside **2** (7.33 mg, 15.2 μmol) was dissolved in DMF (100 μL) and *N,N*‐carbonyldiimidazole (CDI, 3.00 mg, 18.2 μmol) was added followed by the dropwise addition of trimethylamine (8.50 μL, 60.8 μmol). The reaction mixture was stirred at RT for 16 h and was then diluted with ethyl acetate, washed with 1 n HCl, sodium bicarbonate and brine. The combined organic phases were dried over MgSO_4_, it was filtered and concentrated. Purification by column chromatography (CH_2_Cl_2_/MeOH, 95 : 5) gave **4** (6.00 mg, 12.3 μmol, 81 %) as a colourless solid. R_*f*_ 0.40 (CH_2_Cl_2_/MeOH, 95 : 5); [α]^20^_D_=−80.33 (*c* 0.1, CH_2_Cl_2_); IR (ATR): ν_max_/cm^−1^=3242, 2927, 2262, 1671, 1589, 1485, 1216, 1066, 1003, 821, 640; ^1^H NMR (600 MHz, MeCN‐d_3_, 298 K): δ=7.49 (d, ^3^ *J*=9.0 Hz, 2H, H‐6, H‐6’), 7.40 (d, ^3^ *J*=9.0 Hz, 2H, H‐8, H‐8’), 7.04 (d, ^3^ *J*=9.0 Hz, 2H, H‐9, H‐9’), 6.89 (d, ^3^ *J*=9.0 Hz, 2H, H‐7, H‐7’), 5.60 (s, 1H, NH), 5.50 (s, 1H, NH), 5.36 (d, ^3^ *J*_1,2_=2.80 Hz, 1H, H‐1), 4.89 (t, ^3^ *J*_3,4_=3.18 Hz, ^3^ *J*_2,3_=3.89 Hz, 1H, H‐3), 4.29 (dd, ^3^ *J*_4,5a_=1.67 Hz, ^2^ *J*_5a,5b_=12.0 Hz, 1H, H‐5a), 3.71 (dd, ^3^ *J*_2,3_=3.89 Hz, ^3^ *J*_1,2_=2.80 Hz, 1H, H‐2), 3.54 (ddd, ^3^ *J*_3,4_=3.18 Hz, ^3^ *J*_4,5b_=1.90 Hz, ^3^ *J*_*4,5a*_=1.67 Hz, 1H, H‐4), 3.49 (dd, ^3^ *J*_4,5b_=1.90 Hz, ^2^ *J*_5a,5b_=12.0 Hz, 1H, H‐5b) ppm; ^13^C NMR (125 MHz, MeCN‐d_3_, 298 K): δ=162.74 (C‐14), 157.08 (C‐12), 156.81 (C‐10), 133.47 (2 C, C‐6, C‐6’), 133.26 (2 C, C‐8, C‐8’), 119.45 (2 C, C‐9, C‐9’), 118.97 (2 C, C‐7, C‐7’), 114.71 (C‐11), 114.25 (C‐13), 99.13 (C‐1), 68.51 (C‐3), 62.01 (C‐5), 48.13 (C‐4), 47.18 (C‐2) ppm; HRMS (ESI): calcd. for [M+Na]^+^ (C_18_H_16_Br_2_N_2_NaO_4_): 504.9369; found *m/z*=504.9379.

## Conflict of interest

There are no conflicts of interest to declare.

1

## Supporting information

As a service to our authors and readers, this journal provides supporting information supplied by the authors. Such materials are peer reviewed and may be re‐organized for online delivery, but are not copy‐edited or typeset. Technical support issues arising from supporting information (other than missing files) should be addressed to the authors.

Supporting Information

## Data Availability

The data that support the findings of this study are available in the supplementary material of this article.
