# Supplementary material for: Between Two Chairs: Combination of Theory and Experiment for the Determination of the Conformational Dynamics of Xylosides
Source: Chemistry. 2022 Aug 4;28(56):e202201544. doi: 10.1002/chem.202201544 (PMC9804333; doi:10.1002/chem.202201544)
Supplement: Supplementary file 1 — Supporting Information [file CHEM-28-0-s001.pdf]

Chemistry–A European Journal

Supporting Information

Between Two Chairs: Combination of Theory and Experiment for the Determination of the Conformational Dynamics of Xylosides

Sven Ole Jaeschke, Thisbe K. Lindhorst,* and Alexander Auer*

Table of content

1 General methods for synthesis S2

2 Synthetic procedures S3

3 Computational details S9

4 NMR spectra of the synthesized compounds S24

5 References S40

1 General methods for synthesis

General experimental

Analytical thin layer chromatography (TLC) was performed on silica gel plates (GF 254, Merck). Visualization was achieved by UV light and/or with 10 % sulfuric acid in ethanol, vanillin (3.0 g vanillin and 0.5 mL H_2SO_4 in 100 mL EtOH) or ninhydrin, followed by heat treatment at approx. 200 °C. The products were purified by flash chromatography on silica gel columns (Merck, 230–400 mesh, particle size 0.040-0.063 mm) or by automated flash chromatography using a puriFlash 450 device from the Interchim® company. Optical rotations were measured with a PerkinElmer 241 polarimeter with a sodium D-line (589 nm) and a cuvette of 10 cm path length, in the solvents indicated. Proton (^{1}H) nuclear magnetic resonance spectra and carbon (^{13}C) nuclear magnetic resonance spectra were recorded on a Bruker DRX-500 or AV-600 instruments. Chemical shifts (δ) are given in ppm relative to the internal standard tetramethylsilane (TMS) or to the residual proton of the NMR solvent. Full assignment of the signals are supported by 2D NMR experiments (^{1}H–^{1}H COSY, ^{1}H–^{13}C HSQC, ^{1}H–^{1}H COSY-DQF). Data are presented as follows: chemical shift, (multiplicity: s for singlet, d for doublet, t = triplet, dd for doublet of doublets, m = multiplet; coupling constant [Hz], integration, assignment). Infrared (IR) spectra were measured with a PerkinElmer FT-IR Paragon 1000 (ATR) spectrometer and are reported in cm^{-1}. HR-ESI mass spectra were recorded on a LCQ Classic from Thermo Finnigan.

Nomenclature of ring conformations

The nomenclature of specific sugar ring conformations according to IUPAC is indicated by an italic capital letter, for example *C* for chair and *S* for skew-boat, with the atoms on the upper or lower side of the main ring plane in superscript and subscript lettering, respectively.[1]

2 Synthetic procedures

4-Bromophenyl 2,4-dideoxy-2,4-*N*-Boc-3-*O*-(4-bromophenyl)-β-D-xylopyranoside (1)

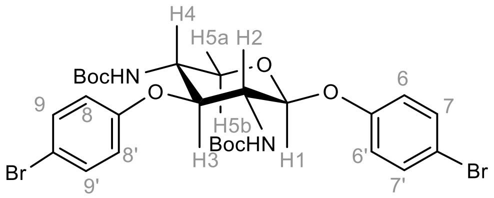

The xyloside **1** was synthesized according to the procedure published by Jaeschke and Lindhorst.[2]

$[\alpha]^{20}_{D}$ = -28.54 (*c* 0.1, acetone); IR (ATR): ν_{max}/cm^{-1} = 3325, 2977, 1681, 1538, 1487, 1303, 1228, 1172, 1031, 818; ^{1}H NMR (500 MHz, DMF-d_7, 300 K): δ = 7.52 (d, ^3J = 8.9 Hz, 2H, H-6, H-6’), 7.43 (d, ^3J = 9.0 Hz, 2H, H-8, H-8’), 7.21–6.97 (m, 6H, NH, NH, H-9, H-9’, H-7, H-7’), 5.27 (d, $^3J_{1,2}$ = 8.33 Hz, 1H, H-1), 4.76 (t, $^3J_{3,4}$ = 9.76 Hz, $^3J_{2,3}$ = 9.76 Hz, 1H, H-3), 3.95 (dd, $^2J_{5a,5b}$ = 10.94 Hz, $^3J_{5a,4}$ = 5.27 Hz, 1H, H-5a), 3.88 (m, 1H, H-4), 3.82 (m, 1H, H-2), 3.66 (m, 2H, H-2, H-5b), 1.31 (s, 9H, $C(CH_3)_3$), 1.28 (s, 9H, $C(CH_3)_3$) ppm; (500 MHz, DMSO-d_6, 300 K): δ = 7.49 (d, ^3J = 8.8 Hz, 2H, H-6, H-6’), 7.41 (d, ^3J = 8.8 Hz, 2H, H-8, H-8’), 7.09 (d, $^3J_{NH,2}$ = 9.3 Hz, 1H, NH), 7.03 (d, $^3J_{NH,4}$ = 9.2 Hz, 1H, NH), 6.97 (d, ^3J = 8.8 Hz, 2H, H-9, H-9’), 6.93 (d, ^3J = 8.8 Hz, 2H, H-7, H-7’), 5.04 (d, $^3J_{1,2}$ = 8.03 Hz, 1H, H-1), 4.48 (t, $^3J_{3,4}$ = 9.66 Hz, $^3J_{2,3}$ = 9.66 Hz, 1H, H-3), 3.80 (dd, $^2J_{5a,5b}$ = 11.37 Hz, $^3J_{5a,4}$ = 5.15 Hz, 1H, H-5a), 3.68 (m, 1H, H-4), 3.60 (m, 1H, H-2), 3.43 (dd, $^2J_{5a,5b}$ = 11.37 Hz, $^3J_{5b,4}$ = 11.37 Hz, 1H, H-5b), 1.27 (s, 9H, $C(CH_3)_3$), 1.24 (s, 9H, $C(CH_3)_3$) ppm; (500 MHz, acetone-d_6, 300 K): δ = 7.47 (d, ^3J = 8.9 Hz, 2H, H-6, H-6’), 7.38 (d, ^3J = 8.9 Hz, 2H, H-8, H-8’), 7.07–6.98 (m, 4H, H-9, H-9’, H-7, H-7’), 6.48 (d, $^3J_{NH,2}$ = 9.0 Hz, 1H, NH), 6.32 (d, $^3J_{NH,4}$ = 8.0 Hz, 1H, NH), 5.35 (d, $^3J_{1,2}$ = 7.7 Hz, 1H, H-1), 4.88 (t, $^3J_{3,4}$ = 9.1 Hz, $^3J_{2,3}$ = 9.1 Hz, 1H, H-3), 3.99 (dd, $^2J_{5a,5b}$ = 11.2 Hz, $^3J_{5a,4}$ = 4.7 Hz, 1H, H-5a), 3.88 (m, 1H, H-4), 3.91–3.84 (m, 1H, H-2), 3.80–3.65 (m, 1H, H-5b), 1.32 (s, 9H, $C(CH_3)_3$), 1.30 (s, 9H, $C(CH_3)_3$) ppm; (500 MHz, MeOD-d_3, 300 K): δ = 7.41 (d, ^3J = 8.9 Hz, 2H, H-6, H-6’), 7.35 (d, ^3J = 9.0 Hz, 2H, H-8, H-8’), 6.99 (d, ^3J = 9.0 Hz, 2H, H-9, H-9’), 6.94 (d, ^3J = 8.8 Hz, 2H, H-7, H-7’), 5.14 (d, $^3J_{1,2}$ = 7.41 Hz, 1H, H-1), 4.59 (t, $^3J_{3,4}$ = 9.38 Hz, $^3J_{2,3}$ = 8.73 Hz, 1H, H-3), 3.97 (dd, $^2J_{5a,5b}$ = 11.92 Hz, $^3J_{5a,4}$ = 5.12 Hz, 1H, H-5a), 3.80 (ddd, $^3J_{3,4}$ = 9.38 Hz, $^3J_{4,5b}$ = 9.61 Hz, $^3J_{4,5a}$ = 5.12 Hz, 1H, H-4), 3.69 (dd, $^3J_{2,3}$ = 8.73 Hz, $^3J_{1,2}$ = 7.41 Hz, 1H, H-2), 3.52

(dd, $^3J_{4,5b}$ = 9.61 Hz, $^2J_{5a,5b}$ = 11.92 Hz, 1H, H-5b), 1.34 (s, 9H, $C(CH_3)_3$), 1.32 (s, 9H, $C(CH_3)_3$) ppm; (500 MHz, Pyridine-d_5, 328 K): δ = 7.35 (d, ^3J = 8.9 Hz, 2H, H-6, H-6'), 7.30 (d, ^3J = 8.9 Hz, 2H, H-8, H-8'), 7.15 (d, ^3J = 8.8 Hz, 2H, H-9, H-9'), 7.05 (d, ^3J = 8.8 Hz, 2H, H-7, H-7'), 5.78 (d, $^3J_{1,2}$ = 6.77 Hz, 1H, H-1), 5.32 (t, $^3J_{3,4}$ = 7.89 Hz, $^3J_{2,3}$ = 7.89 Hz, 1H, H-3), 4.27 (dd, $^2J_{5a,5b}$ = 11.51 Hz, $^3J_{5a,4}$ = 4.69 Hz, 1H, H-5a), 4.22 (ddd, $^3J_{3,4}$ = 8.34 Hz, $^3J_{4,5b}$ = 9.89 Hz, $^3J_{4,5a}$ = 4.69 Hz, 1H, H-4), 4.11 (dd, $^3J_{2,3}$ = 8.34 Hz, $^3J_{1,2}$ = 6.77 Hz, 1H, H-2), 3.88 (dd, $^3J_{4,5b}$ = 9.89 Hz, $^2J_{5a,5b}$ = 11.51 Hz, 1H, H-5b), 1.37 (s, 9H, $C(CH_3)_3$), 1.34 (s, 9H, $C(CH_3)_3$) ppm; (500 MHz, THF-d_8, 300 K): δ = 7.38 (d, ^3J = 8.7 Hz, 2H, H-6, H-6'), 7.33 (d, ^3J = 8.3 Hz, 2H, H-8, H-8'), 7.03 (d, ^3J = 8.0 Hz, 2H, H-9, H-9'), 6.95 (d, ^3J = 8.5 Hz, 2H, H-7, H-7'), 6.66 (d, $^3J_{NH,2}$ = 7.6 Hz, 1H, NH), 6.40 (d, $^3J_{NH,4}$ = 7.2 Hz, 1H, NH), 5.39 (d, $^3J_{1,2}$ = 5.87 Hz, 1H, H-1), 4.82 (t, $^3J_{3,4}$ = 7.89 Hz, $^3J_{2,3}$ = 7.89 Hz, 1H, H-3), 4.01 (dd, $^2J_{5a,5b}$ = 11.84 Hz, $^3J_{5a,4}$ = 4.49 Hz, 1H, H-5a), 3.75 (ddd, $^3J_{3,4}$ = 7.89 Hz, $^3J_{4,5b}$ = 9.18 Hz, $^3J_{4,5a}$ = 4.49 Hz, 1H, H-4), 3.55 (dd, $^3J_{2,3}$ = 7.89 Hz, $^3J_{1,2}$ = 5.87 Hz, 1H, H-2), 3.52 (dd, $^3J_{4,5b}$ = 9.18 Hz, $^2J_{5a,5b}$ = 11.84 Hz, 1H, H-5b), 1.35 (s, 9H, $C(CH_3)_3$), 1.32 (s, 9H, $C(CH_3)_3$) ppm; (500 MHz, toluene-d_8, 328 K): δ = 7.19 (d, ^3J = 8.9 Hz, 2H, H-6, H-6'), 7.13 (d, ^3J = 8.9 Hz, 2H, H-8, H-8'), 6.89 (d, ^3J = 8.9 Hz, 2H, H-9, H-9'), 6.71 (d, ^3J = 8.9 Hz, 2H, H-7, H-7'), 5.10 (d, $^3J_{1,2}$ = 4.38 Hz, 1H, H-1), 4.76 (t, $^3J_{3,4}$ = 6.10 Hz, $^3J_{2,3}$ = 6.10 Hz, 1H, H-3), 4.42 (d, $^3J_{NH,2}$ = 7.9 Hz, 1H, NH), 4.18 (d, $^3J_{NH,4}$ = 3.5 Hz, 1H, NH), 3.89 (dd, $^2J_{5a,5b}$ = 11.83 Hz, $^3J_{5a,4}$ = 3.87 Hz, 1H, H-5a), 3.55 (ddd, $^3J_{3,4}$ = 6.10 Hz, $^3J_{4,5b}$ = 6.58 Hz, $^3J_{4,5a}$ = 3.87 Hz, 1H, H-4), 3.49 (dd, $^3J_{2,3}$ = 6.10 Hz, $^3J_{1,2}$ = 4.38 Hz, 1H, H-2), 3.09 (dd, $^3J_{4,5b}$ = 6.58 Hz, $^2J_{5a,5b}$ = 11.83 Hz, 1H, H-5b), 1.36 (s, 9H, $C(CH_3)_3$), 1.32 (s, 9H, $C(CH_3)_3$) ppm; (500 MHz, benzene-d_6, 328 K): δ = 7.24 (d, ^3J = 8.8 Hz, 2H, H-6, H-6'), 7.18 (d, ^3J = 8.8 Hz, 2H, H-8, H-8'), 6.94 (d, ^3J = 8.8 Hz, 2H, H-9, H-9'), 6.75 (d, ^3J = 8.8 Hz, 2H, H-7, H-7'), 5.18 (s, 1H, H-1), 4.85 (t, $^3J_{3,4}$ = 5.63 Hz, $^3J_{2,3}$ = 5.63 Hz, 1H, H-3), 4.50 (d, $^3J_{NH,2}$ = 7.9 Hz, 1H, NH), 4.22 (s, 1H, NH), 3.92 (dd, $^2J_{5a,5b}$ = 11.87 Hz, $^3J_{5a,4}$ = 3.81 Hz, 1H, H-5a), 3.65 (ddd, $^3J_{3,4}$ = 5.63 Hz, $^3J_{4,5b}$ = 6.55 Hz, $^3J_{4,5a}$ = 3.81 Hz, 1H, H-4), 3.56 (dd, $^3J_{2,3}$ = 5.63 Hz, 1H, H-2), 3.11 (dd, $^3J_{4,5b}$ = 6.55 Hz, $^2J_{5a,5b}$ = 11.87 Hz, 1H, H-5b), 1.38 (s, 9H, $C(CH_3)_3$), 1.33 (s, 9H, $C(CH_3)_3$) ppm; (500 MHz, $CDCl_3$, 328 K): δ = 7.40-7.37 (m, 4H, H-6, H-6', H-8, H-8'), 6.99 (d, ^3J = 8.9 Hz, 2H, H-9, H-9'), 6.91 (d, ^3J = 9.0 Hz, 2H, H-7, H-7'), 5.40 (d, $^3J_{1,2}$ = 4.17 Hz, 1H, H-1), 4.95 (d, $^3J_{NH,2}$ = 8.1 Hz, 1H, NH), 4.83 (t, $^3J_{3,4}$ = 5.53 Hz, $^3J_{2,3}$ = 5.53 Hz, 1H, H-3), 4.72 (d, $^3J_{NH,4}$ = 4.6 Hz, 1H, NH), 4.28 (dd, $^2J_{5a,5b}$ = 12.04 Hz, $^3J_{5a,4}$ = 3.70 Hz, 1H, H-5a), 3.79 (ddd, $^3J_{3,4}$ = 5.53 Hz, $^3J_{4,5b}$ = 5.97 Hz, $^3J_{4,5a}$ = 3.70 Hz, 1H, H-4), 3.75 (dd, $^3J_{2,3}$ = 5.53 Hz, 1H, H-2), 3.55 (dd, $^3J_{4,5b}$ = 5.97 Hz, $^2J_{5a,5b}$ = 12.04

Hz, 1H, H-5b), 1.43 (s, 9H, $C(CH_3)_3$), 1.40 (s, 9H, $C(CH_3)_3$) ppm; (500 MHz, chlorobenzene-d$_5$, 328 K): δ = 7.00 (d, ^3J = 8.8 Hz, 2H, H-6, H-6’), 6.98 (d, ^3J = 8.9 Hz, 2H, H-8, H-8’), 6.74 (d, ^3J = 8.8 Hz, 2H, H-9, H-9’), 6.56 (d, ^3J = 8.8 Hz, 2H, H-7, H-7’), 5.04 (d, $^3J_{1,2}$ = 4.21 Hz, 1H, H-1), 4.67 (t, $^3J_{3,4}$ = 5.42 Hz, $^3J_{2,3}$ = 5.42 Hz, 1H, H-3), 4.60 (d, $^3J_{NH,2}$ = 7.3 Hz, 1H, NH), 4.38 (d, $^3J_{NH,4}$ = 5.9 Hz, 1H, NH), 3.90 (dd, $^2J_{5a,5b}$ = 12.00 Hz, $^3J_{5a,4}$ = 3.47 Hz, 1H, H-5a), 3.58 (ddd, $^3J_{3,4}$ = 5.42 Hz, $^3J_{4,5b}$ = 5.50 Hz, $^3J_{4,5a}$ = 3.47 Hz, 1H, H-4), 3.45 (dd, $^3J_{2,3}$ = 5.42 Hz, 1H, H-2), 3.08 (dd, $^3J_{4,5b}$ = 5.50 Hz, $^2J_{5a,5b}$ = 12.00 Hz, 1H, H-5b), 1.19 (s, 9H, $C(CH_3)_3$), 1.15 (s, 9H, $C(CH_3)_3$) ppm; ^{13}C NMR (125 MHz, acetone-d$_6$, 298 K): δ = 160.13 (C-12), 157.84 (C-10), 156.20 (NH-<u>C</u>(O)), 156.12 (C4-NH-<u>C</u>(O)), 133.21 (2C, C-6, C-6’), 132.80 (2C, C-8, C-8’), 119.83 (2C, C-9, C-9’), 119.81 (2C, C-7, C-7’), 115.17 C-11), 113.73 (C-13), 100.41 (C-1), 79.25 (2 <u>C</u>(CH_3)$_3$), 79.20 (C-3), 64.58 (C-5), 57.71 (C-2), 53.08 (C-4), 28.50 (6 C(<u>C</u>H$_3$)$_3$) ppm; HRMS (ESI): calcd. for $[M+Na]^+$ ($C_{27}H_{34}Br_2N_2NaO_7$): 679.0625; found *m/z* = 679.0629.

4-Bromophenyl 2,4-diamino-2,4-dideoxy-3-*O*-(4-bromophenyl)-β-D-xylopyranoside (2)

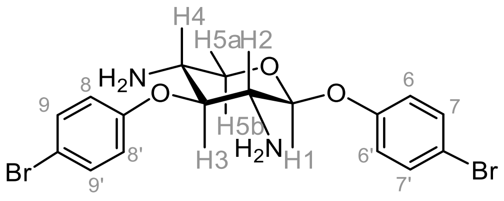

The xyloside **2** was synthesized according to the procedure published by Jaeschke and Lindhorst.[2]

$[\alpha]^{20}_D$ = -10.57 (*c* 0.08, CH_2Cl_2); IR (ATR): ν_{max}/cm^{-1} = 3304, 2922, 1578, 1484, 1226, 1068, 1004, 820, 659; ^{1}H NMR (600 MHz, acetone-d$_6$, 298 K): δ = 7.44 (d, ^3J = 9.0 Hz, 2H, H-6, H-6’), 7.33 (d, ^3J = 9.1 Hz, 2H, H-8, H-8’), 6.99 (d, ^3J = 9.0 Hz, 2H, H-9, H-9’), 6.93 (d, ^3J = 9.0 Hz, 2H, H-7, H-7’), 5.28 (d, $^3J_{1,2}$ = 7.6 Hz, 1H, H-1), 4.58 (t, $^3J_{3,4}$ = 9.2 Hz, $^3J_{2,3}$ = 9.2 Hz, 1H, H-3), 4.00–3.94 (m, 1H, H-4), 3.91 (dd, $^3J_{2,3}$ = 9.3 Hz, $^3J_{1,2}$ = 7.6 Hz, 1H, H-2), 3.81–3.76 (m, 2H, H-5a, H-5b); (600 MHz, $CDCl_3$, 298 K, TMS): δ = 7.40 (d, ^3J = 8.9 Hz, 2H, H-6, H-6’), 7.39 (d, ^3J = 9.0 Hz, 2H, H-8, H-8’), 7.07 (d, ^3J = 9.0 Hz, 2H, H-9, H-9’), 7.01 (d, ^3J = 8.9 Hz, 2H, H-7, H-7’), 4.84 (d, $^3J_{1,2}$ = 7.60 Hz, 1H, H-1), 4.05 (m, 1H, H-3), 4.03 (m, 1H, H-5a), 3.32 (1H, H-5b), 3.28 (1H, H-4), 3.26 (m, 1H, H-2) ppm; (600 MHz, MeCN-d$_3$, 298 K): δ = 7.45 (d, ^3J = 8.9 Hz, 2H, H-6, H-6’), 7.42

(d, ^3J = 9.0 Hz, 2H, H-8, H-8'), 7.08 (d, ^3J = 9.0 Hz, 2H, H-9, H-9'), 6.99 (d, ^3J = 8.9 Hz, 2H, H-7, H-7'), 4.84 (d, $^3J_{1,2}$ = 7.52 Hz, 1H, H-1), 4.03 (t, $^3J_{3,4}$ = 9.06 Hz, $^3J_{2,3}$ = 9.06 Hz, 1H, H-3), 3.91 (dd, $^3J_{4,5a}$ = 5.03 Hz, $^2J_{5a,5b}$ = 11.66 Hz, 1H, H-5a), 3.34 (dd, $^3J_{4,5b}$ = 10.22 Hz, $^2J_{5a,5b}$ = 11.66 Hz, 1H, H-5b), 3.05 (ddd, $^3J_{3,4}$ = 9.06 Hz, $^3J_{4,5b}$ = 10.22 Hz, $^3J_{4,5a}$ = 5.03 Hz, 1H, H-4), 3.02 (dd, $^3J_{2,3}$ = 9.06 Hz, $^3J_{1,2}$ = 7.52 Hz, 1H, H-2) ppm; (500 MHz, DMSO-d$_6$, 298 K): δ = 7.48 (d, ^3J = 8.9 Hz, 2H, H-6, H-6'), 7.42 (d, ^3J = 9.0 Hz, 2H, H-8, H-8'), 7.14 (d, ^3J = 9.0 Hz, 2H, H-9, H-9'), 7.01 (d, ^3J = 9.0 Hz, 2H, H-7, H-7'), 4.90 (d, $^3J_{1,2}$ = 7.44 Hz, 1H, H-1), 4.07 (t, $^3J_{3,4}$ = 8.93 Hz, $^3J_{2,3}$ = 8.93 Hz, 1H, H-3), 3.84 (dd, $^3J_{4,5a}$ = 5.00 Hz, $^2J_{5a,5b}$ = 11.54 Hz, 1H, H-5a), 3.32 (dd, $^3J_{4,5b}$ = 10.73 Hz, $^2J_{5a,5b}$ = 11.54 Hz, 1H, H-5b), 2.92 (ddd, $^3J_{3,4}$ = 8.93 Hz, $^3J_{4,5b}$ = 10.73 Hz, $^3J_{4,5a}$ = 5.00 Hz, 1H, H-4), 2.88 (dd, $^3J_{2,3}$ = 8.93 Hz, $^3J_{1,2}$ = 7.44 Hz, 1H, H-2), 1.67 (s, 4H, NH_2) ppm; ^{13}C NMR (125 MHz, MeCN-d$_3$, 298 K): δ = 160.90 (C-12), 157.71 (C-10), 133.31 (2C, C-6, C-6'), 133.15 (2C, C-8, C-8'), 119.68 (2C, C-9, C-9'), 119.56 (2C, C-7, C-7'), 115.13 (C-11), 113.68 (C-13), 103.68 (C-1), 86.34 (C-3), 67.76 (C-5), 57.99 (C-2), 53.48 (C-4), ppm; (125 MHz, $CDCl_3$, 298 K): δ = 159.19 (C-12), 156.23 (C-10), 132.82 (2C, C-6, C-6'), 132.60 (2C, C-8, C-8'), 118.86 (2C, C-9, C-9'), 118.33 (2C, C-7, C-7'), 115.52 (C-11), 114.32 (C-13), 102.84 (C-1), 85.81 (C-3), 67.35 (C-5), 56.79 (C-2), 52.46 (C-4), ppm; HRMS (ESI): calcd. for $[M+Na]^+$ ($C_{17}H_{18}Br_2N_2NaO_3$): 456.9757; found *m/z* = 456.9761.

Dichloro [4-Bromophenyl 2,4-diamino-2,4-dideoxy-3-*O*-(4-bromophenyl)-β-D-xylopyranoside-*N*,*N*'] zinc (3)

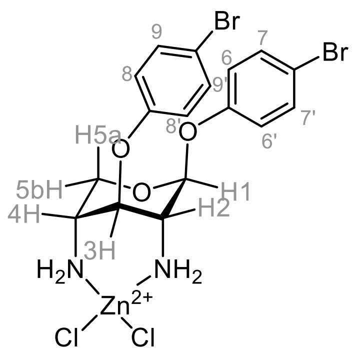

The 2,4-diamino xyloside **2** (2.00 mg, 4.37 µmol) was dissolved in MeCN-d$_3$ (600 µL) and zinc chloride (595 µg, 4.37 µmol) was added. Formation of the complex **3** was observed in a quantitative yield (according to ^{1}H NMR analysis). $[\alpha]^{20}_D$ = -70.62 (*c* 0.1, MeCN); IR (ATR): ν_{max}/cm^{-1} = 2927, 1624, 1485, 1281, 1231, 1003, 825, 659; ^{1}H NMR

(500 MHz, MeCN-d$_3$, 328 K): δ = 7.52 (d, ^3J = 8.8 Hz, 2H, H-6, H-6'), 7.49 (d, ^3J = 8.9 Hz, 2H, H-8, H-8'), 7.07 (d, ^3J = 8.9 Hz, 2H, H-9, H-9'), 7.01 (d, ^3J = 7.7 Hz, 2H, H-7, H-7'), 5.50 (d, $^3J_{1,2}$ = 2.18 Hz, 1H, H-1), 4.52 (dd, $^3J_{3,4}$ = 3.90 Hz, $^3J_{2,3}$ = 3.54 Hz, 1H, H-3), 4.36 (dd, $^3J_{4,5a}$ = 2.24 Hz, $^2J_{5a,5b}$ = 13.0 Hz, 1H, H-5a), 3.65 (dd, $^3J_{4,5b}$ = 2.83 Hz, $^2J_{5a,5b}$ = 13.0 Hz, 1H, H-5b), 3.51 (dd, $^3J_{2,3}$ = 3.54 Hz, $^3J_{1,2}$ = 2.18 Hz, 1H, H-2), 3.37 (ddd, $^3J_{3,4}$ = 3.90 Hz, $^3J_{4,5b}$ = 2.83 Hz, $^3J_{4,5a}$ = 2.24 Hz, 1H, H-4), ppm; ^{13}C NMR (125 MHz, MeCN-d$_3$, 298 K): δ = 156.45 (C-12), 156.22 (C-10), 133.53 (2C, C-6, C-6'), 133.45 (2C, C-8, C-8'), 119.82 (2C, C-9, C-9'), 119.66 (2C, C-7, C-7'), 115.05 (C-11), 115.03 (C-13), 97.00 (C-1), 72.21 (C-3), 59.07 (C-5), 49.44 (C-2), 48.34 (C-4) ppm; HRMS (ESI): calcd. for [M-Cl]$^+$ ($C_{17}H_{18}Br_2ClN_2O_3Zn$): 554.8659; found *m/z* = 554.8665.

4-Bromophenyl 2,4-*N*-carbonyl-2,4-diamino-2,4-dideoxy-3-*O*-(4-bromophenyl)-β-D-xylopyranoside (4)

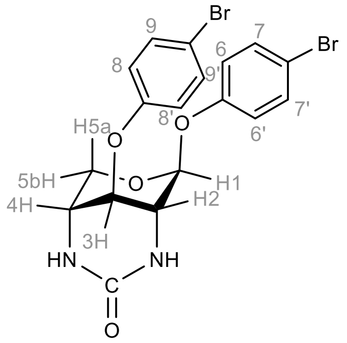

The xyloside **2** (7.33 mg, 15.2 µmol) was dissolved in DMF (100 µL) and *N,N*-carbonyldiimidazole (CDI, 3.00 mg, 18.2 µmol) was added. Subsequently, trimethylamine (8.50 µL, 60.8 µmol) was added. The reaction mixture was stirred for 16 h at RT and was then diluted with ethyl acetate, washed with 1 N HCl, sodium bicarbonate and brine. The combined organic phases were dried over $MgSO_4$, it was filtered and concentrated. Purification by column chromatography (CH_2Cl_2/MeOH, 95:5) gave **4** (6.00 mg, 12.3 µmol, 81 %) as a colourless solid. R_f 0.40 (CH_2Cl_2/MeOH, 95:5); $[\alpha]^{20}_D$ = -80.33 (*c* 0.1, CH_2Cl_2); IR (ATR): ν_{max}/cm^{-1} = 3242, 2927, 2262, 1671, 1589, 1485, 1216, 1066, 1003, 821, 640; ^{1}H NMR (600 MHz, MeCN-d$_3$, 298 K): δ = 7.49 (d, ^3J = 9.0 Hz, 2H, H-6, H-6'), 7.40 (d, ^3J = 9.0 Hz, 2H, H-8, H-8'), 7.04 (d, ^3J = 9.0 Hz, 2H, H-9, H-9'), 6.89 (d, ^3J = 9.0 Hz, 2H, H-7, H-7'), 5.60 (s, 1H, NH), 5.50 (s, 1H, NH), 5.36 (d, $^3J_{1,2}$ = 2.80 Hz, 1H, H-1), 4.89 (t, $^3J_{3,4}$ = 3.18 Hz, $^3J_{2,3}$ = 3.89 Hz, 1H, H-3), 4.29 (dd, $^3J_{4,5a}$ = 1.67 Hz, $^2J_{5a,5b}$ = 12.0 Hz, 1H, H-5a), 3.71 (dd, $^3J_{2,3}$ = 3.89 Hz,

$^3J_{1,2}$ = 2.80 Hz, 1H, H-2), 3.54 (ddd, $^3J_{3,4}$ = 3.18 Hz, $^3J_{4,5b}$ = 1.90 Hz, $^3J_{4,5a}$ = 1.67 Hz, 1H, H-4), 3.49 (dd, $^3J_{4,5b}$ = 1.90 Hz, $^2J_{5a,5b}$ = 12.0 Hz, 1H, H-5b) ppm; ^{13}C NMR (125 MHz, MeCN-d$_3$, 298 K): δ = 162.74 (C-14), 157.08 (C-12), 156.81 (C-10), 133.47 (2C, C-6, C-6'), 133.26 (2C, C-8, C-8'), 119.45 (2C, C-9, C-9'), 118.97 (2C, C-7, C-7'), 114.71 (C-11), 114.25 (C-13), 99.13 (C-1), 68.51 (C-3), 62.01 (C-5), 48.13 (C-4), 47.18 (C-2) ppm; HRMS (ESI): calcd. for $[M+Na]^+$ ($C_{18}H_{16}Br_2N_2NaO_4$): 504.9369; found *m/z* = 504.9379.

3 Computational details

All calculations were carried out with a local development version of the ORCA 5.0[3] program package. Geometries were obtained from geometry optimizations at the B3LYP-D4 and ωB97M-D4 level of theory (def2-TZVPP basis set, RI approximation with def2 aux basis sets and tight optimization and convergence criteria)[4, 5, 6,7]. Spin-spin coupling constants were computed at the DFT/pc-J3[8] level of theory (RI with Autoaux basis set and tight convergence criteria). The functionals applied were PBE, TPSS, TPSSH, PBE0 and B3LYP.[4, 9, 10, 11,12] Note that all contributions to the coupling constants (Fermi contact, paramagnetic and diamagnetic spin-orbit as well as spin-dipole) are included. NMR Chemical shifts were obtained at the DLPNO-MP2/pcSseg-3[13, 14] level of theory (no frozen core approximation, RIJCOSX approximation with def2/JK and cc-pwCVQZ/C aux basis set and very tight convergence criteria) using gauge including atomic orbitals. Single point energies were obtained at the DLPNO-CCSD(T1)/cc-pVTZ[15, 16] level of theory (RI approximation with the corresponding JK and C basis sets, normal PNO settings). When mentioned in the manuscript, implicit solvation effects for energies, geometries and properties were included using the CPCM model as implemented in ORCA 5.0[3].

In order to find low energy conformer structures for compound **1** and **2**, several possible conformers were used as starting structures for the CREST protocol at the XTB level of theory.[17] The resulting 20 lowest energy conformers and some additional non $^{1}C_{4}$ and $^{4}C_{1}$ structures were then further optimized at the B3LYP-D4/def2-tzvpp and ωB97M-D4/def2-TVPP level of theory.

Association energy for compound 2 with $ZnCl_2$ to yield 3

In order to assess the energy for the association of compound **2** with $ZnCl_2$ to constrain it to the $^{1}C_4$ conformation, the geometries of **2**, the adduct **3** and $ZnCl_2$ were optimized at the B3LYP-D4/def-TZVPP + CPCM (MeCN) level of theory, and their relative energies and free energies were evaluated. The free energy difference for the reaction **2** + $ZnCl_2$ → **3** amounts to 98.4 kJ/mol. Despite uncertainties in the DFT results this large exothermic free energy is a strong indication for a quantitative reaction in this case.

Computed energy differences for different conformers of compound 1 and 2

In order to assess the relative free energies for the lowest energy conformers found in the CREST conformational search, the ensemble of structures has been optimized using B3LYP-D4 and ωB97M-D4 (def2-TZVPP) including. For the structures with lowest electronic energy for each conformation, a normal mode analysis yielding free energy corrections has been carried out. For the lowest energy structures or each conformer, a DLPNO-CCSD(T1)/cc-pVTZ single point energies were computed.

Table S1. Electronic energy ranking for compound **2** of B3LYP-D4 CPCM (MeCN), ωB97M-D4 CPCM (MeCN) and B3LYP-D4 (no CPCM) obtained with default SCF and optimization criteria.

**B3LYP-D4 CPCM (MeCN)**			**ωB97m-D4 CPCM (MeCN)**			**B3LYP-D4**		
**struc.**	**conf.**	**energy**	**struc.**	**conf.**	**energy**	**struc.**	**conf.**	**energy**
27	$^4C_1$	-6141.802726	27	$^4C_1$	-6142.835719	5	$^1C_4$	-6141.780966
25	$^4C_1$	-6141.802724	25	$^4C_1$	-6142.835719	2	$^1C_4$	-6141.780892
26	$^4C_1$	-6141.802038	26	$^4C_1$	-6142.835209	26	$^4C_1$	-6141.780758
29	$^4C_1$	-6141.802034	29	$^4C_1$	-6142.835204	29	$^4C_1$	-6141.780752
15	$^1C_4$*	-6141.801947	2	$^1C_4$	-6142.834127	27	$^4C_1$	-6141.780738
16	$^1C_4$	-6141.801941	3	$^1C_4$	-6142.833735	25	$^4C_1$	-6141.780733
2	$^1C_4$	-6141.801798	17	$^1C_4$	-6142.833292	6	$^1C_4$	-6141.780571
19	$^1C_4$	-6141.801784	18	$^1C_4$	-6142.833279	3	$^1C_4$	-6141.779121
3	$^1C_4$	-6141.801466	15	$^1C_4$	-6142.833202	16	$^1C_4$	-6141.778819
17	$^1C_4$	-6141.800854	16	$^1C_4$	-6142.833189	15	$^1C_4$	-6141.778810
18	$^1C_4$	-6141.800838	9	$^1C_4$	-6142.833169	9	$^1C_4$	-6141.778682
9	$^1C_4$	-6141.800703	19	$^1C_4$	-6142.832993	20	$^1C_4$	-6141.778612
21	$^1C_4$	-6141.800341	20	$^1C_4$	-6142.832970	21	$^1C_4$	-6141.778610
20	$^1C_4$	-6141.800335	21	$^1C_4$	-6142.832968	8	$^1C_4$	-6141.778317
4	$^1C_4$	-6141.799668	4	$^1C_4$	-6142.832001	7	$^1C_4$	-6141.778301
5	$^1C_4$	-6141.799131	5	$^1C_4$	-6142.831642	19	$^1C_4$	-6141.778057
6	$^1C_4$	-6141.799129	6	$^1C_4$	-6142.831641	4	$^1C_4$	-6141.777685

24	$^{1}C_{4}$	-6141.798983	8	$^{1}C_{4}$	-6142.831272	18	$^{1}C_{4}$	-6141.777588
23	$^{1}C_{4}$	-6141.798977	7	$^{1}C_{4}$	-6142.831271	17	$^{1}C_{4}$	-6141.777576
8	$^{1}C_{4}$	-6141.798791	1	$^{4}C_{1}$	-6142.830587	1	$^{4}C_{1}$	-6141.776904
7	$^{1}C_{4}$	-6141.798787	22	$^{1}C_{4}$	-6142.830553	24	$^{1}C_{4}$	-6141.776892
22	$^{1}C_{4}$	-6141.798007	24	$^{1}C_{4}$	-6142.830522	23	$^{1}C_{4}$	-6141.776891
1	$^{4}C_{1}$	-6141.797663	23	$^{1}C_{4}$	-6142.830512	22	$^{1}C_{4}$	-6141.776722
10	$^{1}S_{5}$	-6141.797211	10	$^{1}S_{5}$	-6142.829973	14	$^{2}S_{0}$	-6141.776563

Table S2. Free energies and energy differences for compound **2** after tight optimization and frequency calculation. Energies are given for B3LYP-D4 CPCM (MeCN), ωB97M-D4 CPCM (MeCN) and B3LYP-D4 (no CPCM) levels of theory (tight SCF and optimization criteria).

	**B3LYP-D4 (MeCN)**			**ωB97m-D4 (MeCN)**			**B3LYP-D4**		
conf.	$^{4}C_{1}$	$^{1}C_{4}$	$^{1}S_{5}$	$^{4}C_{1}$	$^{1}C_{4}$	$^{1}S_{5}$	$^{4}C_{1}$	$^{1}C_{4}$	$^{2}S_{0}$
G	-6141.523711	-6141.520807	-6141.517673	-6142.553639	-6142.550937	-6142.547505	-6141.500779	-6141.500065	-6141.496376
ΔG [kJ/mol]	0.00	7.62	15.85	0.00	7.09	16.11	0.00	1.88	11.56
DLPNO-CCSD(T1) energies	-6137.552587	-6137.552239	-6137.547805	-6137.552382	-6137.552710	-6137.547680	-6137.531472	-6137.532361	-6137.527432
ΔE [kJ/mol]	0.00	0.91	12.55	0.00	-0.86	12.35	0.00	-2.33	10.61
DLPNO-CCSD(T1) energies + DFT free energy corrections	-6137.273573	-6137.271099	-6137.268267	-6137.270303	-6137.269519	-6137.265211	-6137.251493	-6137.251458	-6137.247240
ΔG [kJ/mol]	0.00	6.50	13.93	0.00	2.06	13.37	0.00	0.09	11.17

Table S3. Electronic energy ranking for compound **1** of B3LYP-D4 CPCM (MeCN), ωB97M-D4 CPCM (MeCN) and B3LYP-D4 (no CPCM) obtained with default SCF and optimization criteria.

**B3LYP-D4 (MeCN)**			**ωB97m-D4 (MeCN)**			**B3LYP-D4**		
**struc.**	**conf.**	**energy**	**struc.**	**conf.**	**energy**	**struc.**	**conf.**	**energy**
13	$^{1}C_{4}$	-6833.427422	1	$^{1}C_{4}$	-6834.990000	12	$^{1}C_{4}$	-6833.400109
2	$^{1}C_{4}$	-6833.427419	12	$^{1}C_{4}$	-6834.989994	1	$^{1}C_{4}$	-6833.400103
12	$^{1}C_{4}$	-6833.426543	17	$^{1}C_{4}$	-6834.989855	2	$^{1}C_{4}$	-6833.399860
1	$^{1}C_{4}$	-6833.426536	13	$^{1}C_{4}$	-6834.989738	13	$^{1}C_{4}$	-6833.399856
7	$^{1}C_{4}$	-6833.426426	2	$^{1}C_{4}$	-6834.989738	19	$^{1}C_{4}$	-6833.399736
19	$^{1}C_{4}$	-6833.426417	18	$^{1}C_{4}$	-6834.989592	7	$^{1}C_{4}$	-6833.399732
6	$^{1}C_{4}$	-6833.426261	16	$^{1}C_{4}$	-6834.989481	16	$^{1}C_{4}$	-6833.399094
17	$^{1}C_{4}$	-6833.426196	5	$^{1}C_{4}$	-6834.989472	5	$^{1}C_{4}$	-6833.399087
4	$^{4}C_{1}$	-6833.426111	14	$^{1}C_{4}$	-6834.989219	18	$^{1}C_{4}$	-6833.399068
15	$^{4}C_{1}$	-6833.426104	3	$^{1}C_{4}$	-6834.989218	14	$^{1}C_{4}$	-6833.398717
5	$^{1}C_{4}$	-6833.425954	19	$^{1}C_{4}$	-6834.988877	3	$^{1}C_{4}$	-6833.398716
16	$^{1}C_{4}$	-6833.425953	7	$^{1}C_{4}$	-6834.988794	20	$^{1}C_{4}$	-6833.398584
18	$^{1}C_{4}$	-6833.425946	15	$^{4}C_{1}$	-6834.988722	8	$^{1}C_{4}$	-6833.398541
14	$^{1}C_{4}$	-6833.425693	4	$^{4}C_{1}$	-6834.988705	11	$^{1}C_{4}$	-6833.398468
3	$^{1}C_{4}$	-6833.425691	6	$^{1}C_{4}$	-6834.988449	6	$^{1}C_{4}$	-6833.397498
8	$^{1}C_{4}$	-6833.424328	23	$^{2}S_{0}$*	-6834.987711	17	$^{1}C_{4}$	-6833.397467
20	$^{1}C_{4}$	-6833.424299	25	$^{2}S_{0}$	-6834.987675	4	$^{4}C_{1}$	-6833.397330
25	$^{2}S_{0}$	-6833.424179	10	$^{2}S_{0}$	-6834.987659	15	$^{4}C_{1}$	-6833.397314
23	$^{2}S_{0}$	-6833.424175	20	$^{1}C_{4}$	-6834.987609	25	$^{2}S_{0}$	-6833.396597
10	$^{2}S_{0}$	-6833.424112	9	$^{2}S_{0}$	-6834.987605	23		-6833.396590

Table S4. Electronic energy ranking for compound **1** of B3LYP-D4 CPCM (DMSO), B3LYP-D4 CPCM (acetone) and B3LYP-D4 CPCM (benzene) obtained with default SCF and optimization criteria.

**B3LYP-D4 (DMSO)**			**B3LYP-D4 (acetone)**			**B3LYP-D4 (benzene)**		
**struc.**	**conf.**	**energy**	**struc.**	**conf.**	**energy**	**struc.**	**conf.**	**energy**
13	$^{1}C_{4}$	-6833.427639	13	$^{1}C_{4}$	-6833.426691	6	$^{1}C_{4}$	-6833.414372
2		-6833.427635	2		-6833.426690	17		-6833.414364
6		-6833.427028	12		-6833.425836	2		-6833.414295
12		-6833.426750	1		-6833.425832	13		-6833.414294
1		-6833.426744	7		-6833.425723	12		-6833.414065
7		-6833.426634	19		-6833.425715	1		-6833.414057
19		-6833.426623	6		-6833.425464	7		-6833.413889
17		-6833.426418	17		-6833.425443	19		-6833.413889
15	$^{4}C_{1}$	-6833.426344	15	$^{4}C_{1}$	-6833.425320	18		-6833.413298
4		-6833.426339	4		-6833.425307	5		-6833.413293
5		-6833.426164	5		-6833.425243	16		-6833.413287
16		-6833.426162	16		-6833.425242	3		-6833.413012
18		-6833.426155	18		-6833.425235	14		-6833.413006
14		-6833.425901	14		-6833.424982	4	$^{4}C_{1}$	-6833.412635
3		-6833.425899	3		-6833.424978	15		-6833.412629
8		-6833.424540	8		-6833.423608	20		-6833.411769
20		-6833.424511	20		-6833.423577	8		-6833.411726
25	$^{2}S_{0}$	-6833.424395	25	$^{2}S_{0}$	-6833.423442	11		-6833.411632
23		-6833.424392	23		-6833.423437	25	$^{2}S_{0}$	-6833.411020
10		-6833.424328	10		-6833.423374	23		-6833.411001

Table S5. Free energies and energy differences for compound **1** after tight optimization and frequency calculation. Energies are given for B3LYP-D4 CPCM (MeCN), ωB97M-D4 CPCM (MeCN) and B3LYP-D4 (no CPCM) levels of theory (tight SCF and optimization criteria).

	**B3LYP-D4 (MeCN)**			**ωB97m-D4 (MeCN)**			**B3LYP-D4**		
conf.	$^{1}C_{4}$	$^{4}C_{1}$	$^{2}S_{O}$	$^{1}C_{4}$	$^{4}C_{1}$	$^{2}S_{O}$	$^{1}C_{4}$	$^{4}C_{1}$	$^{2}S_{0}$
G	-6832.910740	-6832.911508	-6832.909078	-6834.470631	-6834.469845	-6834.468003	-6832.883041	-6832.880111	-6832.879646
ΔG [kJ/mol]	0.00	-2.02	4.36	0.00	2.06	6.90	0.00	7.69	8.91
DLPNO-CCSD(T1) energies	-6828.131832	-6828.130480	-6828.128894	-6828.130969	-6828.129667	-6828.128782	-6828.106929	-6828.102974	-6828.102388
DLPNO-CCSD(T1) energies + DFT free energy corrections	-6827.615154	-6827.615891	-6827.613793	-6827.611600	-6827.610791	-6827.609072	-6827.589862	-6827.585759	-6827.585436
ΔG [kJ/mol]	1.94	0.00	5.51	-2.12	0.00	4.51	-10.77	0.00	0.85

Table S6. Free energies and energy differences for compound **1** after tight optimization and frequency calculation. Energies are given for B3LYP-D4 CPCM (DMSO), B3LYP-D4 CPCM (acetone) and B3LYP-D4 CPCM (benzene) levels of theory.

	**B3LYP-D4 (DMSO)**			**B3LYP-D4 (acetone)**			**B3LYP-D4 (benzene)**		
conf.	$^{1}C_{4}$	$^{4}C_{1}$	$^{2}S_{O}$	$^{1}C_{4}$	$^{4}C_{1}$	$^{2}S_{O}$	$^{1}C_{4}$	$^{4}C_{1}$	$^{2}S_{0}$
G	-6832.910965	-6832.911705	-6832.909293	-6832.909975	-6832.910648	-6832.908309	-6832.897350	-6832.896465	-6832.894861
ΔG [kJ/mol]	0.00	1.94	4.39	0.00	-1.77	4.37	0.00	2.32	4.21
DLPNO-CCSD(T1) energies	-6828.132034	-6828.130713	-6828.129072	-6828.131136	-6828.129706	-6828.128241	-6828.119734	-6828.117579	-6828.116993
DLPNO-CCSD(T1) energies + DFT free energy corrections	-6827.615366	-6827.616089	-6827.613971	-6827.614423	-6827.615044	-6827.613107	-6827.602711	-6827.601411	-6827.600832
ΔG [kJ/mol]	1.90	0.00	5.56	1.63	0.00	5.08	-3.41	0.00	1.52

Note that an additional file containing all relevant geometries and further computational data is available for download along with the supporting information

^{1}H chemical shifts

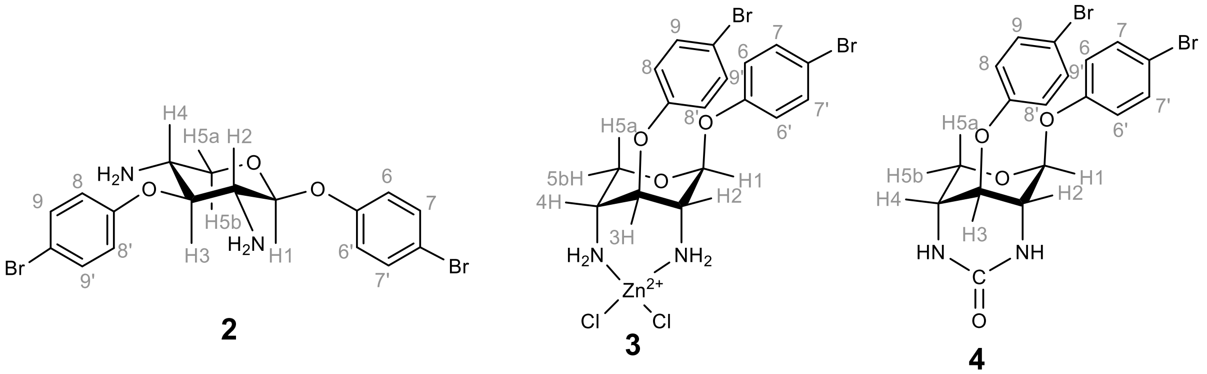

2 **3** **4**

Figure S1. Proton numbering for the xylosides **2**, **3**, and **4**.

Table S7. Chemical shifts (in ppm) for protons of xylosides **2**, **3**, and **4** in MeCN-d_3 and comparison to the calculated data. Protons were assigned by 2D NMR experiments (COSY and HSQC). Calculated values were obtained at the DLPNO-MP2/pcSseg-3 + CPCM (MeCN) (based on B3LYP-D4/def2-TZVPP + CPCM (MeCN) geometries) levels of theory and are displayed in grey columns.

Proton	Int.	2 (exp)	2 (calc.)	3 (exp)	3 (calc.)	4 (exp)	4 (calc.)
2	1	3.02	3.46	3.51	3.66	3.71	3.98
4	1	3.05	2.23	3.37	3.47	3.54	3.65
5b	1	3.34	2.76	3.65	3.83	3.49	3.80
5a	1	3.91	3.29	4.36	4.48	4.29	4.58
3	1	4.03	3.46	4.52	4.84	4.89	5.16
1	1	4.84	4.29	5.50	5.77	5.36	5.50
7, 7‘	2	6.99	7.58	7.01	7.78	6.89	7.69
9, 9‘	2	7.08	7.55	7.07	7.86	7.04	7.87
8, 8‘	2	7.41	7.13	7.49	7.69	7.41	7.67
6, 6‘	2	7.45	7.01	7.52	7.56	7.49	7.34

^{13}C chemical shifts

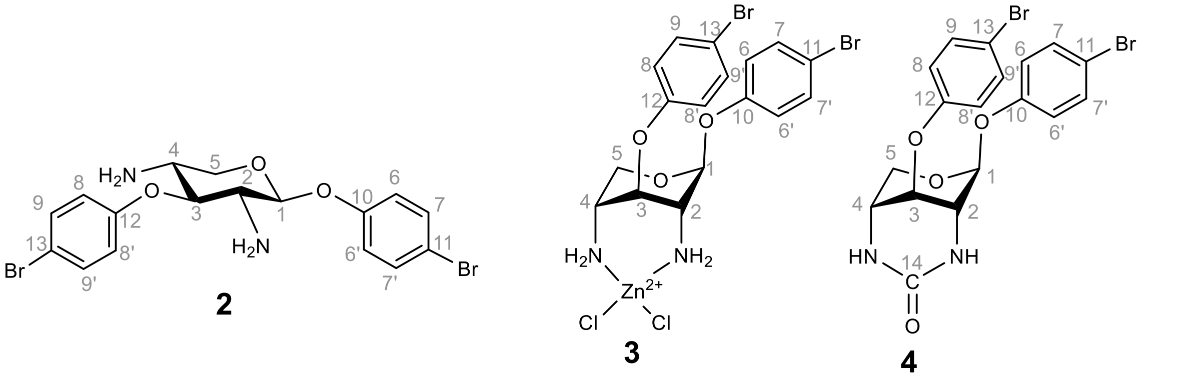

Figure S2. Carbon numbering for the xylosides **2**, **3**, and **4**.

Table S8. Chemical shifts (in ppm) of the carbons of xylosides **2**, **3**, and **4** in MeCN-d$_3$ and comparison to the calculated data. Carbons were assigned by 2D NMR experiments (HSQC). Calculated values were obtained at the DLPNO-MP2/pcSseg-3 + CPCM (MeCN) (based on B3LYP-D4/def2-TZVPP + CPCM (MeCN) geometries) levels of theory and are displayed in grey columns.

Carbon	Int.	2 (exp)	2 (calc.)	3 (exp)	3 (calc.)	4 (exp)	4 (calc.)
4	1	53.48	55.36	48.34	56.52	48.13	56.38
2	1	57.99	58.64	49.44	53.18	47.18	52.50
5	1	67.76	68.62	59.07	65.46	62.01	69.32
3	1	86.34	88.52	72.21	76.46	68.57	74.58
1	1	103.42	99.40	97.00	104.20	99.13	108.72
13	1	113.68	135.51	n.d.	134.73	114.25	134.23
11	1	115.13	1136.37	n.d.	134.63	114.71	134.31
7, 7‘	2	119.56	149.72	119.66	136.90	118.97	136.82
9, 9‘	2	119.68	149.74	119.82	137.19	119.45	137.04
8, 8‘	2	133.15	129.50	133.45	124.90	133.26	124.98
6, 6‘	2	133.31	128.28	133.53	124.66	133.47	124.41
10	1	157.71	172.46	156.22	163.29	156.81	164.93
12	1	160.90	176.74	156.45	163.65	157.08	164.66
14	1	-	-	-	-	157.27	165.87

Computed spin-spin coupling constants

In order to assess which level of theory gives the best agreement with the experimental results, we have computed all components of the isotropic spin-spin coupling constants for the benchmark compounds **3** and **4**. The main influences in the computed values are the level of theory for the geometry optimization, the level of theory for the calculation of the coupling constants and the usage of implicit solvent corrections. Tables S5 to S8 list the corresponding results at various levels of theory together with the MAD and RMSD with respect to the experimental numbers. Note that if not mentioned otherwise, geometries and couplings have been computed using CPCM (MeCN).

Table S9. Computed spin-spin coupling constants (computed at the DFT/pc-J3 level of theory) for compound **3** based on a B3LYP-D4 geometry with CPCM (MeCN). All values are given in Hz. For comparison, the couplings also have been computed without CPCM on geometries; MAD: maximum absolute deviation, RMSD: root-mean-square deviation.

	**B3LYP-D4 with CPCM (MeCN)**					**B3LYP-D4 without CPCM**
	**PBE**	**PBE0**	**TPSS**	**TPSSH**	**B3LYP**	**PBE**
$J_{1,2}$	1.16	1.30	0.62	0.73	1.37	1.01
$J_{2,3}$	1.90	2.02	1.51	1.61	2.08	1.83
$J_{3,4}$	2.68	2.80	2.38	2.48	2.91	2.60
$J_{4,5a}$	2.86	2.99	2.52	2.63	3.20	3.17
$J_{4,5b}$	1.29	1.48	0.85	0.99	1.57	1.21
$J_{5a,5b}$	-12.86	-14.83	-10.67	-11.87	-14.49	-12.67
**RMSD exp/calc.**	1.15	1.29	1.75	1.45	1.19	1.26
**MAD**	1.64	1.83	2.34	1.94	1.49	1.71

Table S10. Computed spin-spin coupling constants (computed at the DFT/pc-J3 level of theory) for compound **3** based on a ωB97M-D4 geometry with CPCM (MeCN). All values are given in Hz; MAD: maximum absolute deviation, RMSD: root-mean-square deviation.

	**ωB97M-D4 with CPCM (MeCN)**				
	**PBE**	**PBE0**	**TPSS**	**TPSSH**	**B3LYP**
$J_{1,2}$	1.16	1.300	0.61	0.72	1.37
$J_{2,3}$	1.72	1.84	1.32	1.42	1.89
$J_{3,4}$	2.69	2.81	2.41	2.51	2.92
$J_{4,5a}$	2.62	2.75	2.24	2.36	2.95
$J_{4,5b}$	1.48	1.67	1.07	1.21	1.77
$J_{5a,5b}$	-12.82	-14.78	-10.59	-11.79	-14.44
**RMSD exp/calc.**	1.14	1.27	1.76	1.45	1.16
**MAD**	1.82	1.78	2.41	2.12	1.66

Table S11. Computed spin-spin coupling constants (computed at the DFT/pc-J3 level of theory) for compound **4** based on a B3LYP-D4 geometry with CPCM (MeCN). All values are given in Hz. For comparison, the couplings also were computed without CPCM on geometries; MAD: maximum absolute deviation, RMSD: root-mean-square deviation.

	**B3LYP-D4 with CPCM (MeCN)**					**B3LYP-D4 without CPCM**
	**PBE**	**PBE0**	**TPSS**	**TPSSH**	**B3LYP**	**PBE**
$J_{1,2}$	1.94	2.08	1.48	1.60	2.22	1.87
$J_{2,3}$	3.47	3.59	3.41	3.49	3.75	3.46
$J_{3,4}$	4.40	4.54	4.41	4.50	4.77	4.36
$J_{4,5a}$	1.17	1.28	0.61	0.72	1.37	1.14
$J_{4,5b}$	2.26	2.44	1.86	1.99	2.58	2.21
$J_{5a,5b}$	-11.51	-13.43	-8.63	-9.86	-12.99	-11.04
$J_{2,NH}$	5.50	5.42	5.63	5.62	5.73	
$J_{4,NH}$	4.84	4.76	4.89	4.88	5.03	
**RMSD exp/calc.**	0.71	0.91	1.63	1.21	0.86	0.79
**MAD**	1.22	1.43	3.37	2.14	1.59	1.18

Table S12. Computed spin-spin coupling constants (computed at the DFT/pc-J3 level of theory) for compound **4** based on a ωB97M-D4 geometry with CPCM (MeCN). All values are given in Hz; MAD: maximum absolute deviation, RMSD: root-mean-square deviation.

	**ωB97M-D4 with CPCM (MeCN)**				
	**PBE**	**PBE0**	**TPSS**	**TPSSH**	**B3LYP**
$J_{1,2}$	2.07	2.22	1.63	1.75	2.37
$J_{2,3}$	3.41	3.53	3.36	3.44	3.68
$J_{3,4}$	4.38	4.52	4.39	4.48	4.75
$J_{4,5a}$	1.07	1.19	0.53	0.63	1.27
$J_{4,5b}$	2.44	2.62	2.06	2.19	2.78
$J_{5a,5b}$	-11.58	-13.49	-8.73	-9.95	-13.04
$J_{2,NH}$	5.37	5.29	5.48	5.47	5.58
$J_{4,NH}$	4.69	4.61	4.72	4.71	4.87
**RMSD exp/calc.**	0.71	0.93	1.59	1.18	0.88
**MAD**	1.12	1.49	3.27	2.05	1.57

From this set of results, it is already obvious that for none of the couplings computed at PBE and PBE0 level of theory, the maximum absolute deviation (MAD) is never larger than 2 Hz. Overall, PBE is in slightly better agreement with experiment. Comparing the results obtained using B3LYP-D4 and ωB97M-D4 geometries for the PBE couplings, the B3LYP geometries yield results with almost identical MAD and RMSD values. However, as B3LYP-D4 geometries are in better agreement for the relative energies of the conformations for compound **2** (see main text and section below), we have chosen to focus on the B3LYP-D4 geometries for further calculations.

Another important finding is that although a rigorous treatment would require the usage of CPCM throughout all calculations, even without CPCM, the spin-spin couplings are

in good agreement with experiment and omitting CPCM here increases the MAD and RMSD only in the order of 0.1 Hz. So, while the relative energies for the different conformers are very sensitive to solvent effects, the spin-spin coupling constants of the xylose protons are quite insensitive when implicit solvation is used, however, they are very specific to the conformation.

Hence, an efficient yet accurate way to achieve a root mean square deviation of about 1 Hz is to compute the spin-spin coupling constants at the PBE/pc-J3 level of theory based on B3LYP-D4/def2-TZVPP geometries. Note that there are several studies in the literature that find similar results.[18] This way, the spin-spin coupling constants for different conformers can be predicted in a robust and efficient way.

Table S13. The $^{1}C_{4}$:$^{4}C_{1}$ conformer distribution of xyloside **1** in various solvents is given as a result of a least squares fit of the type J^{i}_{exp}(solv.) = a * $J^{i}_{calc}(^{1}C_{4})$ + b $J^{i}_{calc}(^{4}C_{1})$.

	**B3LYP-D4 / B3LYP**		**B3LYP-D4 / PBE**		**B3LYP-D4 (CPCM) / PBE (CPCM)**		**ωB97M-D4 (CPCM) / PBE (CPCM)**	
**Solvent**	**$^{1}C_{4}$:$^{4}C_{1}$**	**RMSD**	**$^{1}C_{4}$:$^{4}C_{1}$**	**RMSD**	**$^{1}C_{4}$:$^{4}C_{1}$**	**RMSD**	**$^{1}C_{4}$:$^{4}C_{1}$**	**RMSD**
DMF-d_7	16:84	0.8	5:95	0.3	5:95	0.4	5:95	0.5
DMSO-d_6	17:83	0.8	5:95	0.5	5:95	0.5	5:95	0.6
Acetone-d_6	22:78	1.2	11:89	0.9	11:89	0.8	11:89	0.7
MeOD-d_3	27:73	0.5	17:83	0.4	16:84	0.5	17:83	0.5
Pyridine-d_5	32:68	0.7	22:78	0.4	21:79	0.5	22:78	0.6
THF-d_8	38:62	0.7	30:70	0.5	29:71	0.6	30:70	0.6
Toluene-d_8	61:39	0.6	54:46	0.3	53:47	0.4	54:46	0.4
Benzene-d_6	62:38	0.7	56:44	0.5	55:45	0.4	55:45	0.5
$CDCl_3$	66:34	0.6	60:40	0.4	59:41	0.3	60:40	0.4
Chlorobenzene-d_5	68:32	0.6	62:38	0.3	61:39	0.3	62:38	0.4

4 NMR spectra of the synthesized compounds

NMR spectroscopy of **1** in different solvents at 298 K gave ^{1}H NMR spectra with broad signals as typical for a dynamic mixture of conformers. In some cases, the resolution of the spectra was improved when the NMR experiment was carried out at 328 K and coupling constants could be determined.

To gain more information about the solvent-dependent conformational equilibrium of **1**, NMR experiments were carried out at different temperatures (328 K to 258 K in $CDCl_3$). However, the coalescence temperature was not reached and hence, separation of the signal sets of the different conformers was not achieved.

For the determination of coupling constants of the xyloside ring protons ^{1}H-^{1}H COSY-DQF spectroscopy was performed. Of the respective spectra (Figures S15-S21, Figure S30 and Figure S33) the chemical shift range of the ring protons (~3 ppm to 6 ppm) is depicted.

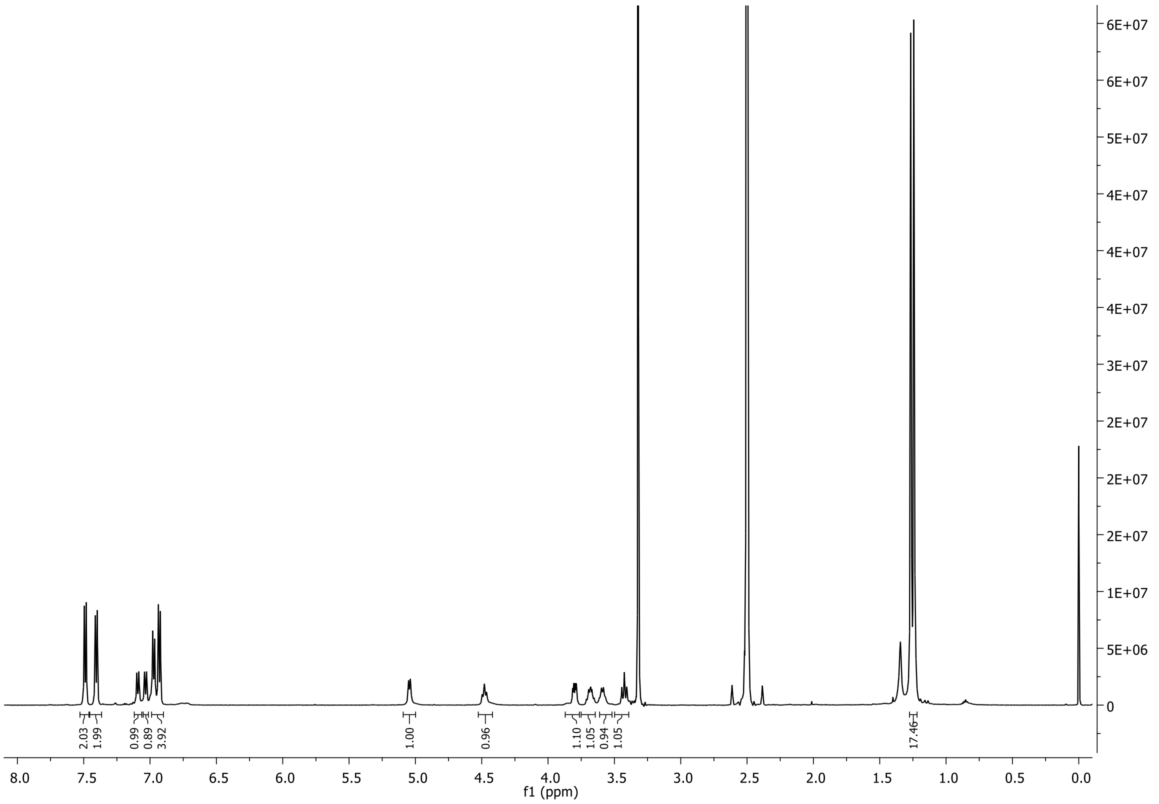

Figure S3. ^{1}H NMR spectrum of **1** (500 MHz, DMSO-d_6, 298 K).

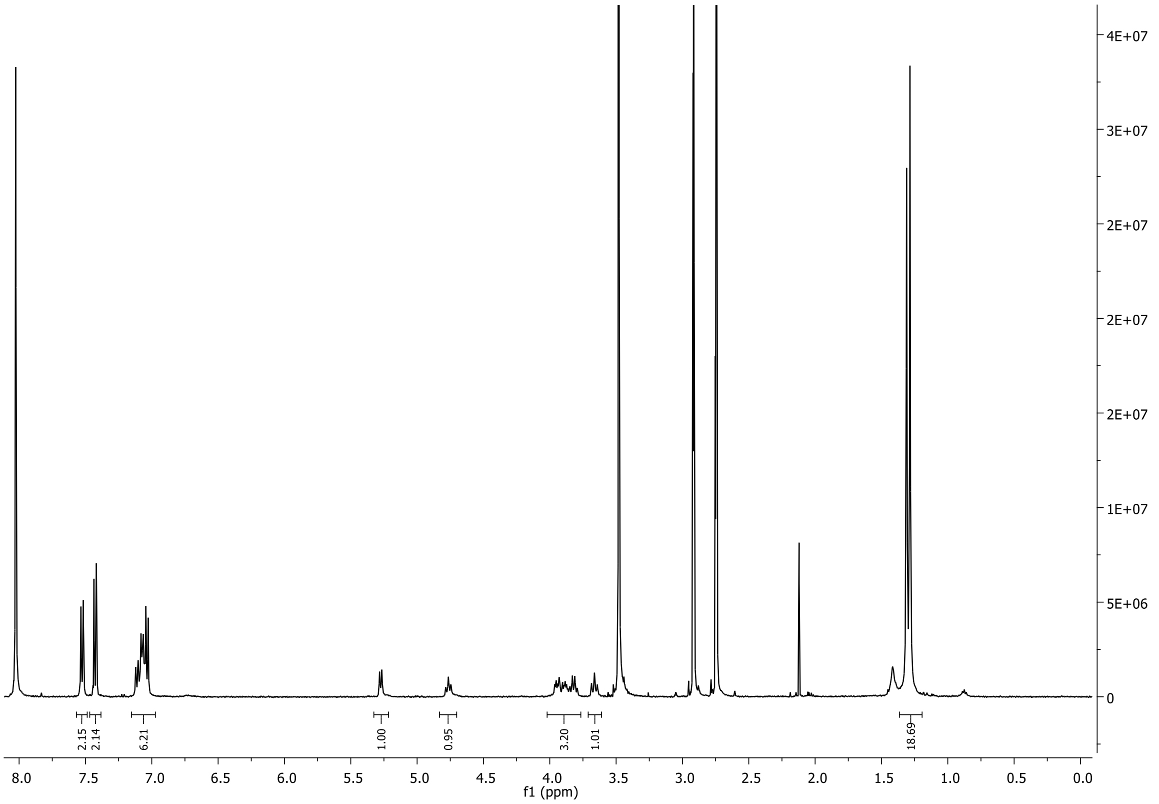

Figure S4. ^{1}H NMR spectrum of **1** (500 MHz, DMF-d_7, 298 K).

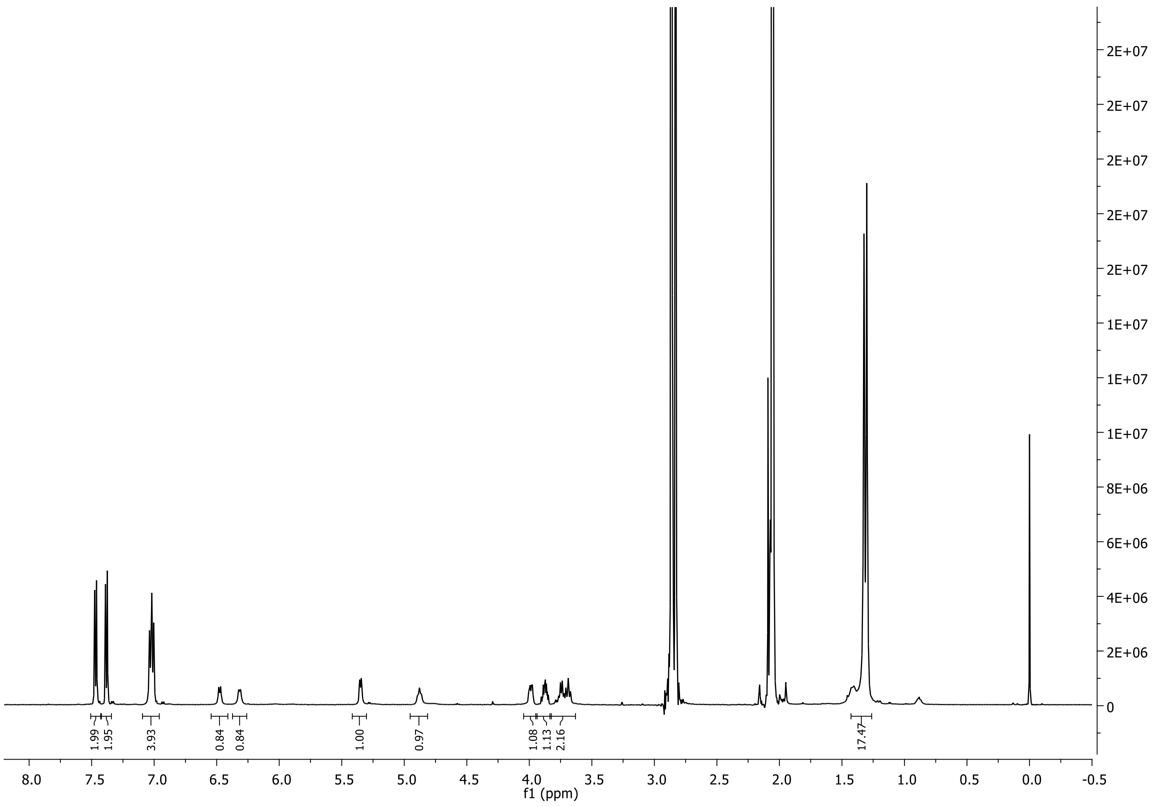

Figure S5. ^{1}H NMR spectrum of **1** (500 MHz, acetone-d_6, 298 K).

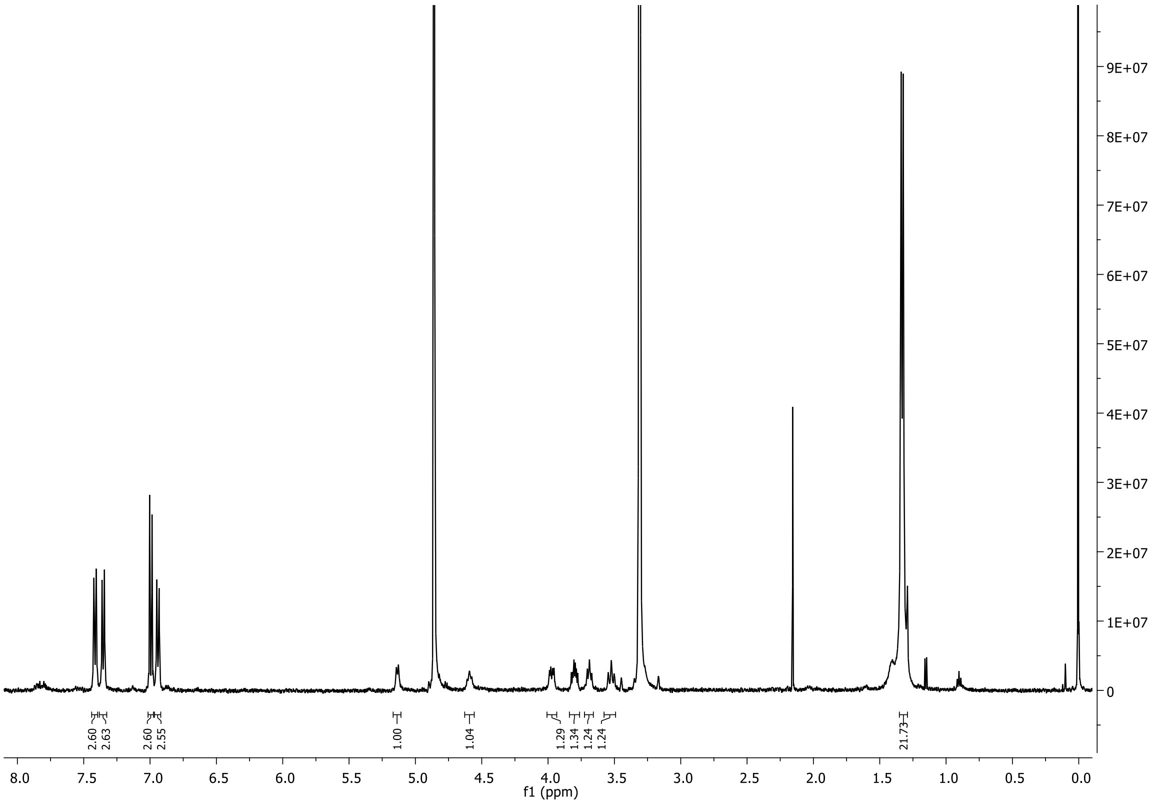

Figure S6. ^{1}H NMR spectrum of **1** (500 MHz, MeOD-d_4, 298 K).

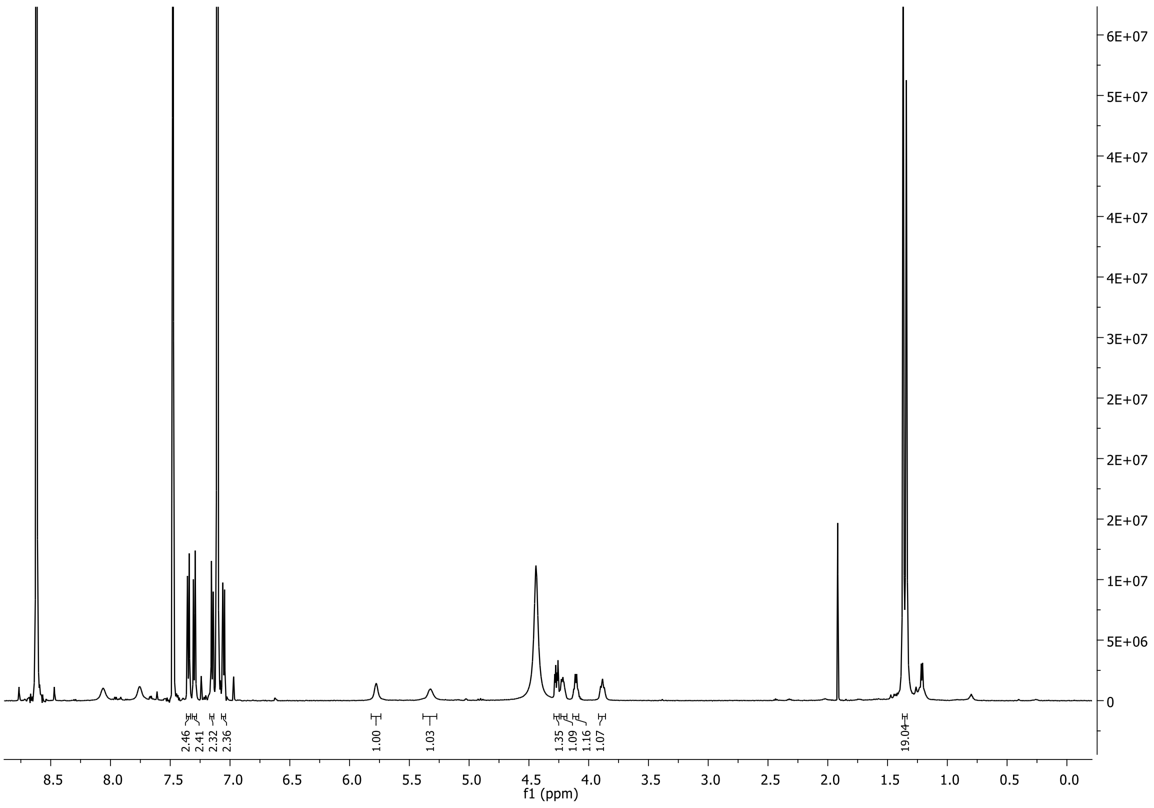

Figure S7. ^{1}H NMR spectrum of **1** (500 MHz, pyridine-d_5, 298 K).

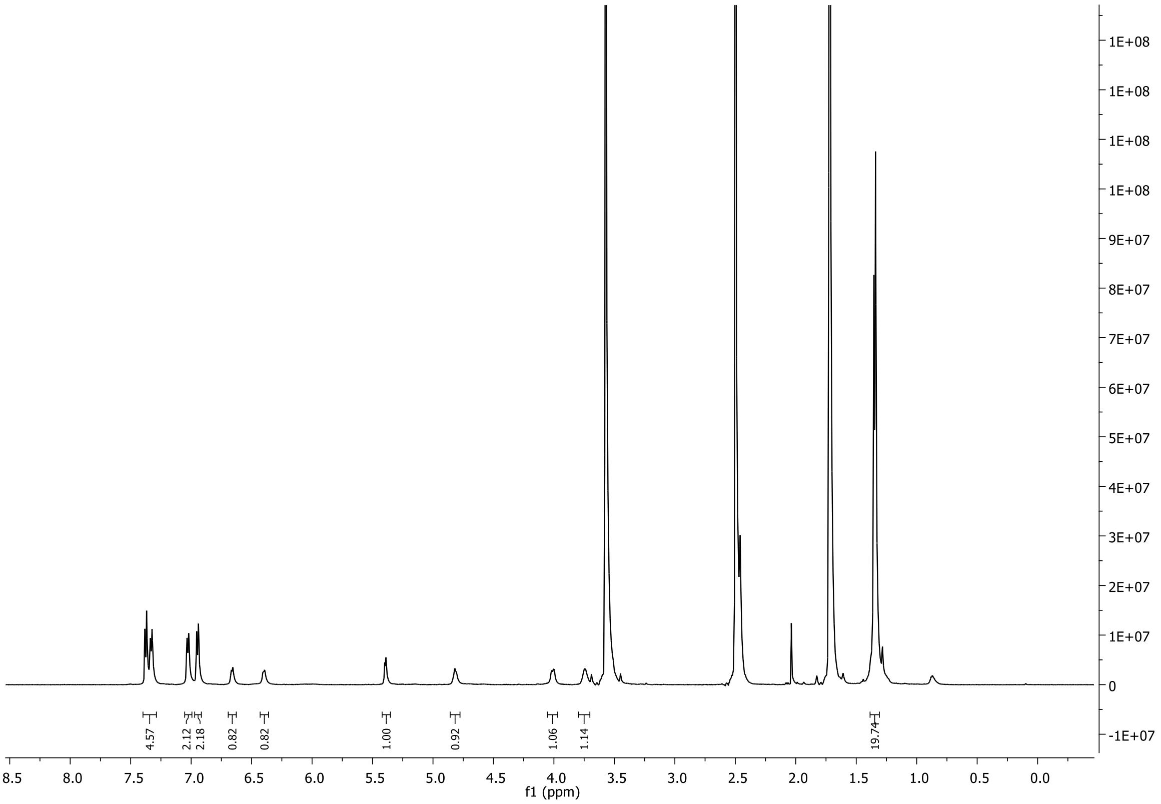

Figure S8. ^{1}H NMR spectrum of **1** (500 MHz, THF-d_8, 298 K).

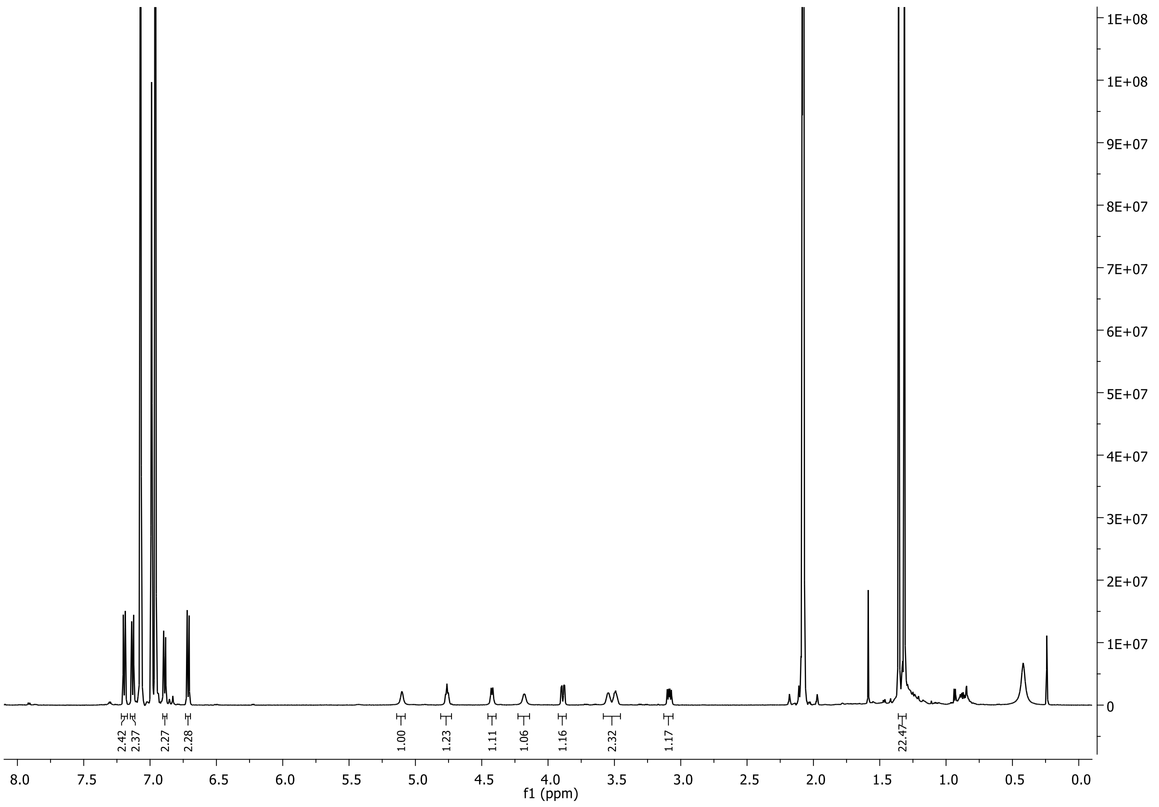

Figure S9. ^{1}H NMR spectrum of **1** (500 MHz, toluene-d_8, 328 K).

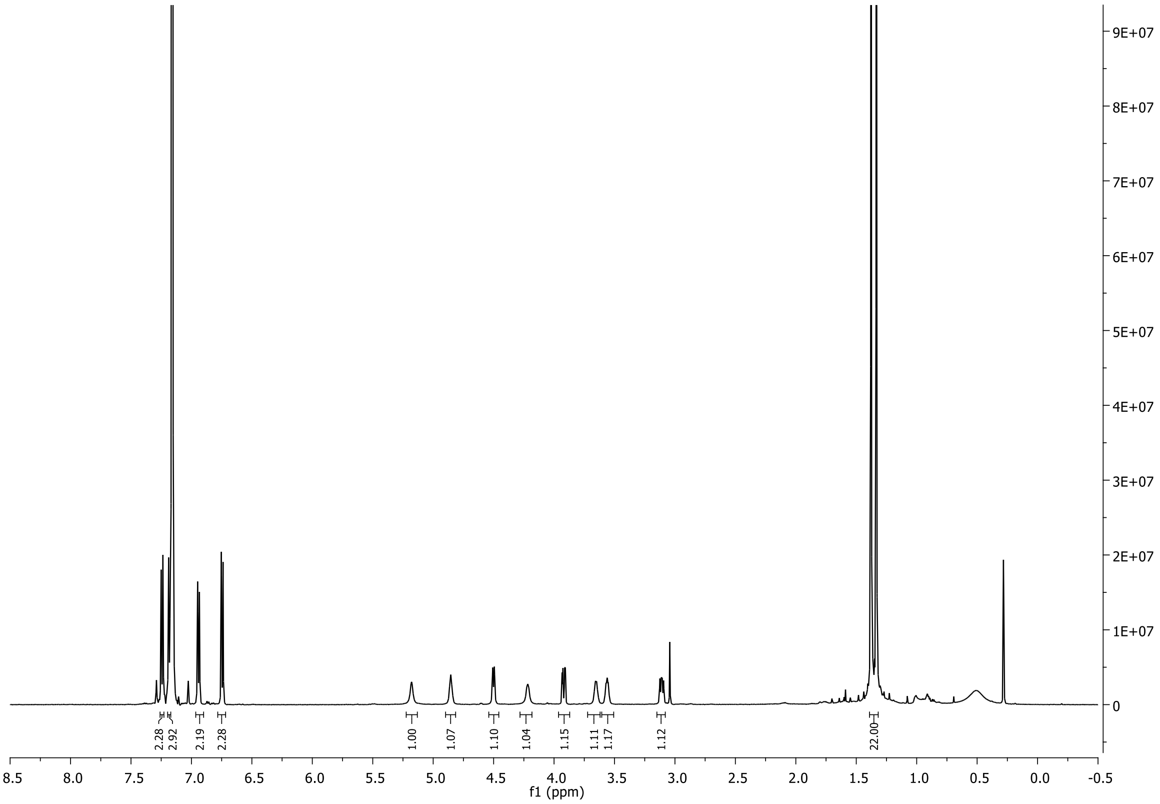

Figure S10. ^{1}H NMR spectrum of **1** (500 MHz, benzene-d_6, 328 K).

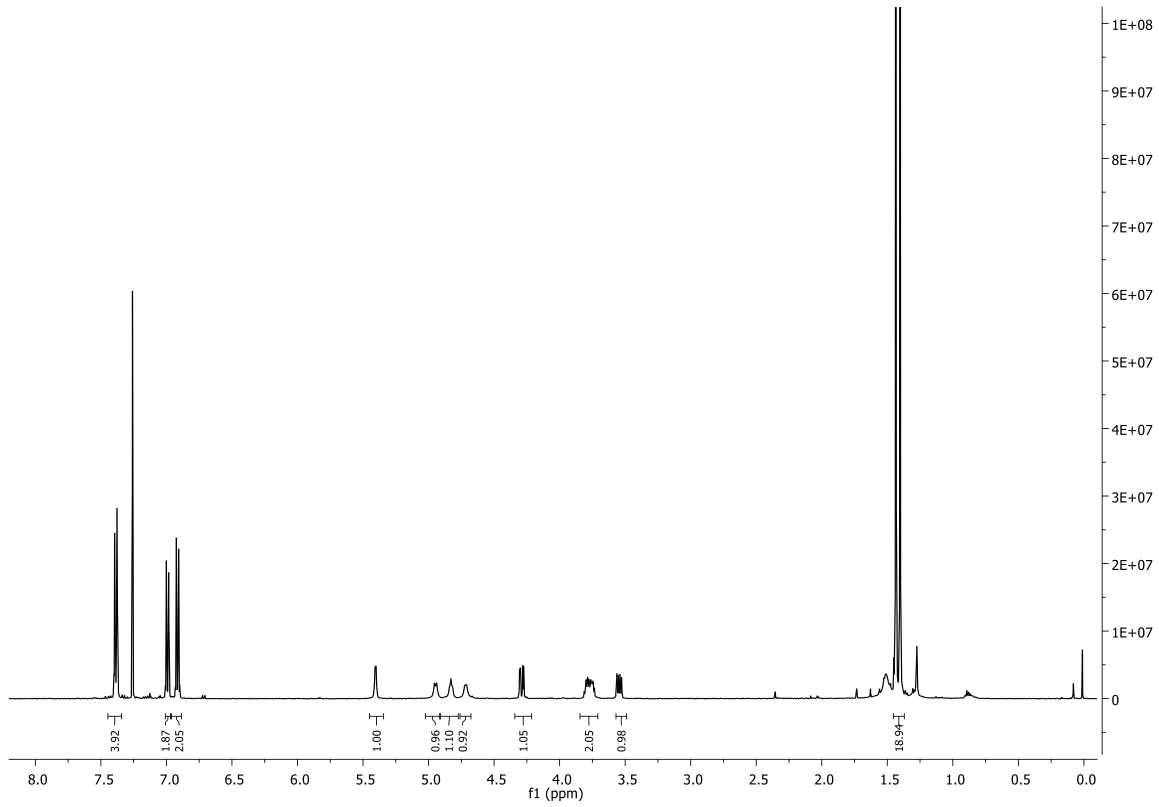

Figure S11. ^{1}H NMR spectrum of **1** (500 MHz, $CDCl_3$, 328 K).

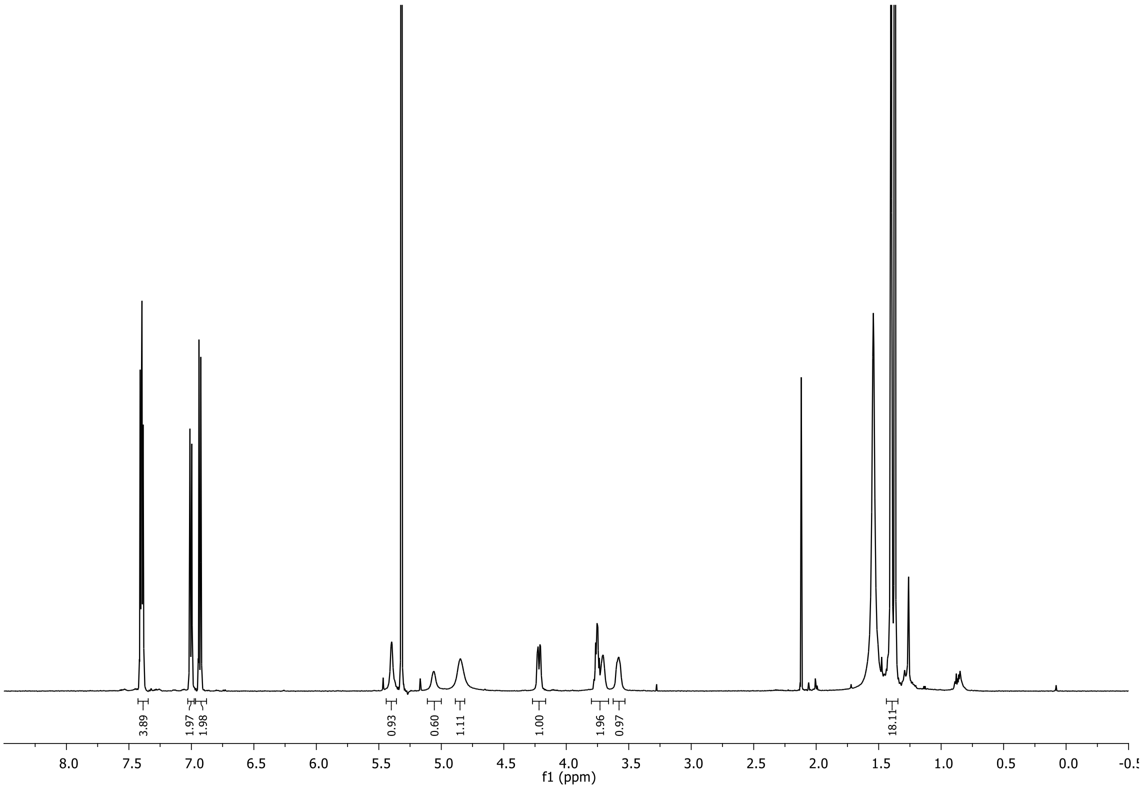

Figure S12. ^{1}H NMR spectrum of **1** (500 MHz, CD_2Cl_2, 298 K).

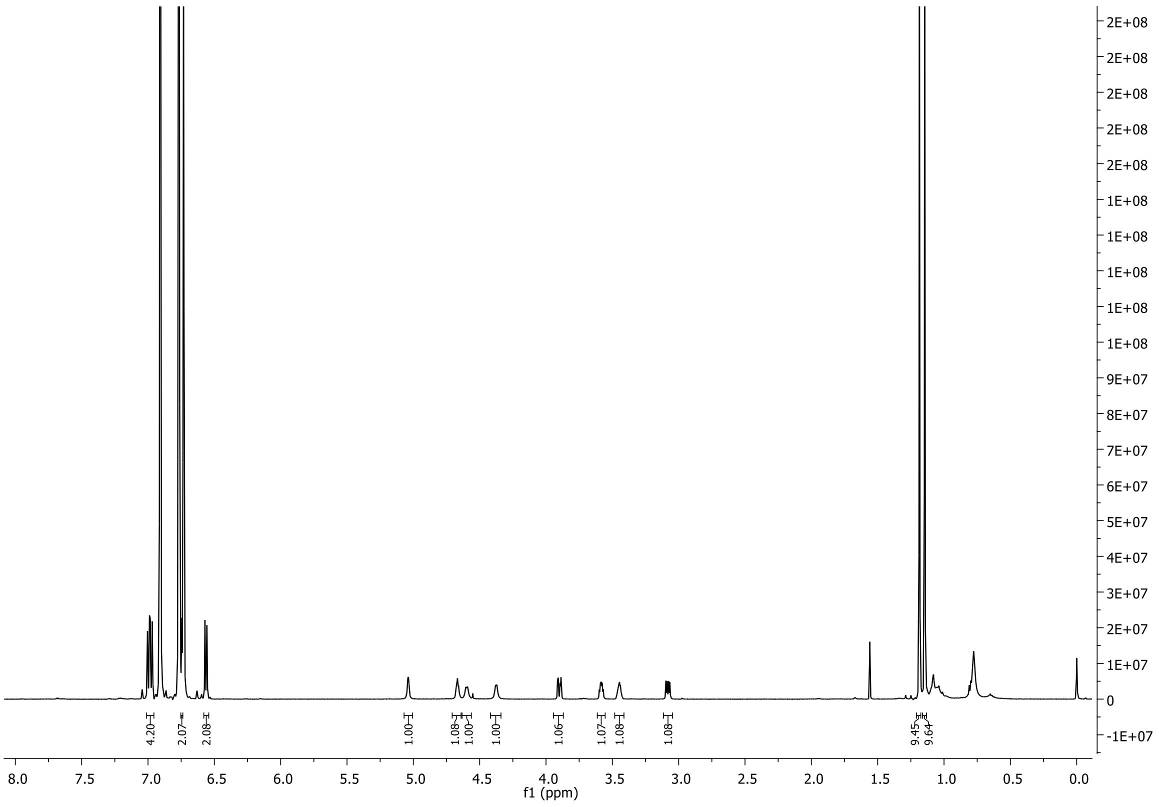

Figure S13. ^{1}H NMR spectrum of **1** (500 MHz, chlorobenzene-d_5, 328 K).

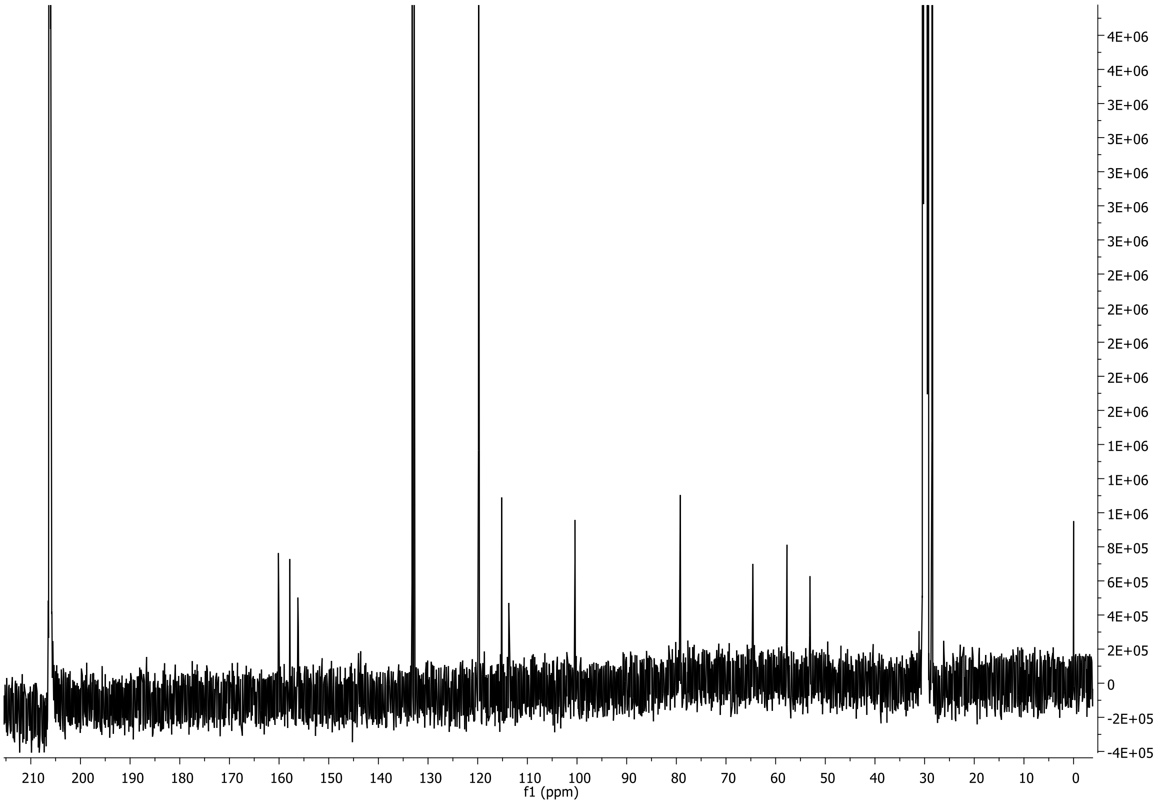

Figure S14. ^{13}C NMR spectrum of **1** (125 MHz, acetone-d$_6$, 298 K).

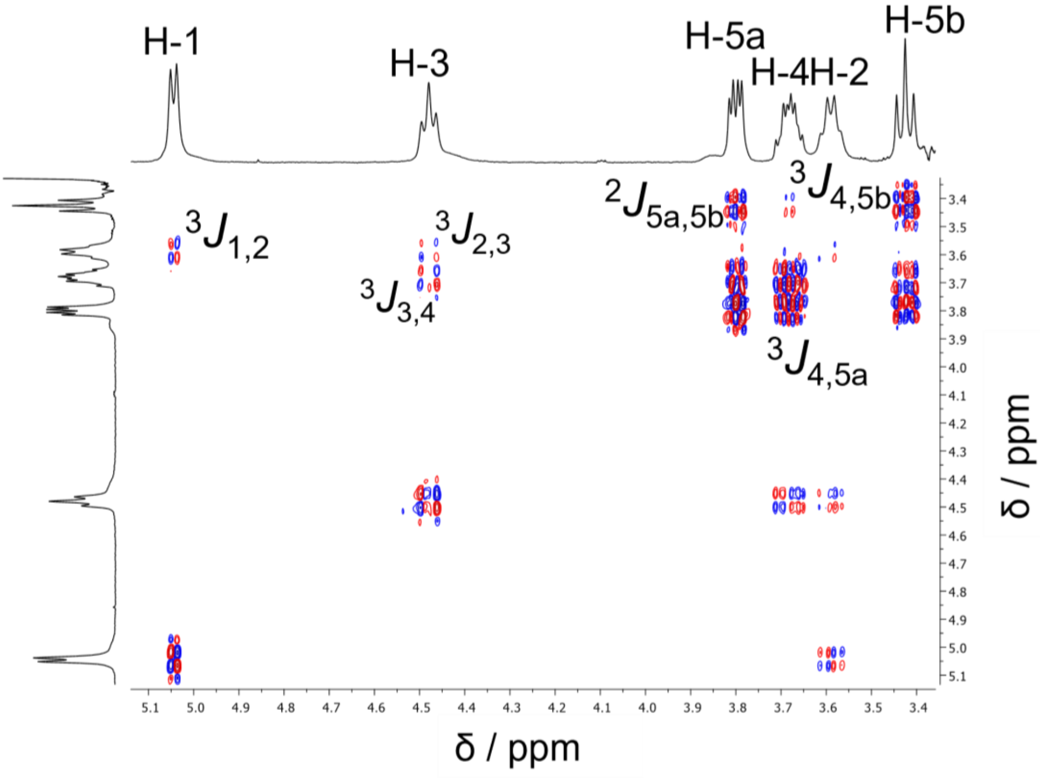

Figure S15. COSY-DQF NMR spectrum of **1** (500 MHz, DMSO-d$_6$, 298 K).

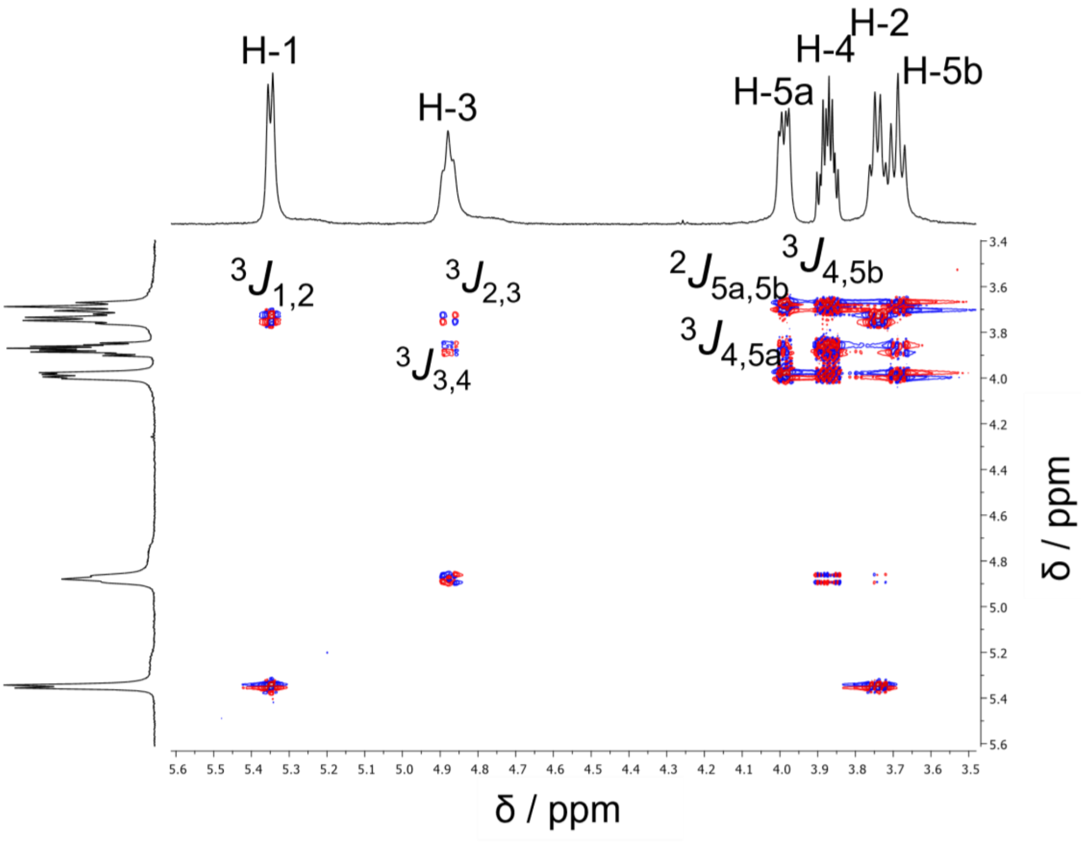

Figure S16. COSY-DQF NMR spectrum of **1** (500 MHz, acetone-d_6, 298 K).

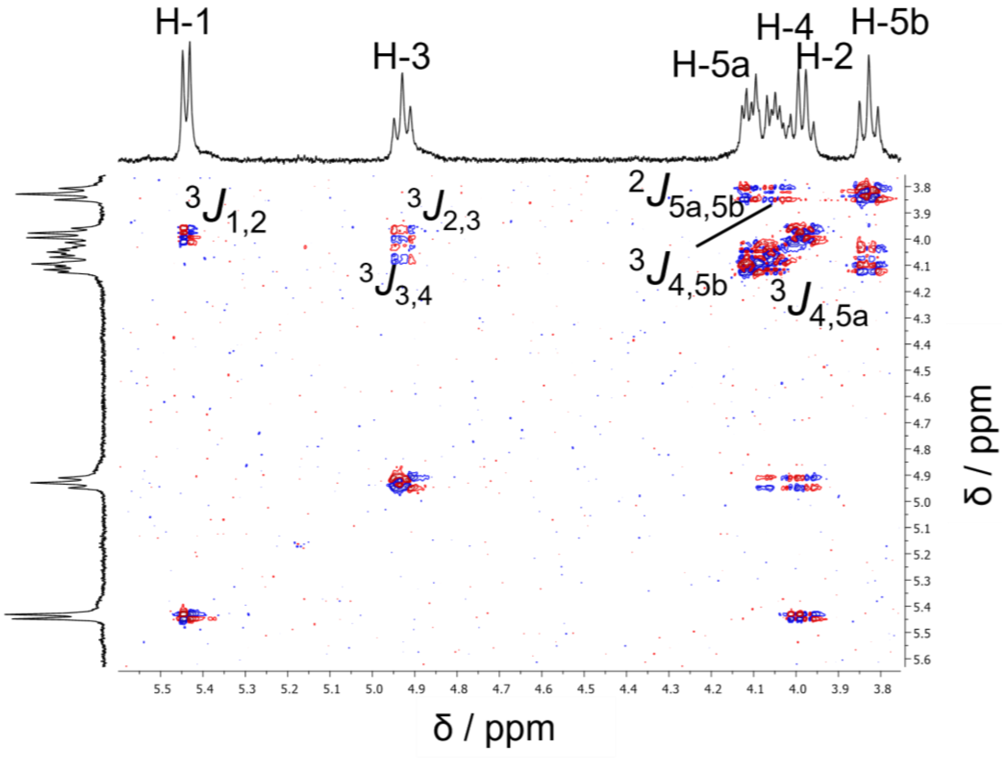

Figure S17. COSY-DQF NMR spectrum of **1** (500 MHz, DMF-d_7, 298 K).

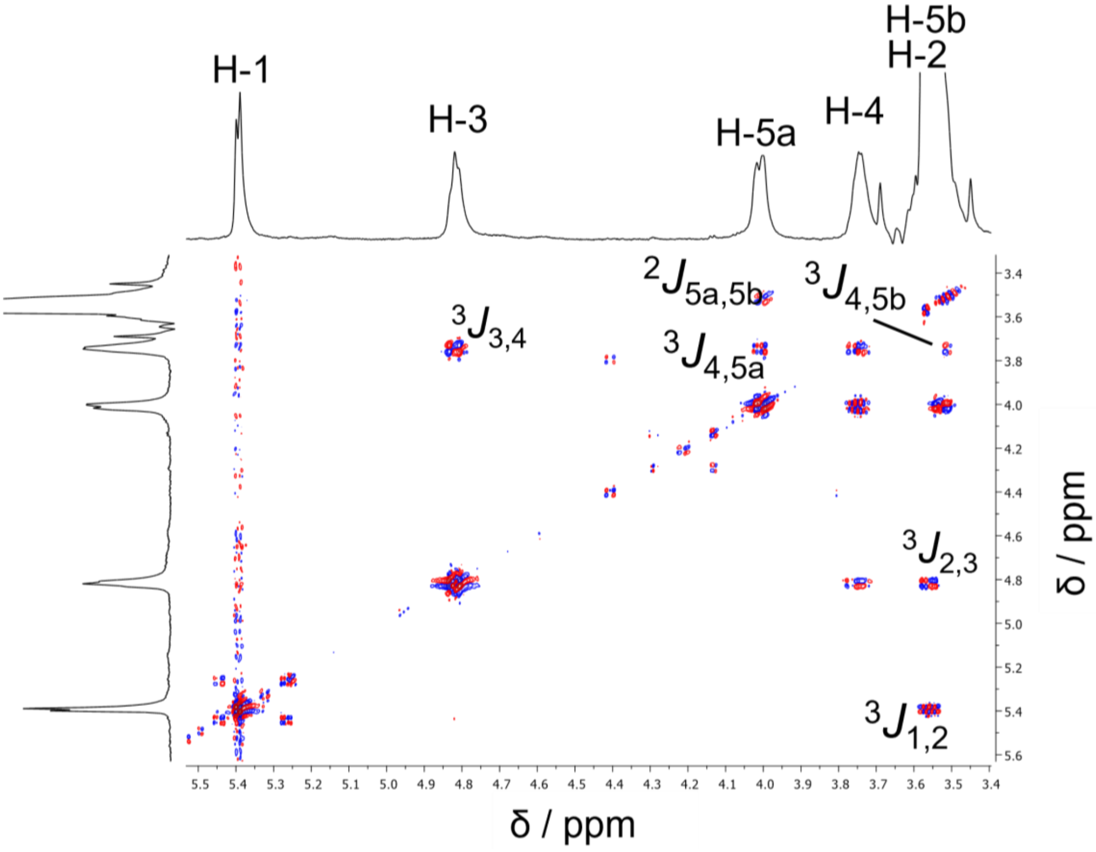

Figure S18. COSY-DQF NMR spectrum of **1** (500 MHz, THF-d_8, 298 K).

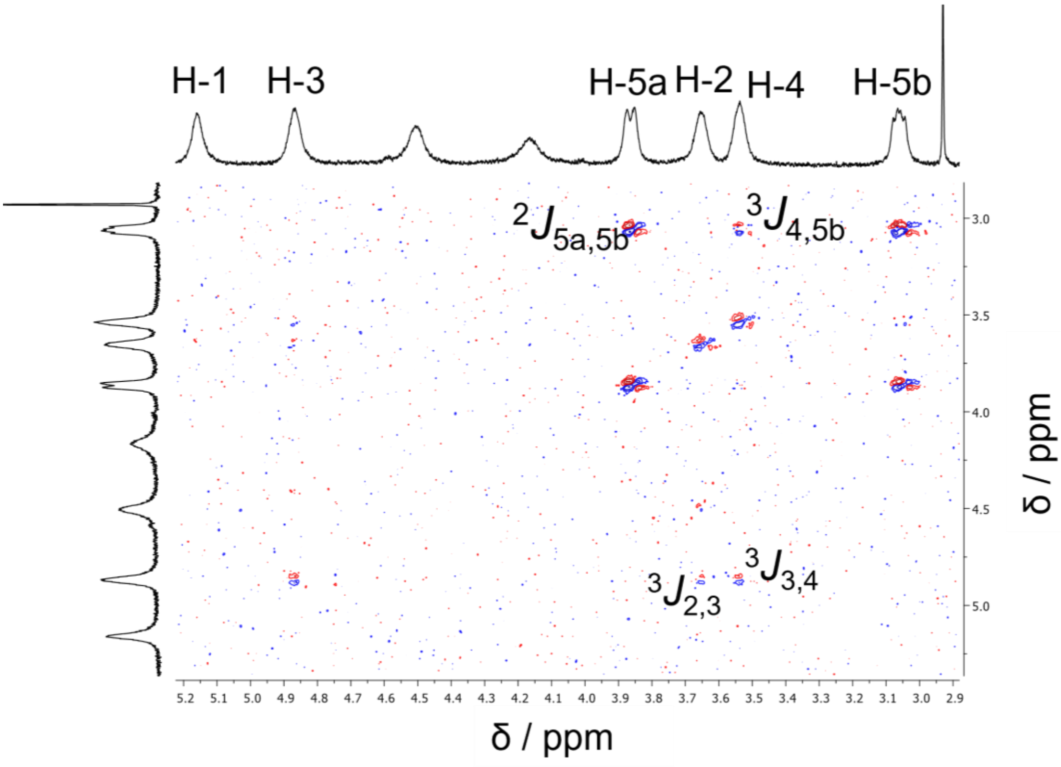

Figure S19. COSY-DQF NMR spectrum of **1** (500 MHz, benzene-d_6, 298 K).

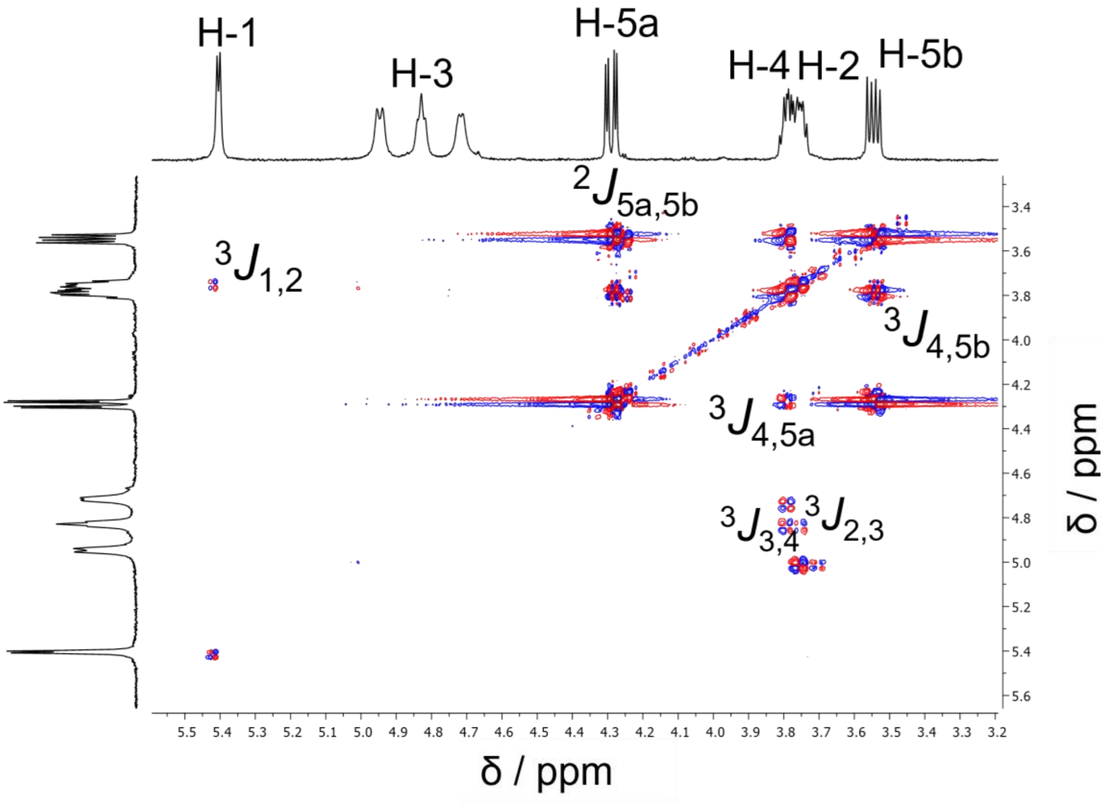

Figure S20. COSY-DQF NMR spectrum of **1** (500 MHz, $CDCl_3$, 298 K).

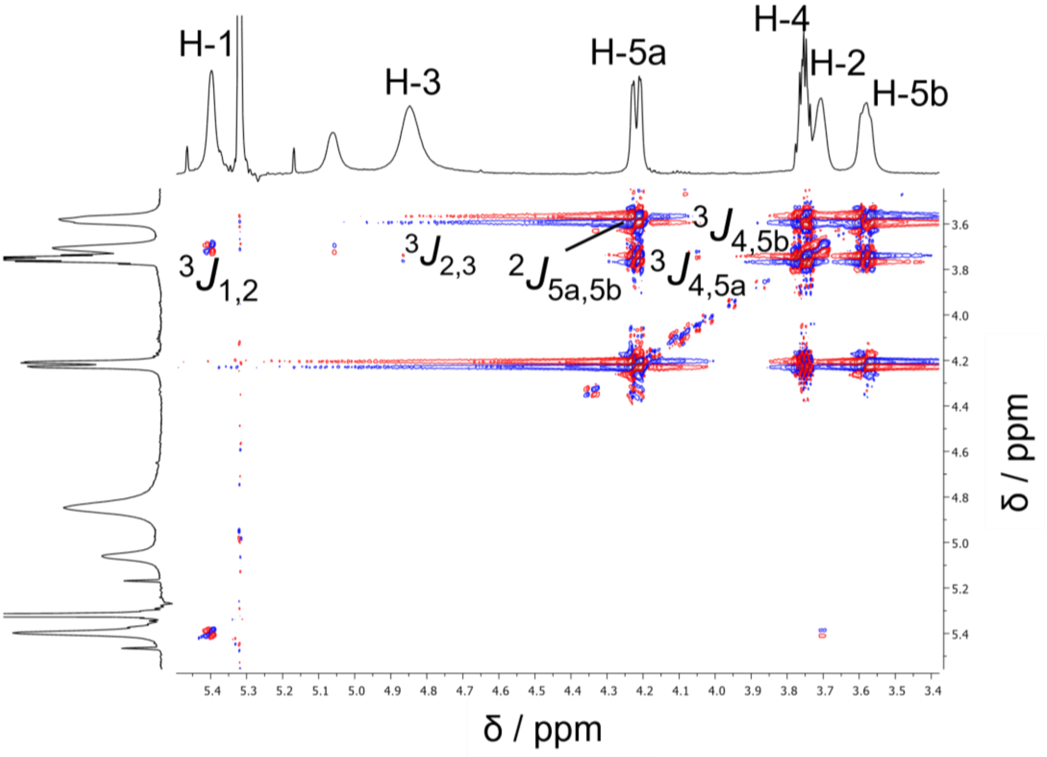

Figure S21. COSY-DQF NMR spectrum of **1** (500 MHz, CD_2Cl_2, 298 K).

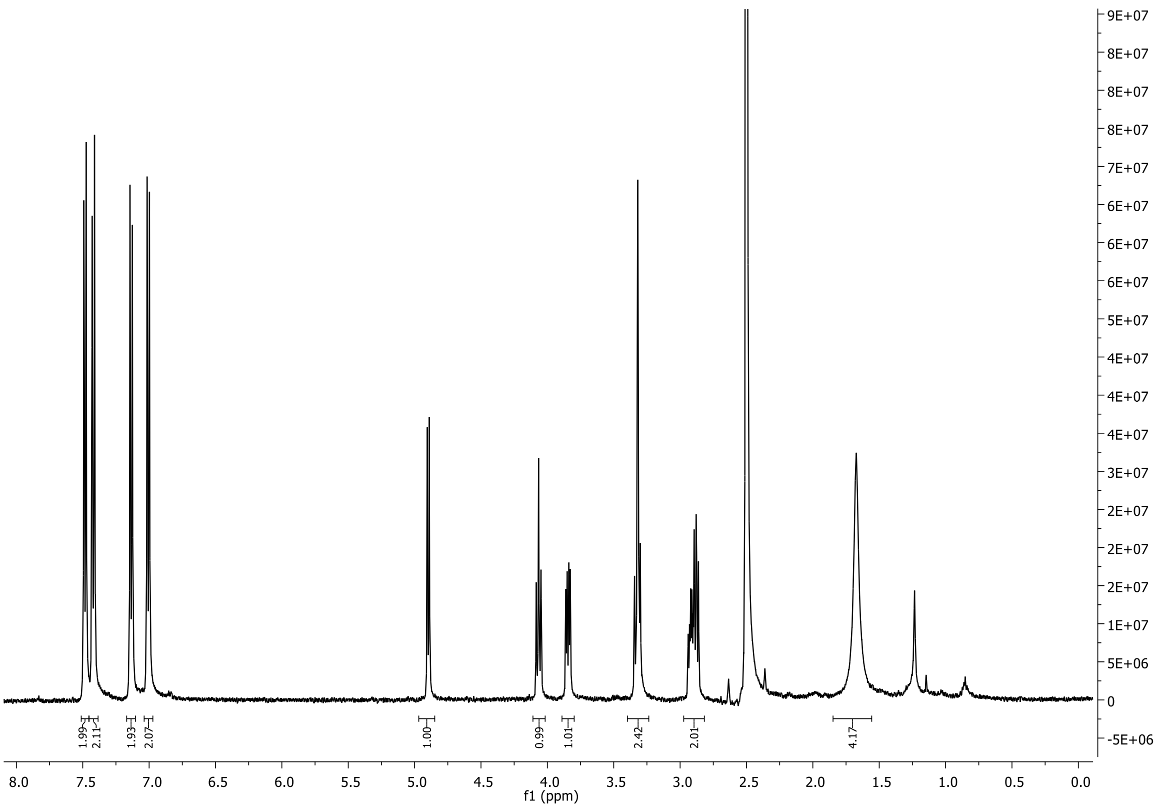

Figure S22. ^{1}H NMR spectrum of **2** (500 MHz, DMSO-d$_6$, 298 K).

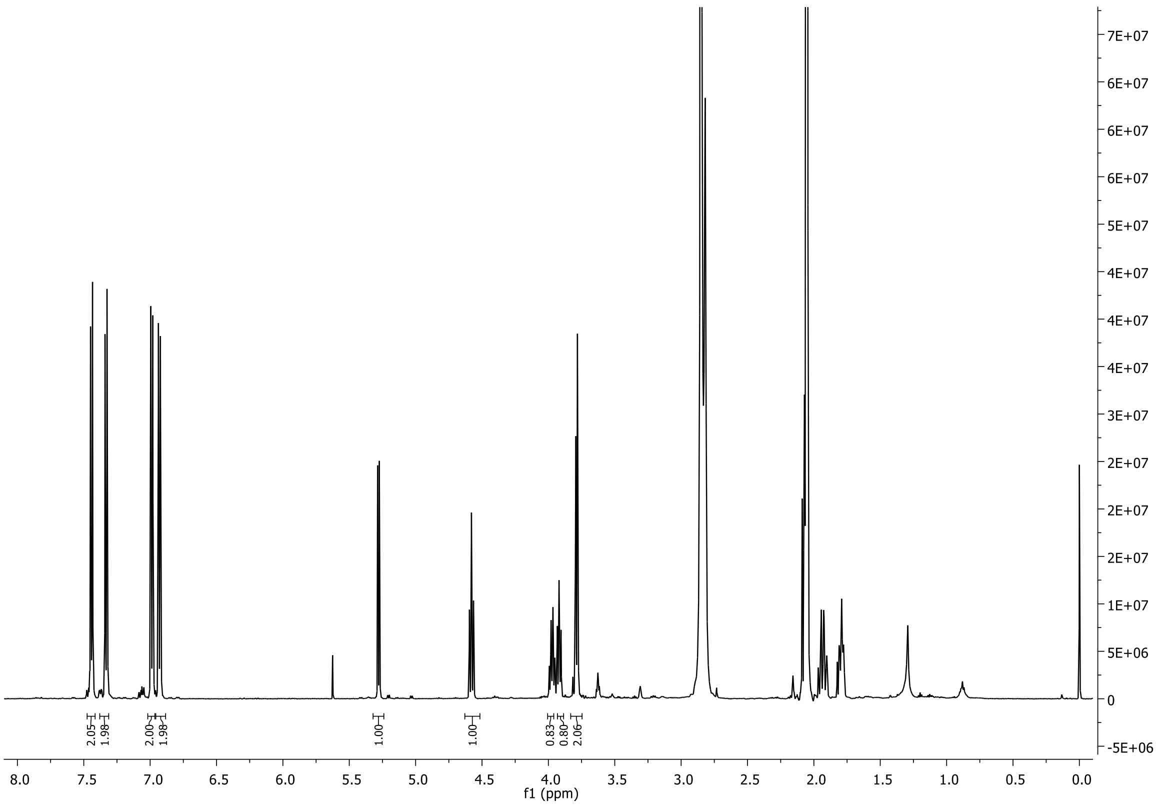

Figure S23. ^{1}H NMR spectrum of **2** (500 MHz, acetone-d$_6$, 298 K).

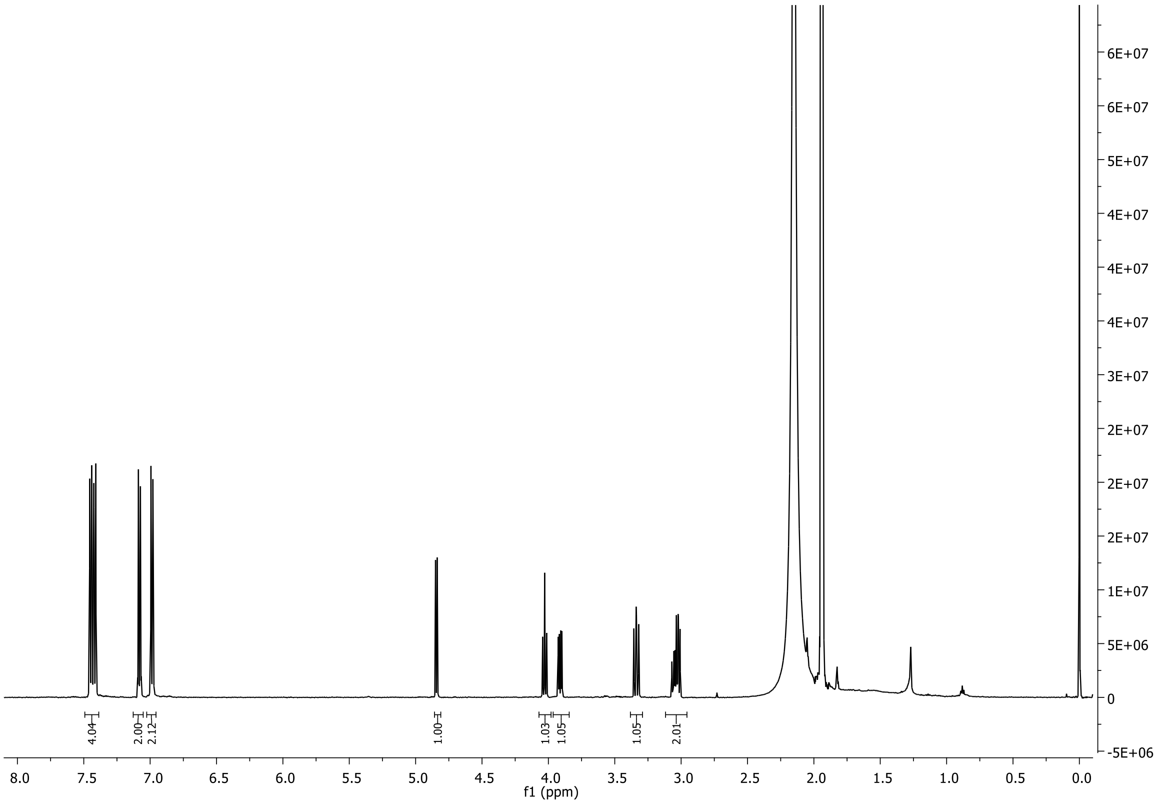

Figure S24. ^{1}H NMR spectrum of **2** (500 MHz, MeCN-d_3, 298 K).

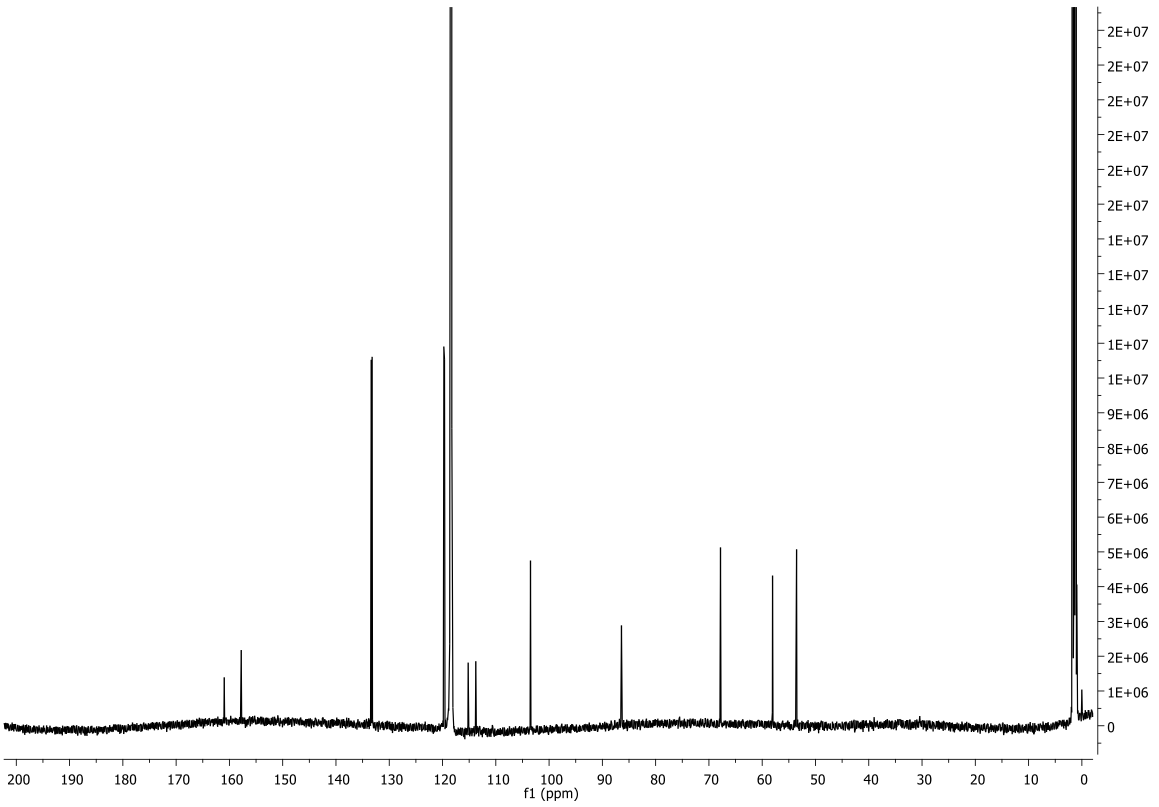

Figure S25. ^{13}C NMR spectrum of **2** (125 MHz, MeCN-d_3, 298 K).

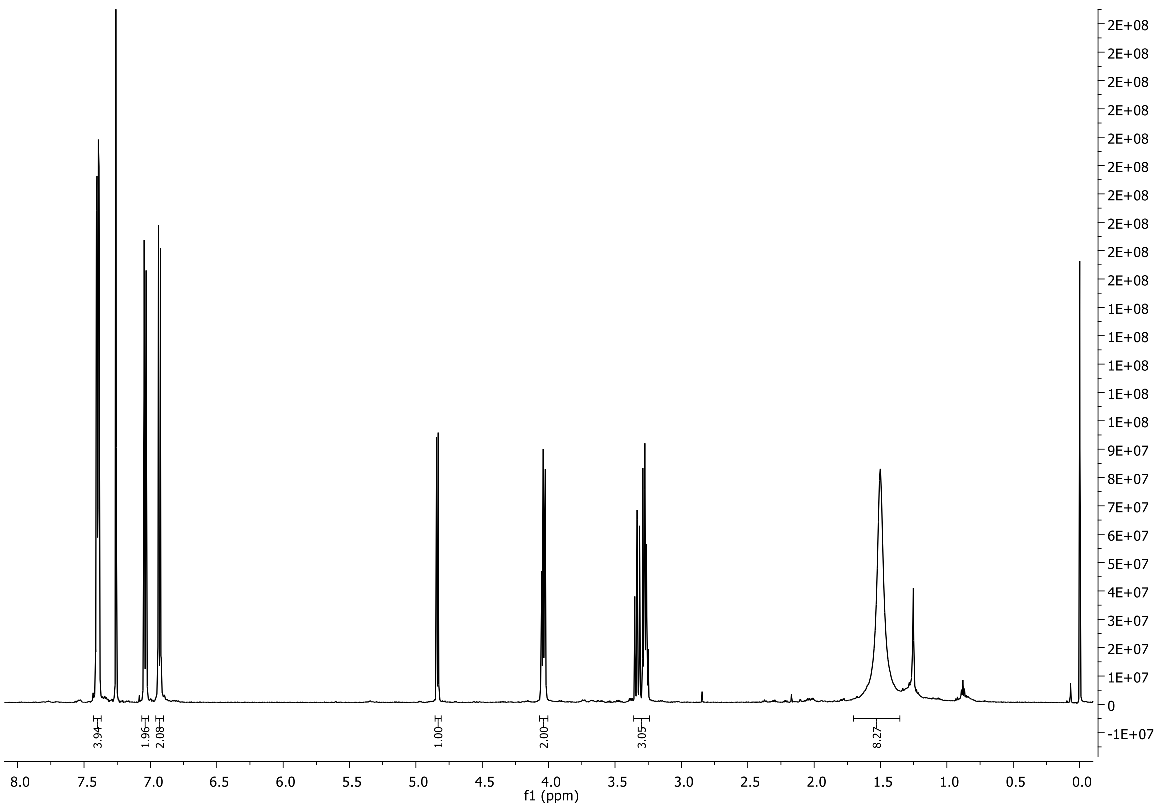

Figure S26. ^{1}H NMR spectrum of **2** (500 MHz, $CDCl_3$, 298 K).

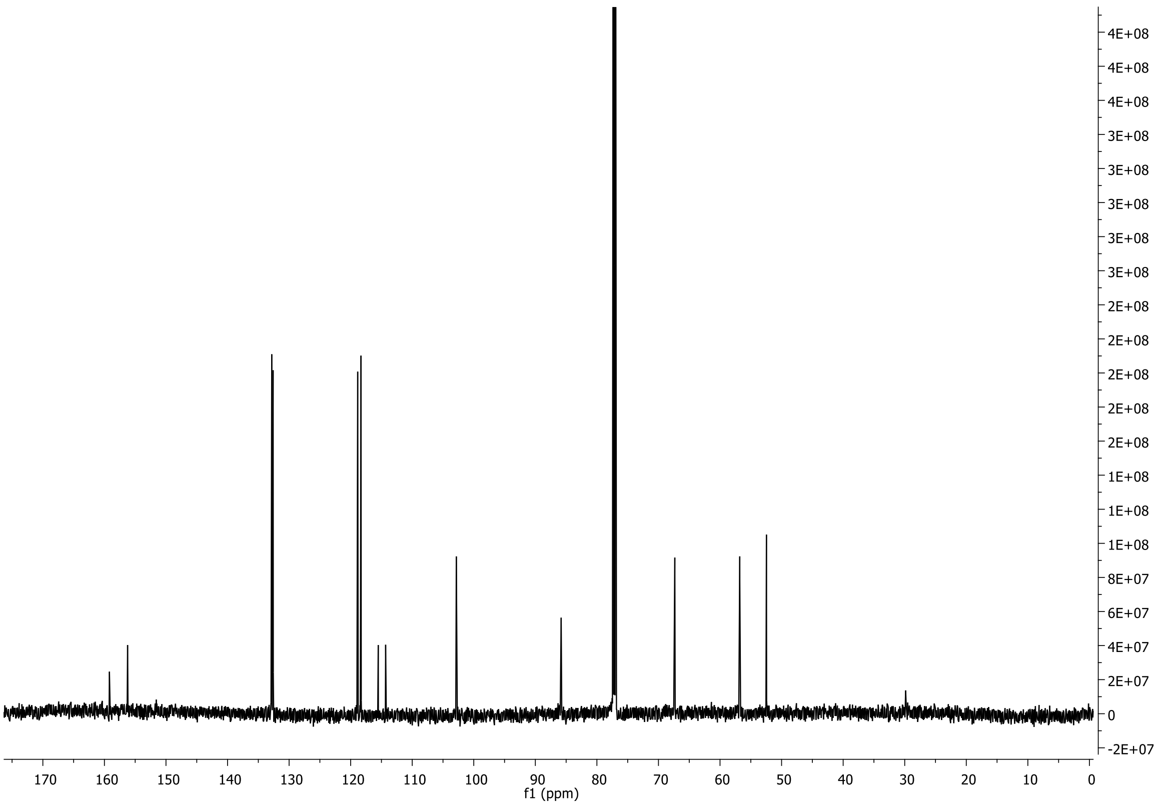

Figure S27. ^{13}C NMR spectrum of **2** (125 MHz, $CDCl_3$, 298 K).

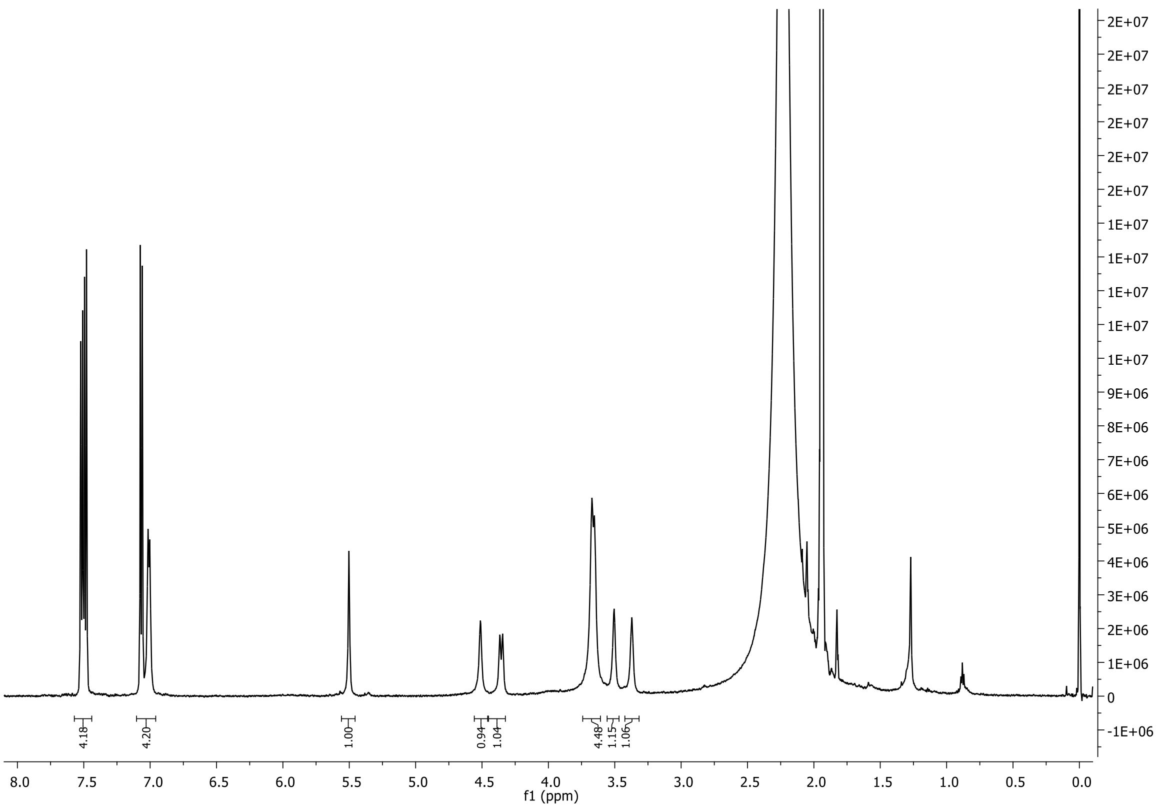

Figure S28. ^{1}H NMR spectrum of **3** (500 MHz, MeCN-d_3, 298 K).

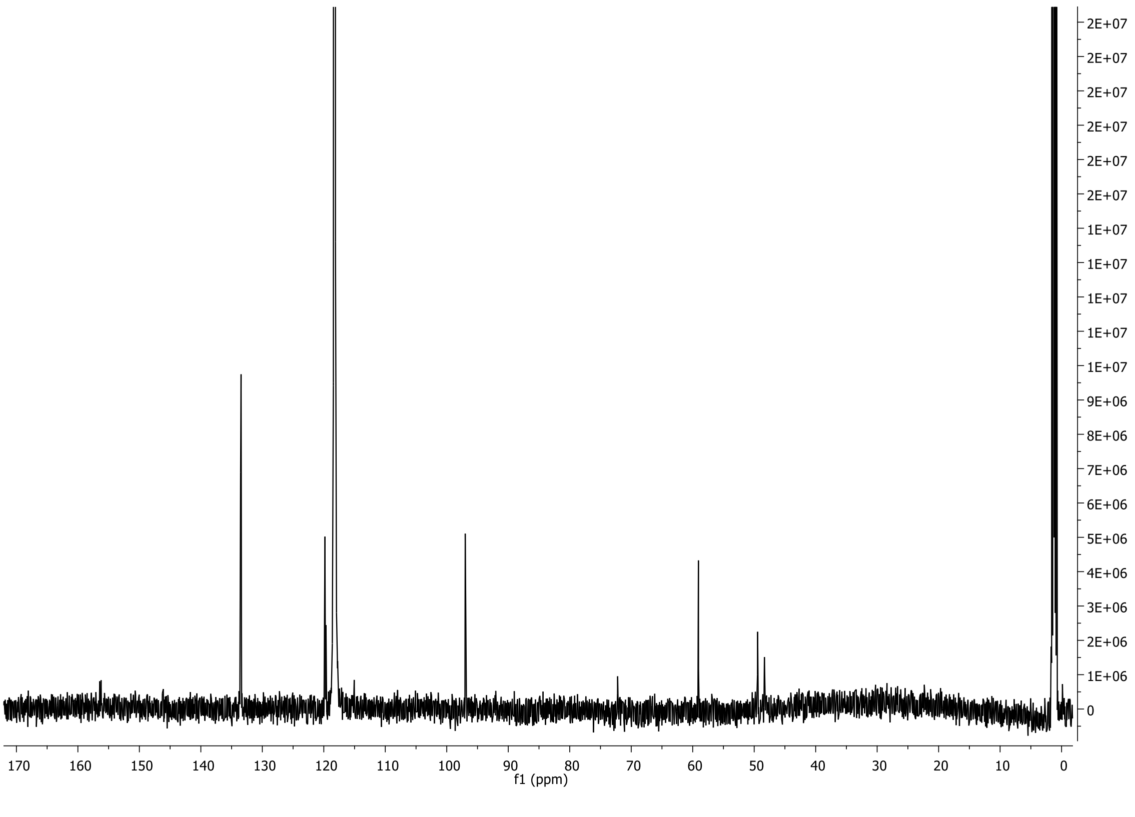

Figure S29. ^{13}C NMR spectrum of **3** (125 MHz, MeCN-d_3, 298 K).

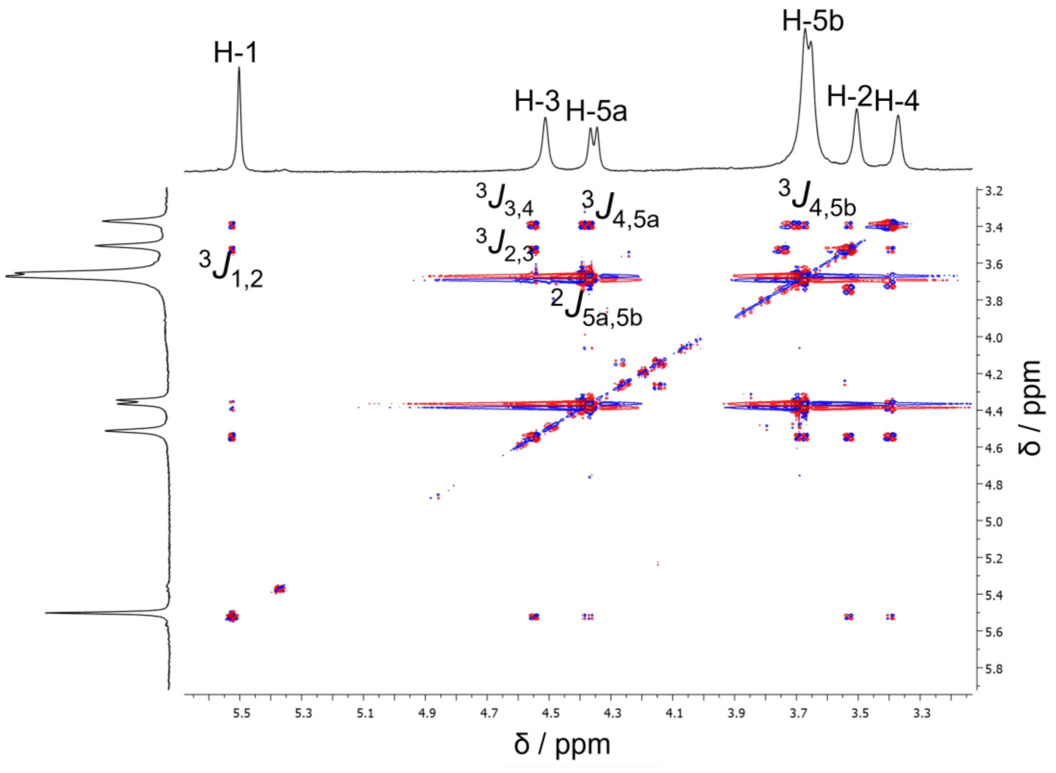

Figure S30. COSY-DQF NMR spectrum of **3** (500 MHz, MeCN-d_3, 298 K).

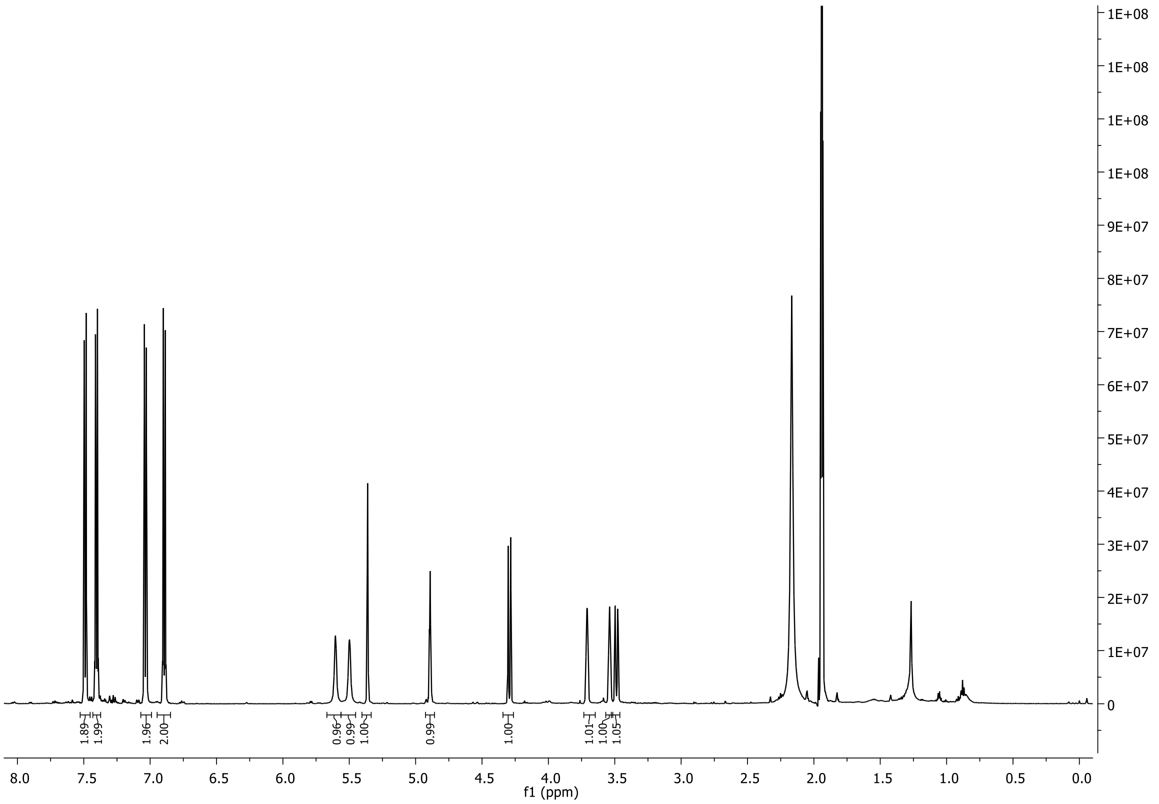

Figure S31. ^{1}H NMR spectrum of **4** (600 MHz, MeCN-d_3, 298 K).

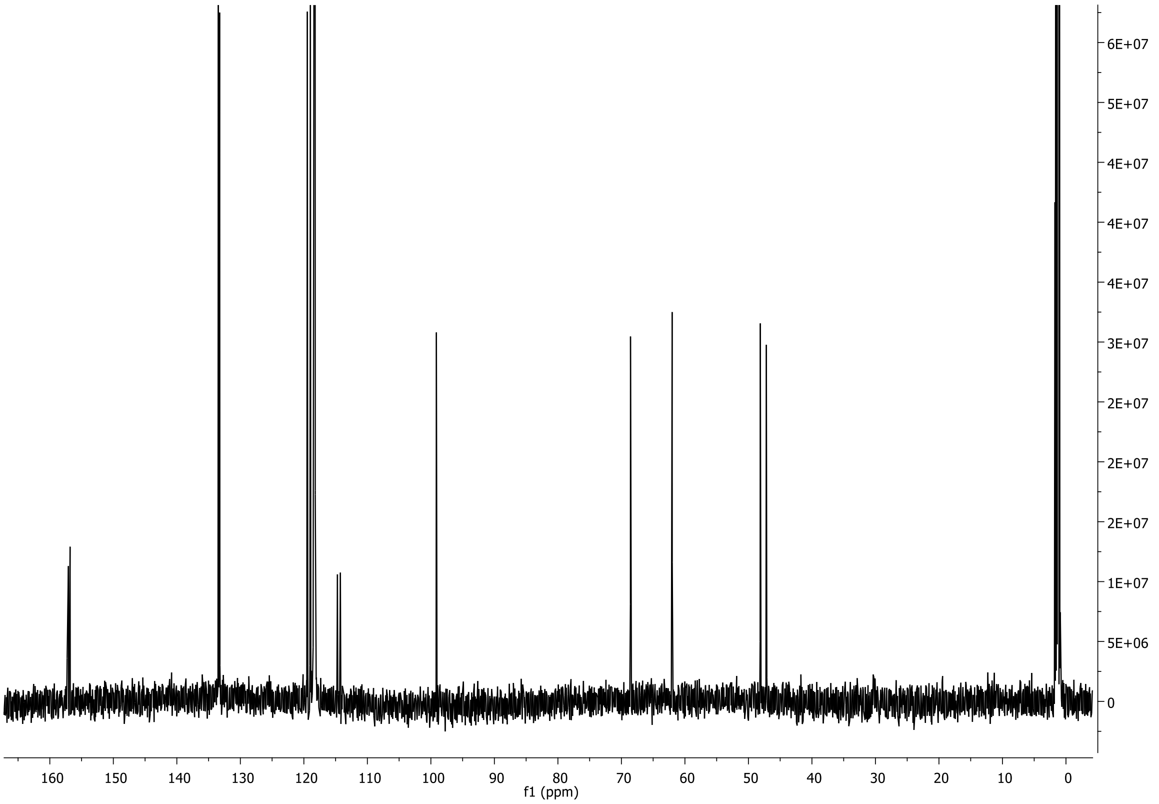

Figure S32. ^{13}C NMR spectrum of **4** (125 MHz, MeCN-d_3, 298 K).

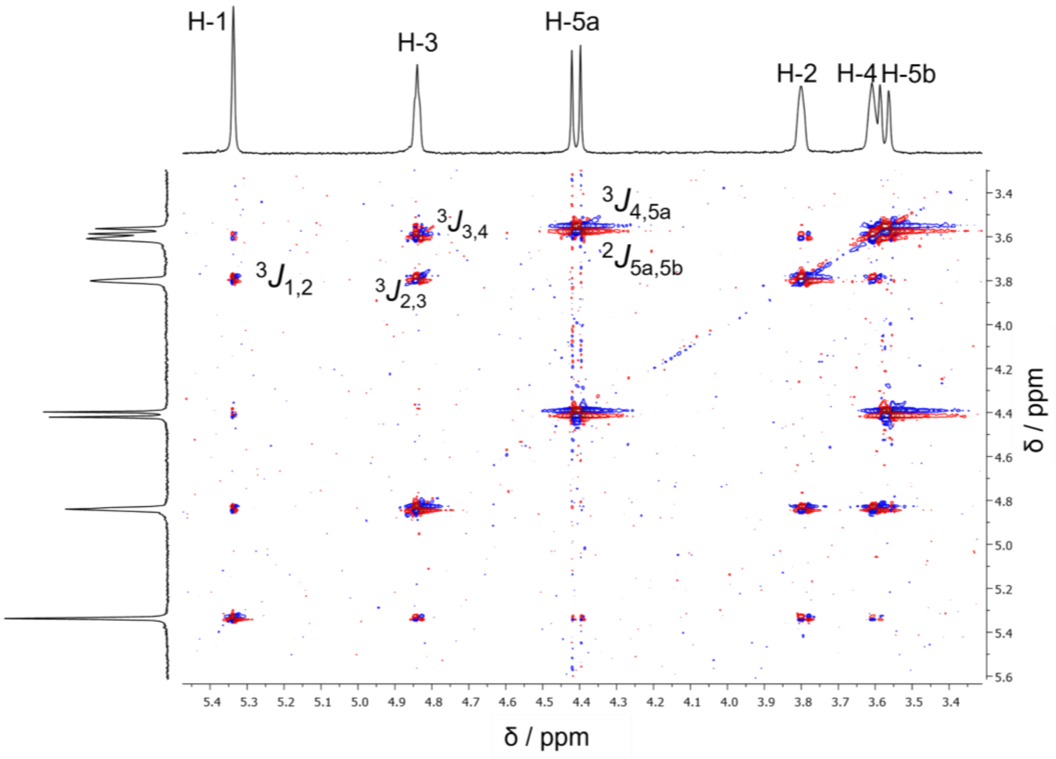

Figure S33. COSY-DQF NMR spectrum of **4** (500 MHz, MeCN-d_3, 298 K).

5 References

[1] A. D. McNaught, *Carbohydr. Res.* **1997**, *297*, 1-92.

[2] S. O. Jaeschke, T. K. Lindhorst, *Eur. J. Org. Chem.* **2021**, *2021*, 6312-6318.

[3] a) F. Neese, *WIREs Comput. Mol. Sci.* **2018**, *8*, e1327; b) F. Neese, F. Wennmohs, U. Becker, C. Riplinger, *J. Chem. Phys.* **2020**, *152*, 224108.

[4] a) A. D. Becke, *Phys. Rev. A* **1988**, *38*, 3098-3100; b) C. Lee, W. Yang, R. G. Parr, *Phys. Rev. B* **1988**, *37*, 785-789; c) A. D. Becke, *J. Chem. Phys.* **1992**, *96*, 2155-2160.

[5] F. Weigend, M. Häser, H. Patzelt, R. Ahlrichs, *Chem. Phys. Lett.* **1998**, *294*, 143-152.

[6] a) S. Grimme, J. Antony, S. Ehrlich, H. Krieg, *J. Chem. Phys.* **2010**, *132*, 154104; b) S. Grimme, S. Ehrlich, L. Goerigk, *J. Comput. Chem.* **2011**, *32*, 1456-1465; c) E. Caldeweyher, S. Ehlert, A. Hansen, H. Neugebauer, S. Spicher, C. Bannwarth, S. Grimme, *J. Chem. Phys*. **2019**, *150*, 154122.

[7] a) N. Mardirossian, M. Head-Gordon, *J. Chem. Phys.* **2016**, *144*, 214110; b) A. Najibi, L. Goerigk, *J. Comput. Chem*. **2020**, *41*, 2562-2572.

[8] F. Jensen, *Theor. Chem. Acc.* **2010**, *126*, 371-382.

[9] J. P. Perdew, K. Burke, M. Ernzerhof, *Phys. Rev. Lett.* **1996**, *77*, 3865-3868.

[10] J. P. Perdew, M. Ernzerhof, K. Burke, *J. Chem. Phys.* **1996**, *105*, 9982-9985.

[11] J. P. Perdew, J. A. Chevary, S. H. Vosko, K. A. Jackson, M. R. Pederson, D. J. Singh, C. Fiolhais, *Phys. Rev. B* **1992**, *46*, 6671-6687.

[12] a) J. Tao, J. P. Perdew, V. N. Staroverov, G. E. Scuseria, *Phys. Rev. Lett.* **2003**, *91*, 146401; b) J. P. Perdew, J. Tao, V. N. Staroverov, G. E. Scuseria, *J. Chem. Phys.* **2004**, *120*, 6898-6911.

[13] a) F. London, *J. Phys. Radium* **1937**, *8*, 397-409; b) H. F. Hameka, *Mol. Phys.* **1958**, *1*, 203-215; c) R. Ditchfield, *J. Chem. Phys.* **1972**, *56*, 5688-5691; d) R. Ditchfield, *Mol. Phys.* **1974**, *27*, 789-807; e) K. Wolinski, J. F. Hinton, P. Pulay, *J. Am. Chem. Soc.* **1990**, *112*, 8251-8260; f) G. L. Stoychev, A. A. Auer, R. Izsák, F. Neese, *J. Chem. Theory Comput.* **2018**, *14*, 619-637; g) G. L. Stoychev, A. A. Auer, J. Gauss, F. Neese, *J. Chem. Phys.* **2021**, *154*, 164110.

[14] F. Jensen, *J. Chem. Theory Comput.* **2015**, *11*, 132-138.

[15] a) F. Neese, A. Hansen, D. G. Liakos, *J. Chem. Phys.* **2009**, *131*, 064103; b) F. Neese, A. Hansen, F. Wennmohs, S. Grimme, *Acc. Chem. Res.* **2009**, *42*, 641-648; c) D. G. Liakos, A. Hansen, F. Neese, *J. Chem. Theory Comput.* **2011**, *7*, 76-87; d) C. Riplinger, F. Neese, *J. Chem. Phys.* **2013**, *138*, 034106; e) C. Riplinger, B. Sandhoefer, A. Hansen, F. Neese, *J. Chem. Phys.* **2013**, *139*, 134101; f) C. Riplinger, P. Pinski, U. Becker, E. F. Valeev, F. Neese, *J. Chem. Phys.* **2016**, *144*, 024109; g) Y. Guo, C. Riplinger, U. Becker, D. G. Liakos, Y. Minenkov, L. Cavallo, F. Neese, *J. Chem. Phys.* **2018**, *148*, 011101.

[16] T. H. Dunning, *J. Chem. Phys.* **1989**, *90*, 1007-1023.

[17] a) C. Bannwarth, S. Ehlert, S. Grimme, *J. Chem. Theory Comput.* **2019**, *15*, 1652-1671; b) S. Grimme, *J. Chem. Theory Comput.* **2019**, *15*, 2847-2862; c) P. Pracht, F. Bohle, S. Grimme, *Phys. Chem. Chem. Phys.* **2020**, *22*, 7169-7192.

[18] a) M. Hricovíni, O. L. Malkina, F. Bízik, L. T. Nagy, V. G. Malkin, *J. Phys. Chem. A* **1997**, *101*, 9756-9762; b) O. L. Malkina, M. Hricovíni, F. Bízik, V. G. Malkin, *J. Phys. Chem. A* **2001**, *105*, 9188-9195.
